# Supplementary material for: Reductive cyanation of organic chlorides using CO2 and NH3 via Triphos–Ni(I) species
Source: Nat Commun. 2020 Aug 14;11:4096. doi: 10.1038/s41467-020-17939-2 (PMC7428002; doi:10.1038/s41467-020-17939-2)
Supplement: Supplementary file 1 — Supplementary Information [file 41467_2020_17939_MOESM1_ESM.pdf]

Supplementary Information

Reductive Cyanation of Organic Chlorides Using CO_2 and NH_3 via Triphos-Ni(I) Species

Dong et al.

Supplementary Methods

General information

Air- and moisture-sensitive syntheses were performed under argon atmosphere. All chemicals were purchased from Adamas, Aldrich, TCI, Alfa etc. Unless otherwise noted, all commercial reagents were used without further purification. ^{1}H and ^{13}C NMR spectra were recorded on Bruker AVANCE NEO (400 MHz for ^{1}H and 100 MHz for ^{13}C). Chemical shift δ (ppm) was given relative to solvent: references for $CDCl_3$ were 7.26 ppm (^{1}H-NMR) and 77.0 ppm (^{13}C-NMR), and for DMSO-d_6 were 2.50 ppm (^{1}H-NMR) and 39.60 ppm (^{13}C-NMR). ^{13}C-NMR spectra were acquired on a broad band decoupled mode. Data are reported as follows: chemical shift, multiplicity (s = singlet, d = doublet, dd = doublet of doublets, t = triplet, q = quartet, m = multiplet), coupling constant (Hz), and integration. Gas chromatographic analyses were performed on SHIMADZU GC-2010 Plus spectrometer. GC-MS was obtained using electron ionization (SHIMADZU GCMS-QP2010SE). ESI (electrospray ionization) high resolution mass spectra were recorded on an Agilent Technologies 6530 Q-TOF LC/MS spectrometer. TLC was performed using commercially prepared 100-400 mesh silica gel plates (GF254), and visualization was effected at 254 nm.

Experimental procedures

General procedure for the cyanation of aryl chlorides with CO_2/NH_3:

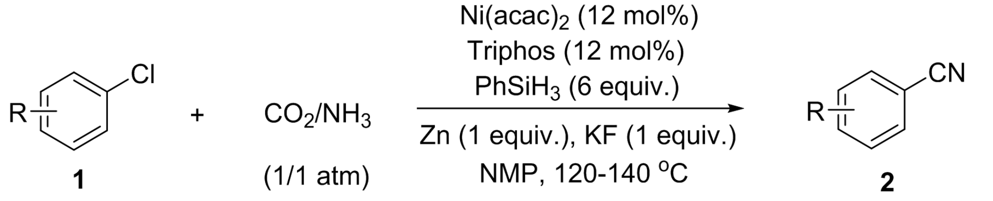

(1)

Under nitrogen atmosphere, $Ni(acac)_2$ (12 mol%, 0.015 mmol), Triphos (12 mol%, 0.015 mmol), KF (1.0 equiv., 0.125 mmol), Zn (1.0 equiv., 0.125 mmol) and a stirring bar were added into a 10 mL oven-dried sealed tube (Supplementary Figure 1). Then NMP (0.5 mL), aryl chlorides (1.0 equiv., 0.125 mmol) and $PhSiH_3$ (6.0 equiv., 0.75 mmol) were injected by syringe. The tube was sealed and CO_2 (3.6 equiv., 10 mL) as well as NH_3 (3.6 equiv., 10 mL) were injected by syringe after N_2 was removed under vacuum. Then the mixture was stirred for 20 h in a pre-heated alloyed block. After the reaction finished, the tube was cooled to room temperature and the pressure was carefully released. The yield was measured by GC analysis or isolated by preparative thin-layer chromatography on silica gel plates.

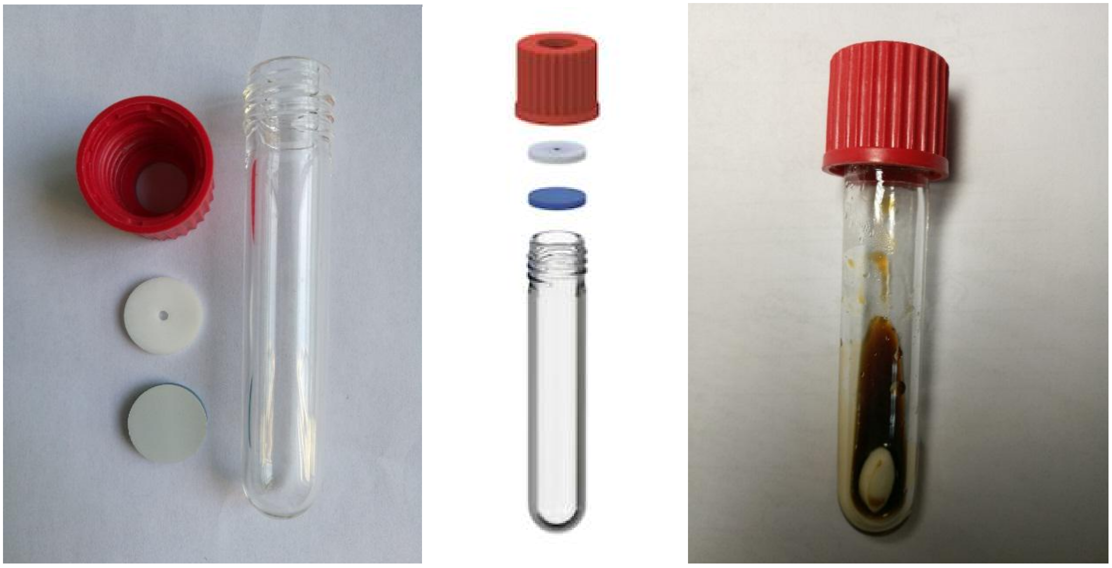

Supplementary Figure 1. The sealed glass tube used in reactions of aryl halides with CO_2 and NH_3

General procedure for the cyanation of aryl chlorides with urea:

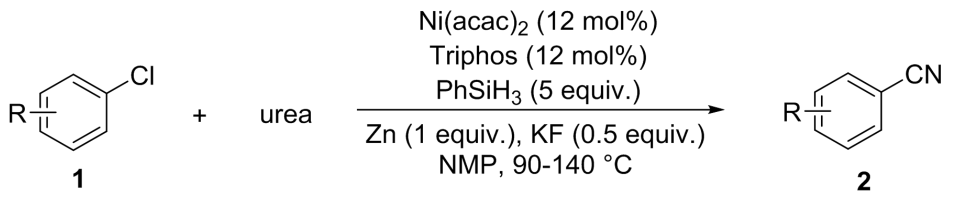

(2)

Under nitrogen atmosphere, $Ni(acac)_2$ (12 mol%, 0.036 mmol), Triphos (12 mol%, 0.036 mmol), urea (3.0 equiv., 0.9 mmol), KF (0.5 equiv., 0.15 mmol), Zn (1.0 equiv., 0.3 mmol) and a stirring bar were added into a 15 mL oven-dried sealed tube. Then NMP (1 mL), aryl chlorides (1.0 equiv., 0.3 mmol) and $PhSiH_3$ (5.0 equiv., 1.5 mmol) were injected by syringe. The tube was then sealed and the mixture was stirred for 20 h in a pre-heated alloyed block. After the reaction finished, the mixture was diluted with EtOAc (20 mL) and filtered through a short plug of celite. The filtrate was washed with H_2O (3 × 20 mL) and dried by anhydrous Na_2SO_4. Then the crude product was concentrated under reduced pressure, and the residue was purified by preparative thin-layer chromatography on silica gel plates eluting with PE/EtOAc to afford corresponding product.

Cell proliferation assay

MCF-7 and Hela cells were cultured in a 96-well plate at a density of 3500–5000 cells/well and were maintained at 37 °C in a humidified atmosphere of 5% CO_2 for 24 h. Subsequently, serial dilutions of the compounds (the same amount of DMSO was used as control) were added in the plates for 48 h. The cell proliferation was determined by CCK8 kit according to the protocol (Apexbio Technology LLC).

Supplementary Table 1

Screening of catalysts.[a]

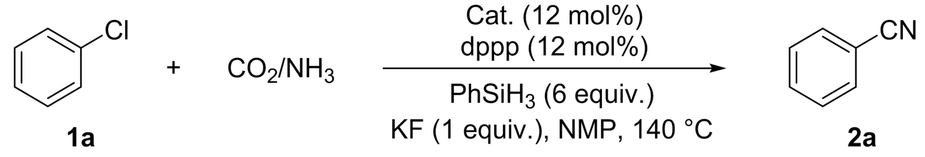

Entry	Cat. (mol%)	Yield (%)
1	$PdCl_2$ (5)	trace
2	$Pd(OAc)_2$ (5)	trace
3	$Pd(PPh_3)_4$ (5)	n.d.
4	$[Rh(COD)_2]BF_4$ (5)	n.d.
5	$[Rh(OAc)_2]_2$ (5)	n.d.
6	$FeCl_2$	n.d.
7	$Fe(OTf)_3$	n.d.
8	$Co(acac)_2$	n.d.
9	$Co(OH)_2$	n.d.
10	$CuFe_2O_4$	n.d.
11	Cu_2O	n.d.
12	CuO	n.d.
13	CuF_2	n.d.
14	$CuCl_2$	n.d.
15	CuI	n.d.
16	$Cu(acac)_2$	n.d.
17	$Cu(OAc)_2$	n.d.
18	$Ni(COD)_2$	2
19	$Ni(OTf)_2$	5
20	$NiBr_2$	17
21	$NiBr_2$.glyme	17
22	$Ni(acac)_2$	20

[a]Reaction conditions: **1a** (0.125 mmol), CO_2/NH_3 (1/1 atm) and NMP (0.5 mL), 140 °C, 20 h. The yield was determined by GC analysis using dodecane as the internal standard. COD: 1,5-Cyclooctadiene.

Supplementary Table 2

Screening of ligands and additives. [a]

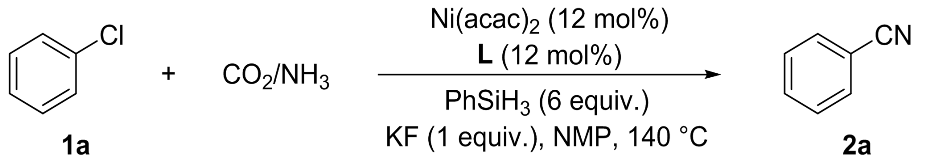

Entry	Ligand	Additive	Yield (%)
1	2,2'-Bipyridine	-	n.d.
2	*o*-Phen	-	n.d.
3	Bathocuproine	-	n.d.
4	DABCO	-	n.d.
5	DMEDA	-	n.d.
6	TMEDA	-	n.d.
7	(+/-)-trans-1,2-Diaminocyclohexane	-	n.d.
8	Diphenyl-2-pyridylphosphine	-	n.d.
9	PPh_3	-	n.d.
10	$PhPCy_2$	-	n.d.
11	PCy_3	-	n.d.
12	dppp	-	20
13	dppp	Zn (1.0 eq.)	37
14	dppe	Zn (1.0 eq.)	4
15	dppb	Zn (1.0 eq.)	5
16	Xantphos	Zn (1.0 eq.)	3
17	dppf	Zn (1.0 eq.)	10
18	Tripod	Zn (1.0 eq.)	64
19	dppp	Mn (1.0 eq.)	31
20	dppp	Mg (1.0 eq.)	29
21	dppp	$MgCl_2$ (1.0 eq.)	18
22	dppp	$ZnCl_2$ (1.0 eq.)	23
23	Triphos	Zn (1.0 eq.)	81

24[b]	Triphos	Zn (1.0 eq.)	79
25[c]	Triphos	Zn (1.0 eq.)	42
26[d]	Triphos	Zn (1.0 eq.)	21
27[e]	Triphos	Zn (1.0 eq.)	trace
28[f]	Triphos	Zn (1.0 eq.)	trace
29[g]	Triphos	Zn (1.0 eq.)	trace
30[h]	Triphos	Zn (1.0 eq.)	trace
31[i]	Triphos	Zn (1.0 eq.)	5

[a]Reaction conditions: **1a** (0.125 mmol), CO_2/NH_3 (1/1 atm) and NMP (0.5 mL), 140 °C, 20 h. The yield was determined by GC analysis using dodecane as the internal standard. [b]K_3PO_4 as base. [c]KO^tBu as base. [d]KOH as base. [e]DCE as solvent. [f]THF as solvent. [g]1,4-Dioxane as solvent. [h]Toluene as solvent. [i]PMHS as silane. NMP: N-methyl-2-pyrrolidone; DABCO: 1,4-Diazabicyclo[2.2.2]octane; DMEDA: N,N'-Dimethyl-1,2-ethanediamine; TMEDA: N,N,N',N'-Tetramethylethylenediamine; dppp: 1,3-Bis(diphenylphosphino)propane; dppe: 1,2-Bis(diphenylphosphino)ethane; dppb: 1,4-Bis(diphenylphosphino)butane, dppf: 1,1'-Bis(diphenylphosphino)ferrocene, Tripod: 1,1,1-Tris(diphenylphosphinoMethyl)ethane; Triphos: Bis(2-diphenylphosphinoethyl)phenylphosphine; PMHS: poly(methylhydrosiloxane); n.d.: Not detected.

Synthesis of nickel complexes

1) $[Ni(dppp)_2]BF_4$ Bis[1,3-Bis(diphenylphosphino)propane] nickel(I) tetrafluoroborate

$$\underset{\textbf{S1}}{[Ni(CH_3CN)_6](BF_4)_2} + dppp + \underset{\textbf{S2}}{Ni(dppp)_2} \longrightarrow \underset{\textbf{S3}}{[Ni(dppp)_2]BF_4} \quad (3)$$

$[Ni(dppp)_2]BF_4$ (**S3**) was prepared following the literature procedure.[1] A mixture of **S1** (195 mg, 0.4 mmol) and dppp (337 mg, 0.8 mmol) in acetonitrile (20 mL) was added dropwise into the acetonitrile solution (20 mL) of **S2** (355 mg, 0.4 mmol). And the resulting mixture was stirred at 25 ºC for 20 h. After reaction, the resulting yellow solution was concentrated under vacuum until cloudy. Then the suspension was filtered and washed with heptane to yield a fine yellow powder. The crude product was collected and re-crystallized from a mixture of dichloromethane and heptane to yield (160 mg 41%) as a brown solid.

$[Ni(CH_3CN)_6](BF_4)_2$ (**S1**) was prepared following the literature procedure using Ni and $NOBF_4$.[2] $Ni(dppp)_2$ (**S2**) was prepared following the literature procedure using $Ni(COD)_2$ and 1,3-Bis(diphenylphosphino)propane (dppp).[3]

2) *o*-tolylNi(dppf)Cl [1,1'-bis(diphenylphosphino)ferrocene](2-methylphenyl)nickel(II) chloride

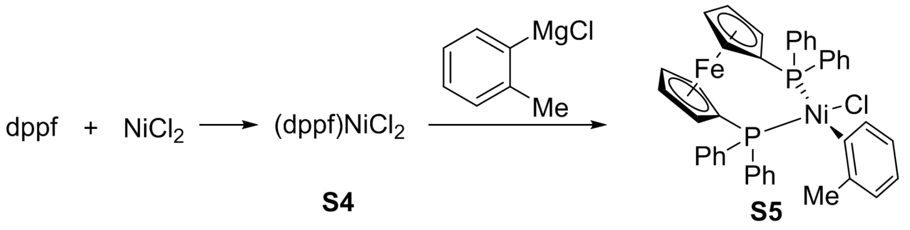

(4)

o-tolylNi(dppf)Cl (**S5**) was prepared following the literature procedure.[4-5]

$NiCl_2$ (4 mmol, 530 mg), EtOH (15 mL) and a magnetic stir bar were placed in a 50 mL two-neck round bottomed flask fitted with a refluxing condenser. The solution was stirred at room temperature in nitrogen for 15 min. Then dppf (4 mmol, 2310 mg) was added in one portion. The reaction mixture was heated to 80 °C for 1 h and then cooled to room temperature. Once cool, the flask was chilled to 0 °C for 10 min, after which the solid was collected by filtration, washed twice with ethanol (10 mL) and twice with ether (10 mL). Drying under vacuum yielded **S4** (2600 mg, 95 %) as a deep green solid.

Under argon atmosphere, **S4** (3.0 mmol, 2052 mg) and THF (100.0 mL) were placed in a round bottomed flask. The resulting suspension was cooled to 0 ºC in an ice bath, and then *o*-tolylmagnesium chloride (1.4 M in THF/toluene 82/18, 4.5 mmol, 3.2 mL) was added dropwise with vigorous stirring. The reaction mixture was stirred for 2 h at 0 ºC, then quenched with methanol (2.0 mL), stirred for 30 min at ambient temperature, filtered through celite and washed with THF. The solvent was evaporated under reduced pressure, then methanol (10.0 mL) was added and the mixture was sonicated until a uniform suspension was obtained. After the suspension was cooled to 0 ºC, the precipitate was collected by filtration, washed with cold methanol, cold ether, a small amounts of THF and dried under vacuum. Then the solid was dissolved in CH_2Cl_2, filtered through celite, and washed with CH_2Cl_2. The solvent was evaporated at 30 ºC under reduced pressure, then methanol (5.0 mL) was added and the mixture was sonicated until a uniform suspension was obtained. After the suspension was cooled to 0 ºC, the yellow precipitate was collected by filtration, washed with cold methanol and ether, and dried under

high vacuum to afford the product *o*-tolylNi(dppf)Cl (**S5**) in 33% yield (730 mg) as a bright yellow solid. HRMS (ESI): Calcd. for $C_{41}H_{35}FeNiP_2^+$ [M-Cl]$^+$: 703.0917, Found: 703.0924.

3) Ni(dppp)Br_3 [1,3-Bis(diphenylphosphino)propane]nickel(III) bromide

Ni(dppp)Br_3 (**S6**) was prepared following the literature procedure using Ni(dppp)Br_2 and Br_2.[6]

Investigation of the reaction mechanism

1) Cyanation of aryl chlorides with trimethylsilyl cyanide (TMS-CN)

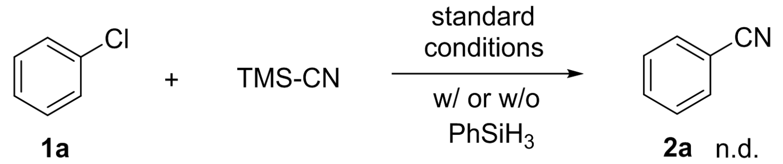

(5)

Upon exposure of TMS-CN to standard conditions (with or without $PhSiH_3$), no product **2a** was detected while substrate **1a** was mostly recovered. Such result suggested that CN^- is not involved in the reaction mechanism.

2) Detection of cyanide anion by indicator paper[7-9]

Upon exposure of cyanide indicator paper to reaction mixture (after the reaction was finished), the test paper didn't changed color. Such experiment indicated that no detectable CN^- was produced in the reaction mechanism.

3) Cyanation of aryl chlorides using $^{13}CO_2$

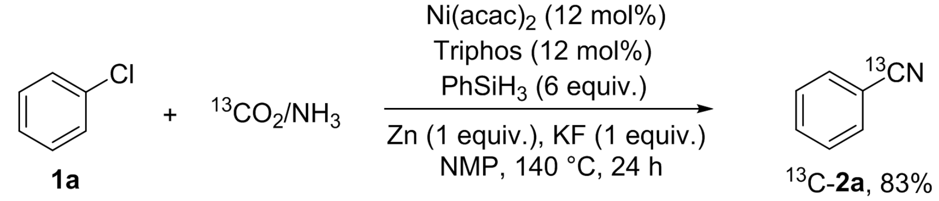

(6)

$^{13}CO_2$ instead of CO_2 was used at standard conditions. After the reaction finished, dodecane was added. And the mixture was diluted with EtOAc and quenched with aqueous solution of NaOH (2M). Stir completely about 30 minutes and leave it for 60 minutes to ensure the solution is stratified. Then, the organic layer was tested with GC-MS and NMR.

GC-MS analysis of reaction mixtures indicated that the exact mass of target product is 103.95. This result showed that the carbon source of CN moiety derives from CO_2. And NMR results (118.74; obvious signal peaks of CN) of reaction mixtures were consistent with GC-MS analysis (Supplementary Figure 2 and 3).

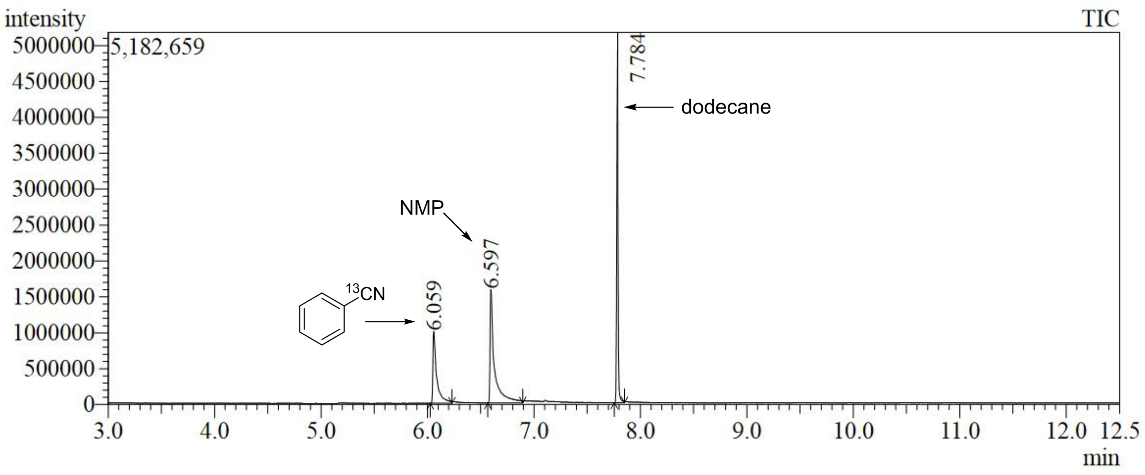

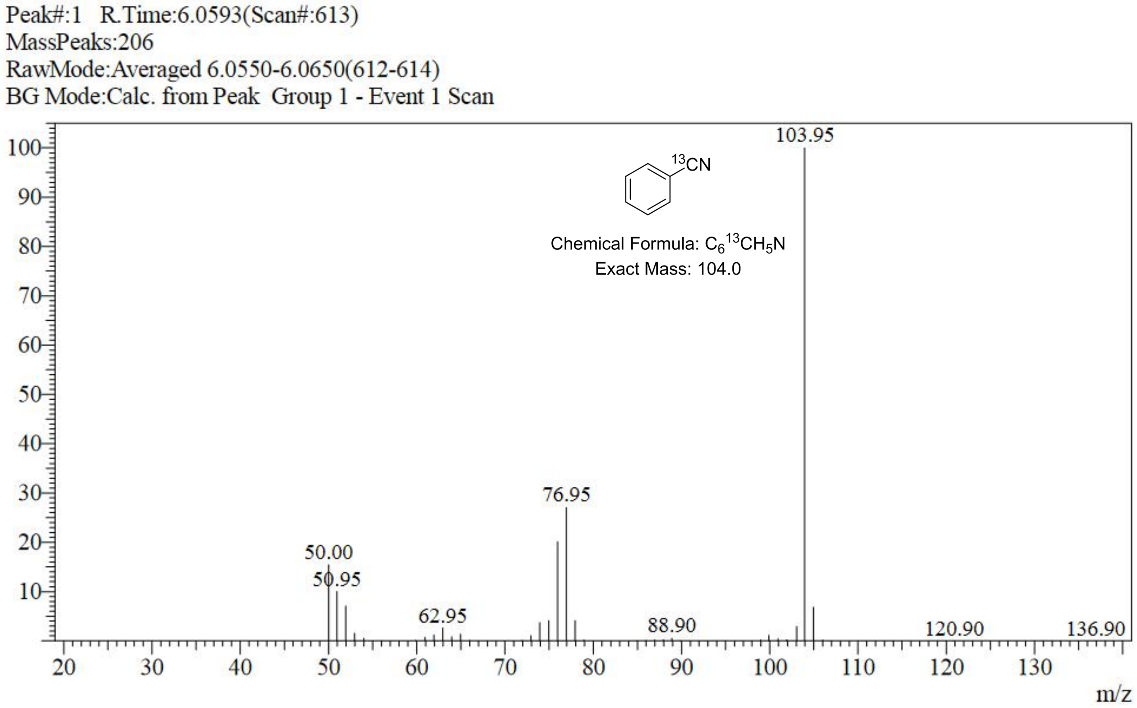

Supplementary Figure 2. GC-MS report of reaction mixtures using $^{13}CO_2$

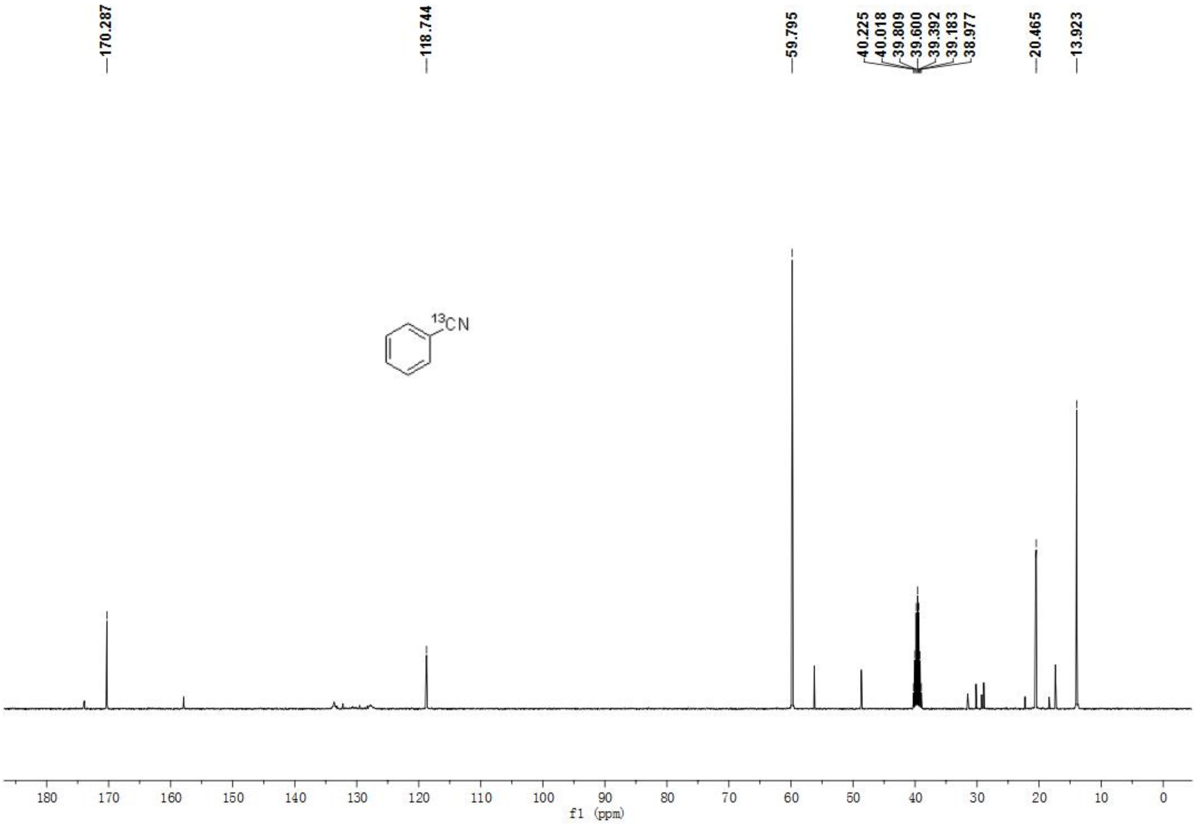

Supplementary Figure 3. NMR spectra of reaction mixtures using $^{13}CO_2$

4) Cyanation of aryl chlorides with Et_3SiH

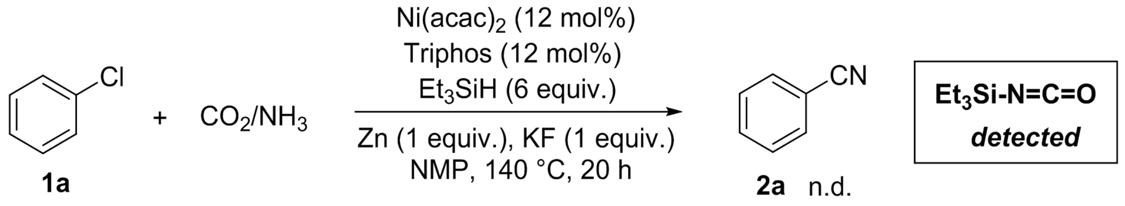

(7)

In the presence of Et_3SiH, no product **2a** was detected. Meanwhile, gas chromatography-mass spectrometry (GC-MS) analysis of reaction mixtures exhibited triethylsilyl isocyanates (Supplementary Figure 4).

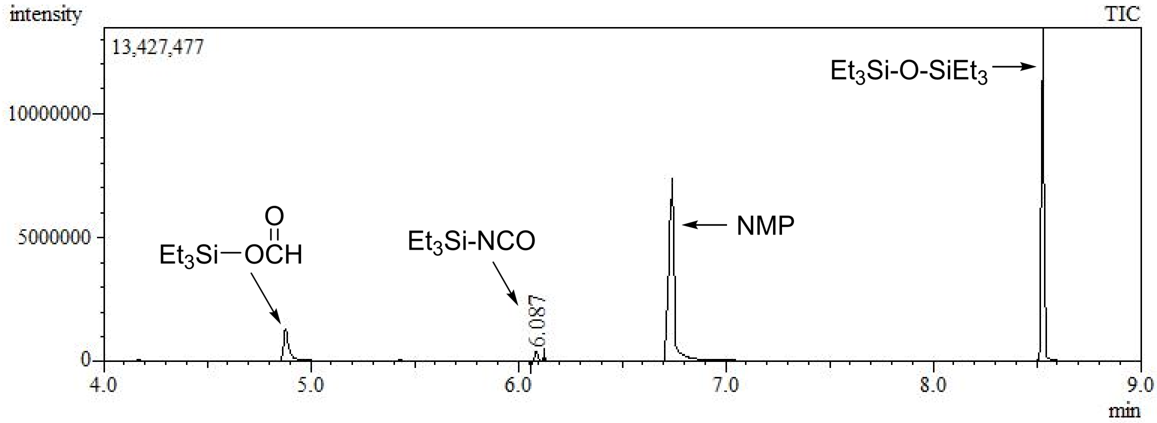

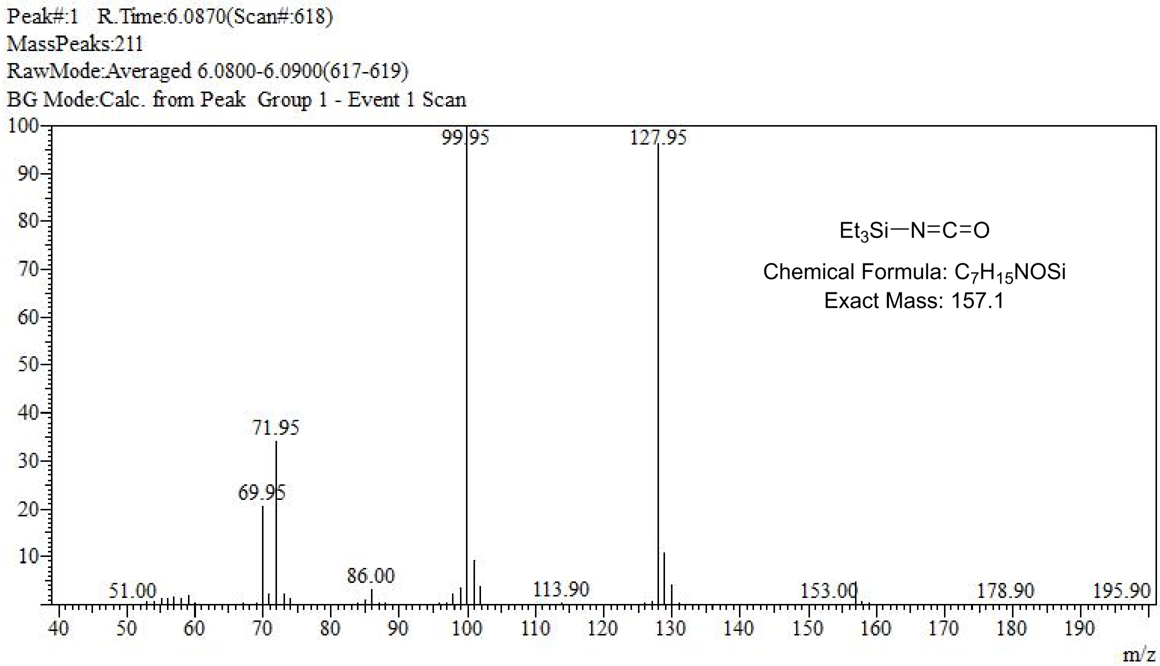

Supplementary Figure 4. GC-MS report of cyanation of aryl chlorides with Et_3SiH

5) Cyanation of chlorobenzene with other C(+4) compounds[a]

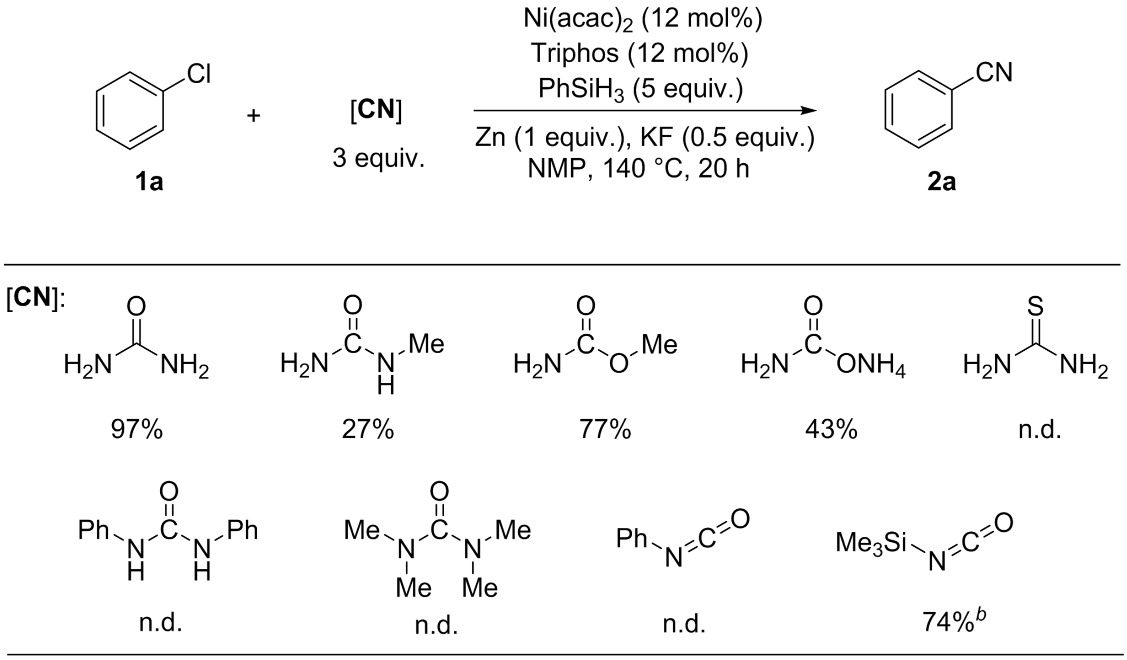

[a]Reaction conditions: 0.3 mmol scale; GC yields. [b]2 equiv. $PhSiH_3$.

Under nitrogen atmosphere, $Ni(acac)_2$ (12 mol%, 0.036 mmol), Triphos (12 mol%, 0.036 mmol), C(+4) compound (3.0 equiv.), KF (0.5 equiv., 0.15 mmol), Zn (1.0 equiv., 0.3 mmol) and a stirring bar were added into a 15 mL oven-dried sealed tube. Then NMP (1 mL), aryl chlorides (1.0 equiv., 0.3 mmol), and $PhSiH_3$ (5.0 equiv., 1.5 mmol) were injected by syringe. The tube was then sealed and the mixture was stirred for 20 h in a pre-heated-to-140 °C alloyed block. After the reaction finished, the tube was cooled to room temperature and the pressure was carefully released. The yield and was measured by GC analysis using dodecane as the internal standard.

6) Cyanation of arylnickel(I) with trimethylsilyl isocyanate (TMS-NCO)

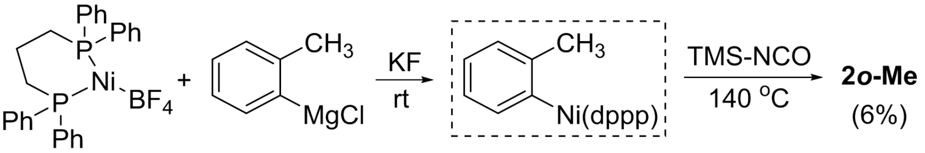

(8)

Under nitrogen atmosphere, $[Ni(dppp)_2]BF_4$ (1.0 equiv., 0.1 mmol), KF (1.0 equiv., 0.1 mmol) and a stirring bar were added into a 15 mL oven-dried sealed tube. Then NMP (1 mL) and *o*-tolylmagnesium chloride (1.0 equiv., 0.1 mmol, 1.4 M in THF/toluene 82/18) were injected by syringe. The tube was then sealed and the mixture was stirred for 1 h at room temperature. After this, TMS-NCO (2.0 equiv., 0.2 mmol) was injected by syringe and the mixture was stirred for 20 h at 140 °C. After the reaction finished, the tube was cooled to room temperature and the pressure was carefully released. The result was obtained by GC and GC-MS analysis.

7) Cyanation of arylnickel(II) with trimethylsilyl isocyanate (TMS-NCO)

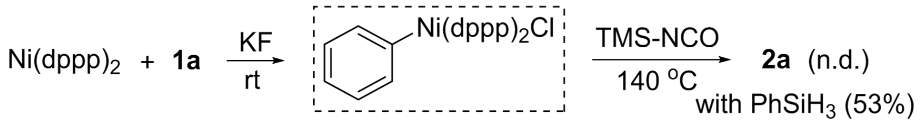

(9)

Under nitrogen atmosphere, $Ni(dppp)_2$ (1.0 equiv., 0.1 mmol), KF (1.0 equiv., 0.1 mmol) and a stirring bar were added into a 15 mL oven-dried sealed tube. Then NMP (1 mL) and chlorobenzene (1.0 equiv., 0.1 mmol) were injected by syringe. The tube was then sealed and the mixture was stirred for 1 h at room temperature. After this, TMS-NCO (2.0 equiv., 0.2 mmol) was injected by syringe and the mixture was stirred for 20 h at 140 °C. After the reaction finished, the tube was cooled to room temperature and the pressure was carefully released. The result was obtained by GC and GC-MS analysis.

8) Direct cyanation of arylnickel(II) with trimethylsilyl isocyanate (TMS-NCO)

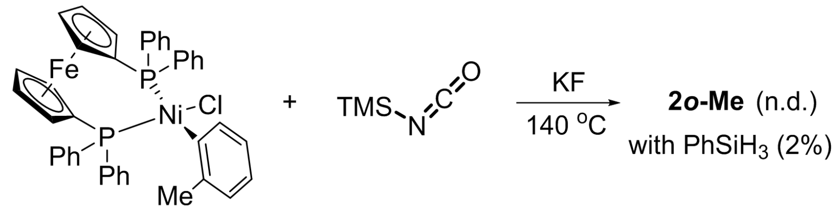

(10)

Under nitrogen atmosphere, *o*-tolylNi(dppf)Cl (1.0 equiv., 0.1 mmol), KF (1.0 equiv., 0.1 mmol) and a stirring bar were added into a 15 mL oven-dried sealed tube. Then NMP (1 mL) and TMS-NCO (2.0 equiv., 0.2 mmol) was injected by syringe and the mixture was stirred for 20 h at 140 °C. After the reaction finished, the tube was cooled to room temperature and the pressure was carefully released. The result was obtained by GC and GC-MS analysis.

9) Cyanation of arylnickel(III) with trimethylsilyl isocyanate (TMS-NCO)

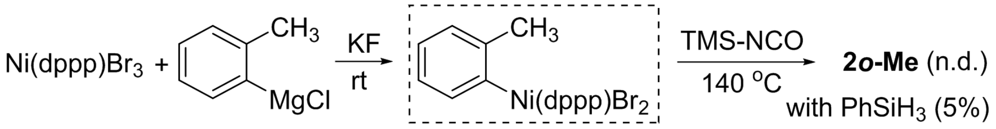

(11)

Under nitrogen atmosphere, $Ni(dppp)Br_3$ (1.0 equiv., 0.1 mmol), KF (1.0 equiv., 0.1 mmol) and a stirring bar were added into a 15 mL oven-dried sealed tube. Then NMP (1 mL) and *o*-tolylmagnesium chloride (1.0 equiv., 0.1 mmol, 1.4 M in THF/toluene 82/18) were injected by syringe. The tube was then sealed and the mixture was stirred for 1 h at room temperature. After this, TMS-NCO (2.0 equiv., 0.2 mmol) was injected by syringe and the mixture was stirred for 20 h at 140 °C. After the reaction finished, the tube was cooled to room temperature and the pressure was carefully released. The result was obtained by GC and GC-MS analysis.

6. X-Ray diffraction characterization

Single crystal of **2aa** was obtained by recrystallization in EA. CCDC: 1974249 contains the supplementary crystallographic data which can be obtained free of charge from the Cambridge Crystallography Data Center via www.ccdc.cam.ac.uk/data_request/cif.

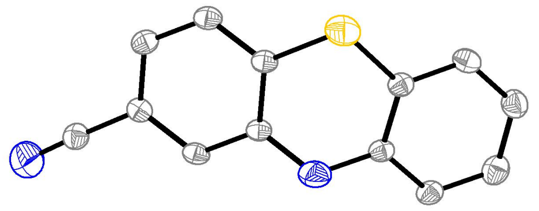

Supplementary Figure 5. Thermal Ellipsoid Depiction of Compound **2aa**

Supplementary Table 3

Crystal data and structure refinement for compound **2aa**

	10*H*-phenothiazine-2-carbonitrile
Formula	$C_{13}H_8N_2S$
Fw	224.27
cryst syst	monoclinic
space group	P21/n
Size (mm^3)	0.30 x 0.20 x 0.20
T, K	296(2)
a, Å	7.856(3)
b, Å	5.845(2)
c, Å	22.870(8)
α, deg	90
β, deg	94.612(7)
γ, deg	90
V, A^3	1046.8(7)
Z	4

d_{calcd}g·cm^{-3}	1.423
μ, mm^{-1}	0.277
Refl collected	6486
T_{min}/T_{max}	0.420/0.746
N measd	2837
[R_{int}]	0.0543
Final *R* [I>2sigma(I)]	R_1 = 0.0819, wR_2 = 0.2271
R indices [all data]	R_1 = 0.1154, wR_2 = 0.2473
GOF	1.001

Single crystal of ^{13}C-**2ax** was obtained by recrystallization in CH_2Cl_2/EA. CCDC: 2003645 contains the supplementary crystallographic data which can be obtained free of charge from the Cambridge Crystallography Data Center via www.ccdc.cam.ac.uk/data_request/cif.

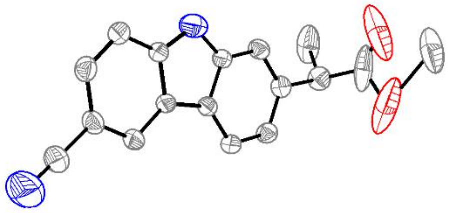

Supplementary Figure 6. Thermal Ellipsoid Depiction of Compound ^{13}C-**2ax**

Supplementary Table 4

Crystal data and structure refinement for compound ^{13}C-**2ax**

	methyl 2-(6-(cyano-^{13}C)-9*H*-carbazol-2-yl)propanoate
Formula	$C_{16}{}^{13}CH_{14}N_2O_2$
Fw	279.30
cryst syst	orthorhombic
space group	Pbca
Size (mm^3)	0.30 x 0.20 x 0.20

T, K	296(2)
a, Å	16.663(3)
b, Å	7.4730(12)
c, Å	22.987(3)
α, deg	90
β, deg	90
γ, deg	90
V, A^3	2862.4(8)
Z	8
d_{calcd}g·cm^{-3}	1.292
μ, mm^{-1}	0.086
Refl collected	13404
T_{min}/T_{max}	0.673/0.745
N measd	2496
[R_{int}]	0.0371
Final *R* [I>2sigma(I)]	R_1 = 0.0764, wR_2 = 0.2020
R indices [all data]	R_1 = 0.1067, wR_2 = 0.2294
GOF	1.008

Single crystal of [Ni(dppp)$_2$]BF_4 was obtained by recrystallization in CH_2Cl_2/heptane. CCDC: 1974241 contains the supplementary crystallographic data which can be obtained free of charge from the Cambridge Crystallography Data Center via www.ccdc.cam.ac.uk/data_request/cif.

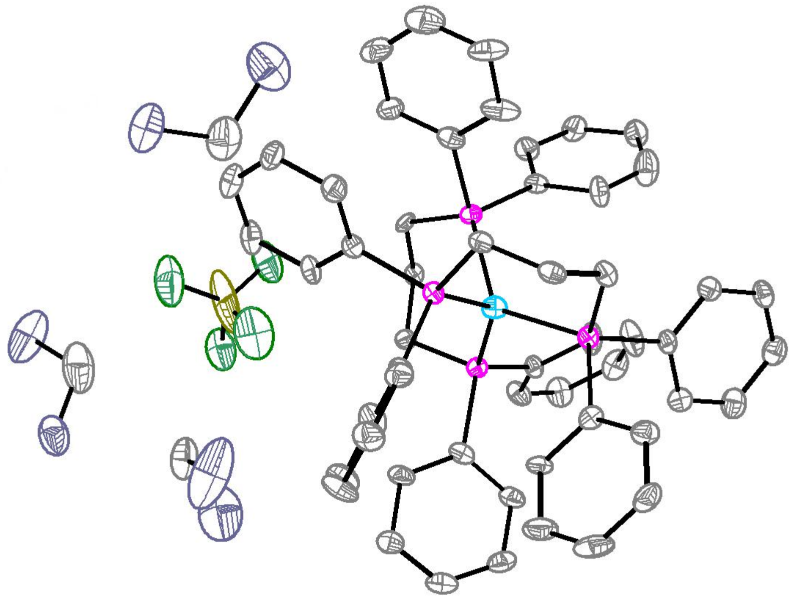

Supplementary Figure 7. Thermal Ellipsoid Depiction of Compound [Ni(dppp)$_2$]$BF_4(CH_2Cl_2)_3$

Supplementary Table 5

Crystal data and structure refinement for compound [Ni(dppp)$_2$]$BF_4(CH_2Cl_2)_3$

	[Ni(dppp)$_2$]$BF_4(CH_2Cl_2)_3$
Formula	$C_{57}H_{58}BF_4NiP_4Cl_6$
Fw	1225.13
cryst syst	tetragonal
space group	P43
Size (mm^3)	0.30 x 0.20 x 0.20
T, K	150(2)
a, Å	12.722(2)
b, Å	12.722(2)
c, Å	35.305(7)
α, deg	90
β, deg	90
γ, deg	90
V, A^3	5714(2)

Z	4
d_{calcd} g·cm^{-3}	1.424
μ, mm^{-1}	0.783
Refl collected	28635
T_{min}/T_{max}	0.582/0.745
N measd	9962
[R_{int}]	0.0590
Final R [I>2sigma(I)]	R_1 = 0.0873, wR_2 = 0.2449
R indices [all data]	R_1 = 0.1035, wR_2 = 0.2604
GOF	1.001

Characterization of products

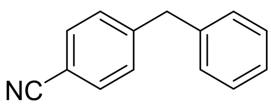

2e (**4-benzylbenzonitrile**), colorless oil; ^{1}H NMR (400 MHz, $CDCl_3$) δ: 7.55 (dt, J = 8.4, 1.6 Hz, 2H), 7.32-7.21 (m, 5H), 7.16-7.14 (m, 2H), 4.02 (s, 2H); ^{13}C NMR (100 MHz, $CDCl_3$) δ: 146.67, 139.27, 132.22, 129.57, 128.89, 128.70, 126.60, 118.90, 110.00, 41.90; HRMS (ESI): Calcd. for $C_{14}H_{11}NNa^+$ $[M + Na]^+$: 216.0784, Found: 216.0784.

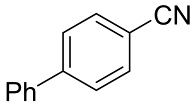

2g (**[1,1'-biphenyl]-4-carbonitrile**), white solid; ^{1}H NMR (400 MHz, $CDCl_3$) δ: 7.74-7.68 (m, 4H), 7.61-7.58 (m, 2H), 7.51-7.43 (m, 3H); ^{13}C NMR (100 MHz, $CDCl_3$) δ: 145.68, 139.18, 132.58, 129.10, 128.64, 127.72, 127.21, 118.90, 110.94.

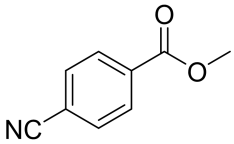

2k (**methyl 4-cyanobenzoate**), white solid; ^{1}H NMR (400 MHz, $CDCl_3$) δ: 8.14-8.11 (m, 2H), 7.75-7.72 (m, 2H), 3.95 (s, 3H); ^{13}C NMR (100 MHz, $CDCl_3$) δ: 165.38, 133.93, 132.18, 130.06, 117.89, 116.40, 52.65; HRMS (ESI): Calcd. for $C_9H_7NNaO_2^+$ $[M + Na]^+$: 184.0369, Found: 184.0369.

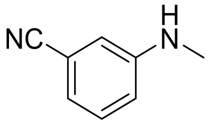

2m (**3-(methylamino)benzonitrile**), pale brown solid; ^{1}H NMR (400 MHz, $CDCl_3$) δ: 7.24-7.20 (m, 1H), 6.94 (dt, J = 7.6, 1.2 Hz, 1H), 6.78-6.77 (m, 2H), 4.00 (s, 1H), 2.83 (d, J = 4.0 Hz, 3H); ^{13}C NMR (100 MHz, $CDCl_3$) δ: 149.35, 129.73, 120.40, 119.52, 116.81, 114.28, 112.76, 30.20.

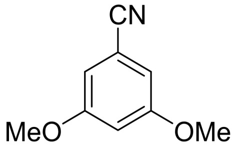

2q (**3,5-dimethoxybenzonitrile**), white solid; ^{1}H NMR (400 MHz, $CDCl_3$) δ: 6.753-6.746 (m, 2H), 6.64 (t, J = 2.0 Hz, 1H), 3.80 (s, 6H); ^{13}C NMR (100 MHz, $CDCl_3$) δ: 160.96, 118.67, 113.37, 109.85, 105.59, 55.60; HRMS (ESI): Calcd. for $C_9H_9NNaO_2^+$ $[M + Na]^+$: 186.0525, Found: 186.0525.

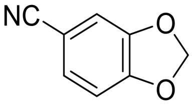

2s (benzo[*d*][1,3]dioxole-5-carbonitrile), white solid; ^{1}H NMR (400 MHz, $CDCl_3$) *δ*: 7.20 (dd, *J* = 8.0, 1.6 Hz, 1H), 7.02 (d, *J* = 1.6 Hz, 1H), 6.85 (d, *J* = 8.0 Hz, 1H), 6.06 (s, 2H); ^{13}C NMR (100 MHz, $CDCl_3$) *δ*: 151.49, 148.00, 128.16, 118.82, 111.36, 109.08, 104.94, 102.16.

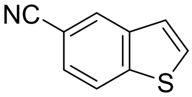

2t (**benzo[*b*]thiophene-5-carbonitrile**), white solid; ^{1}H NMR (400 MHz, DMSO-d_6) *δ*: 8.41 (s, 1H), 8.23 (d, *J* = 8.4 Hz, 1H), 7.98 (d, *J* = 4.8 Hz, 1H), 7.70 (d, *J* = 8.4 Hz, 1H), 7.56 (d, *J* = 5.2 Hz, 1H); ^{13}C NMR (100 MHz, DMSO-d_6) *δ*: 143.66, 139.41, 130.62, 128.46, 126.15, 124.18, 124.02, 119.50, 107.03; HRMS (ESI): Calcd. for $C_9H_5NNaS^+$ $[M + Na]^+$: 182.0035, Found: 182.0029.

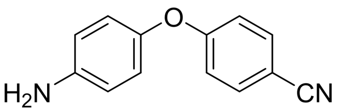

2w (**4-(4-aminophenoxy)benzonitrile**), pale yellow solid; ^{1}H NMR (400 MHz, $CDCl_3$) *δ*: 7.57-7.54 (m, 2H), 6.96-6.92 (m, 2H), 6.89-6.85 (m, 2H), 6.73-6.69 (m, 2H), 3.69 (s, 2H); ^{13}C NMR (100 MHz, $CDCl_3$) *δ*: 162.81, 146.35, 143.97, 133.99, 121.81, 119.02, 116.94, 116.28, 104.91; HRMS (ESI): Calcd. for $C_{13}H_{10}N_2NaO^+$ $[M + Na]^+$: 233.0685, Found: 233.0690.

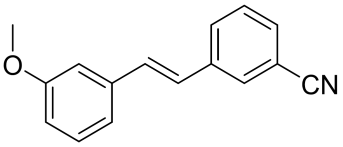

2x (**3-(3-methoxystyryl)benzonitrile**), colorless oil; ^{1}H NMR (400 MHz, $CDCl_3$) *δ*: 7.77 (s, 1H), 7.70 (d, *J* = 7.6 Hz, 1H), 7.54-7.51 (m, 1H), 7.45 (t, *J* = 7.6 Hz, 1H), 7.31 (t, *J* = 8.0 Hz, 1H), 7.15-7.02 (m, 4H), 6.89-6.86 (m, 1H), 3.86 (s, 3H); ^{13}C NMR (100 MHz, $CDCl_3$) *δ*: 159.95, 138.50, 137.77, 131.21, 130.69, 130.51, 129.85, 129.78, 129.43, 126.47, 119.44, 118.71, 114.04, 112.93, 112.06, 55.26; HRMS (ESI): Calcd. for $C_{16}H_{13}NNaO^+$ $[M + Na]^+$: 258.0889, Found: 258.0893.

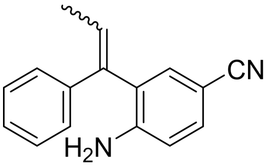

2y (**4-amino-3-(1-phenylprop-1-en-1-yl)benzonitrile**), white solid; ^{1}H NMR (400 MHz, DMSO-d_6): too complex to analyze due to the cis-trans isomerism; ^{13}C NMR (100 MHz, DMSO-d_6) *δ*: 150.27, 140.09, 136.45, 134.21, 132.47, 128.44, 127.09, 126.82, 125.98, 123.34, 120.52, 114.37, 96.28, 15.38; HRMS (ESI): Calcd. for $C_{16}H_{14}N_2Na^+$ $[M + Na]^+$: 257.1049, Found: 257.1049.

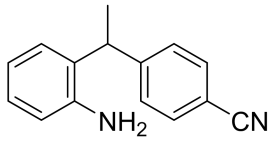

2z (**4-(1-(2-aminophenyl)ethyl)benzonitrile**), yellow solid; ^{1}H NMR (400 MHz, $CDCl_3$) *δ*: 7.59-7.56 (m, 2H), 7.32-7.30 (m, 2H), 7.24-7.22 (m, 1H), 7.14-7.10 (m, 1H), 6.88-6.84 (m, 1H), 6.69-6.66 (m, 1H), 4.14 (q, *J* = 7.2 Hz, 1H), 3.41 (s, 2H), 1.63 (d, *J* = 7.2 Hz, 3H); ^{13}C NMR (100 MHz, $CDCl_3$) *δ*: 151.35, 143.94, 134.14, 132.54, 128.25, 127.81, 127.24, 119.06, 118.85, 116.47, 110.27, 40.11, 21.37; HRMS (ESI): Calcd. for $C_{15}H_{14}N_2Na^+$ [M + Na]$^+$: 245.1049, Found: 245.1048.

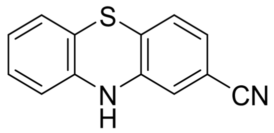

2aa (**10*H*-phenothiazine-2-carbonitrile**), yellow solid; ^{1}H NMR (400 MHz, DMSO-*d*$_6$) *δ*: 8.87 (s, 1H), 7.11 (dd, *J* = 8.4, 1.6 Hz, 1H), 7.05-6.98 (m, 2H), 6.90-6.86 (m, 2H), 6.77 (td, *J* = 7.6, 0.8 Hz, 1H), 6.64 (d, *J* = 7.6 Hz, 1H); ^{13}C NMR (100 MHz, DMSO-*d*$_6$) *δ*: 142.67, 140.66, 128.24, 127.11, 126.44, 125.28, 123.99, 122.70, 118.75, 116.07, 115.18, 114.86, 109.80; HRMS (ESI): calcd for $C_{13}H_8N_2NaS^+$ [M+Na]$^+$: 247.0300, found: 247.0300.

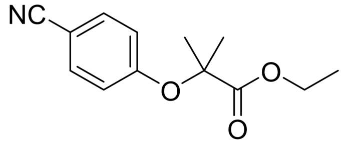

2ab (**ethyl 2-(4-cyanophenoxy)-2-methylpropanoate**), white solid; ^{1}H NMR (400 MHz, $CDCl_3$) *δ*: 7.56-7.52 (m, 2H), 6.87-6.83 (m, 2H), 4.22 (q, *J* = 7.2 Hz, 2H), 1.65 (s, 6H), 1.22 (t, *J* = 7.2 Hz, 3H); ^{13}C NMR (100 MHz, $CDCl_3$) *δ*: 173.26, 159.21, 133.57, 118.93, 118.27, 104.71, 79.63, 61.74, 25.28, 13.95; HRMS (ESI): Calcd. for $C_{13}H_{15}NNaO_3^+$ [M + Na]$^+$: 256.0944, Found: 256.0945.

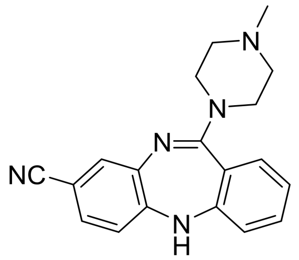

2ac (**11-(4-methylpiperazin-1-yl)-5*H*-dibenzo[*b,e*][1,4]diazepine-8-carbonitrile**), yellow solid; ^{1}H NMR (400 MHz, $CDCl_3$) *δ*: 7.34-7.28 (m, 3H), 7.12 (dd, *J* = 8.0, 2.0 Hz, 1H), 7.05 (td, *J* = 7.6, 0.8 Hz, 1H), 6.82 (dd, *J* = 8.0, 0.8 Hz, 1H), 6.71 (d, *J* = 8.0 Hz, 1H), 5.10 (s, 1H), 3.49 (s, 4H), 2.50 (s, 4H), 2.35 (s, 3H); ^{13}C NMR (100 MHz, $CDCl_3$) *δ*: 162.93, 151.47, 146.29, 141.18, 132.14, 130.90, 130.38, 127.12, 123.49, 123.43, 120.38, 119.78, 119.22, 107.43, 54.96, 47.23, 46.09; HRMS (ESI): Calcd. for $C_{19}H_{20}N_5^+$ [M + H]$^+$: 318.1713, Found: 318.1715.

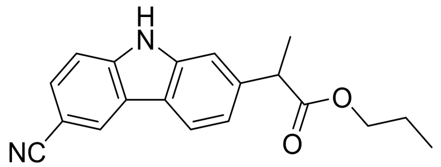

2ad (**propyl 2-(6-cyano-9*H*-carbazol-2-yl)propanoate**), white solid; ^{1}H NMR (400 MHz, $CDCl_3$) δ: 9.13 (s, 1H), 8.29-8.28 (m, 1H), 7.95 (d, J = 8.0 Hz, 1H), 7.60 (dd, J = 8.4, 1.6 Hz, 1H), 7.44-7.42 (m, 2H), 7.24 (dd, J = 8.0, 1.6 Hz, 1H), 4.12-4.02 (m, 2H), 3.91 (dd, J = 14.4, 7.2 Hz, 1H), 1.66-1.57 (m, 5H), 0.86 (t, J = 7.2 Hz, 3H); ^{13}C NMR (100 MHz, $CDCl_3$) δ: 174.94, 141.74, 140.30, 140.03, 128.75, 125.06, 123.10, 121.18, 120.61, 120.56, 120.45, 111.35, 109.85, 101.81, 66.54, 45.94, 21.86, 18.76, 10.23; HRMS (ESI): calcd for $C_{19}H_{19}N_2O_2^+$ [M+H]$^+$: 307.1441, found: 307.1441.

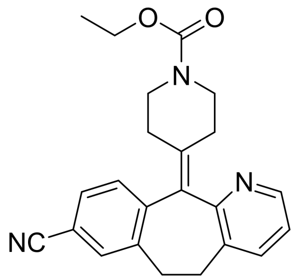

2ae (**ethyl 4-(8-cyano-5,6-dihydro-11*H*-benzo[5,6]cyclohepta[1,2-*b*]pyridin-11-ylidene)piperidine-1-carboxylate**), white solid; ^{1}H NMR (400 MHz, $CDCl_3$) δ: 8.43-8.42 (m, 1H), 7.49-7.46 (m, 3H), 7.30 (d, J = 8 Hz, 1H), 7.15-7.12 (m, 1H), 4.17-4.12 (m, 2H), 3.85-3.79 (m, 2H), 3.50-3.35 (m, 2H), 3.21-3.14 (m, 2H), 2.91-2.84 (m, 2H), 2.55-2.48 (m, 1H), 2.40-2.25 (m, 3H), 1.28-1.24 (m, 3H); ^{13}C NMR (100 MHz, $CDCl_3$) δ: 155.95, 155.38, 146.88, 144.33, 139.16, 138.58, 137.67, 133.89, 133.06, 132.54, 129.89, 129.74, 122.51, 118.69, 111.11, 61.29, 44.64, 31.38, 31.32, 30.74, 30.45, 14.58; HRMS (ESI): Calcd. for $C_{23}H_{24}N_3O_2^+$ [M + H]$^+$: 374.1863, Found: 374.1866.

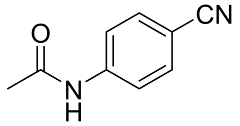

2ah [1,1'-biphenyl]-4-carbonitrile, white solid; ^{1}H NMR (400 MHz, DMSO-d_6) δ: 10.35 (s, 1H),7.77-7.72 (m, 4H), 2.09 (s, 3H); ^{13}C NMR (100 MHz, DMSO-d_6) δ: 169.21, 143.52, 133.27, 119.13, 118.98, 104.76, 24.24.

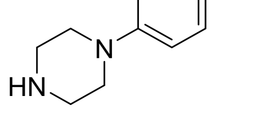

2ai (**4-(piperazin-1-yl)benzonitrile**), pale yellow solid; ^{1}H NMR (400 MHz, $CDCl_3$) δ: 7.46 (dd, J = 8.8, 1.6 Hz, 2H), 6.83 (dd, J = 8.8, 1.6 Hz, 2H), 3.27-3.25 (m, 4H), 2.99-2.97 (m, 4H); ^{13}C NMR (100 MHz, $CDCl_3$) δ: 153.71, 133.38, 119.97, 114.10, 100.10, 48.14, 45.61; HRMS (ESI): Calcd. for $C_{11}H_{14}N_3^+$ [M + H]$^+$: 188.1182, Found: 188.1182.

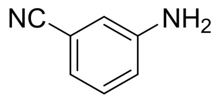

2ak (**3-aminobenzonitrile**), pale brown oil; ^{1}H NMR (400 MHz, DMSO-d_6) δ: 7.20-7.16 (m, 1H), 6.86-6.84 (m, 3H), 5.56 (s, 2H); ^{13}C NMR (100 MHz, DMSO-d_6) δ: 149.60, 130.27, 119.67, 119.00, 118.63, 115.96, 111.64.

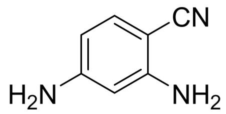

2al (**2,4-diaminobenzonitrile**), pale brown solid; ^{1}H NMR (400 MHz, DMSO-d_6) δ: 6.99-6.97 (m, 1H), 5.87-5.85 (m, 2H), 5.69 (s, 2H), 5.46 (s, 2H); ^{13}C NMR (100 MHz, DMSO-d_6) δ: 153.64, 152.62, 133.24, 119.95, 104.67, 97.25, 81.49.

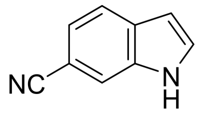

2an (**1*H*-indole-6-carbonitrile**), white solid; ^{1}H NMR (400 MHz, DMSO-d_6) δ: 11.67 (s, 1H), 7.91-7.90 (m, 1H), 7.71 (dd, J = 8.0, 0.8 Hz, 1H), 7.65 (d, J = 2.8 Hz, 1H), 7.32 (dd, J = 8.4, 1.6 Hz, 1H), 6.59 (d, J = 2.8 Hz, 1H); ^{13}C NMR (100 MHz, DMSO-d_6) δ: 134.74, 130.99, 129.95, 121.57, 121.24, 120.79, 116.45, 102.38, 102.12; HRMS (ESI): Calcd. for $C_9H_6N_2Na^+$ $[M + Na]^+$: 165.0423, Found: 165.0426.

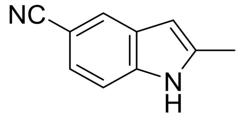

2ap (**2-methyl-1*H*-indole-5-carbonitrile**), white solid; ^{1}H NMR (400 MHz, DMSO-d_6) δ: 11.53 (s, 1H), 7.91 (s, 1H), 7.41 (d, J = 8.4 Hz, 1H), 7.34-7.32 (m, 1H), 6.27 (s, 1H), 2.41 (s, 3H); ^{13}C NMR (100 MHz, DMSO-d_6); δ: 138.76, 137.97, 128.50, 124.29, 123.04, 121.04, 111.69, 100.67, 100.04, 13.39; HRMS (ESI): calcd for $C_{10}H_8N_2Na^+$ $[M+Na]^+$: 179.0580, found: 179.0578.

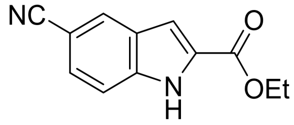

2aq (**ethyl 5-cyano-1*H*-indole-2-carboxylate**), white solid; ^{1}H NMR (400 MHz, DMSO-d_6) δ: 12.42 (s, 1H), 8.23 (s, 1H), 7.61-7.56 (m, 2H), 7.26 (d, J = 1.6 Hz, 1H), 4.36 (q, J = 7.2 Hz, 2H), 1.34 (t, J = 7.2 Hz, 3H); ^{13}C NMR (100 MHz, DMSO-d_6) δ: 160.79, 138.75, 129.88, 128.33, 126.65, 126.44, 120.11, 113.97, 108.35, 102.55, 60.94, 14.23; HRMS (ESI): Calcd. for $C_{12}H_{10}N_2NaO_2^+$ $[M + Na]^+$: 237.0634, Found: 237.0636.

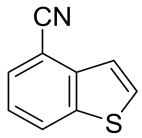

2ar (**benzo[*b*]thiophene-4-carbonitrile**), white solid; ^{1}H NMR (400 MHz, DMSO-d_6) δ: 8.40-8.37 (m, 1H), 8.13-8.10 (m, 1H), 7.92-7.89 (m, 1H), 7.58-7.49 (m, 2H); ^{13}C NMR (100 MHz, DMSO-d_6) δ: 140.01, 139.76, 132.50, 129.85, 128.17, 124.26, 121.52, 117.83, 105.46; HRMS (ESI): Calcd. for $C_9H_5NNaS^+$ [M + Na]$^+$: 182.0035, Found: 182.0035.

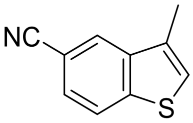

2as (**3-methylbenzo[*b*]thiophene-5-carbonitrile**), white solid; ^{1}H NMR (400 MHz, CDCl$_3$) δ: 8.01 (s, 1H), 7.91 (d, J = 8.4 Hz, 1H), 7.54 (dd, J = 8.4, 0.8 Hz, 1H), 7.22 (s, 1H), 2.46 (s, 3H); ^{13}C NMR (100 MHz, CDCl$_3$) δ: 144.44, 139.52, 132.17, 126.33, 126.00, 124.11, 123.63, 119.54, 107.50, 13.65; HRMS (ESI): calcd for $C_{10}H_7NS^+$ [M]$^+$: 173.0294, found: 173.0291.

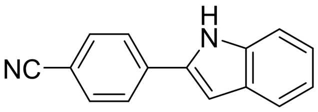

2at (**4-(1*H*-indol-2-yl)benzonitrile**), white solid; ^{1}H NMR (400 MHz, DMSO-d_6) δ: 11.77 (s, 1H), 8.03 (d, J = 8.0 Hz, 2H), 7.89 (d, J = 8.0 Hz, 2H), 7.57 (d, J = 8.0 Hz, 1H), 7.57 (d, J = 8.0 Hz, 1H), 7.44 (d, J = 8.0 Hz, 1H), 7.17 (t, J = 7.6 Hz, 1H), 7.12 (s, 1H), 7.04 (t, J = 7.6 Hz, 1H); ^{13}C NMR (100 MHz, DMSO-d_6) δ: 137.79, 136.63, 135.68, 132.96, 128.47, 125.44, 122.87, 120.78, 119.93, 119.13, 111.73, 109.27, 101.66; HRMS (ESI): Calcd. for $C_{15}H_{10}N_2Na^+$ [M + Na]$^+$: 241.0736, Found: 241.0736.

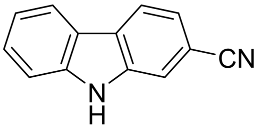

2au (**9*H*-carbazole-2-carbonitrile**), pale brown solid; ^{1}H NMR (400 MHz, DMSO-d_6) δ: 11.69 (br s, 1H), 8.32-8.30 (m, 1H), 8.24-8.22 (m, 1H), 7.983-7.978 (m, 1H), 7.60-7.48 (m, 3H), 7.26-7.22 (m, 1H); ^{13}C NMR (100 MHz, DMSO-d_6) δ: 141.08, 138.62, 127.60, 126.01, 121.57, 121.41, 121.32 (2C), 120.18, 119.56, 115.27, 111.65, 106.88; HRMS (ESI): Calcd. for $C_{13}H_8N_2K^+$ [M + K]$^+$: 231.0319, Found: 231.0316.

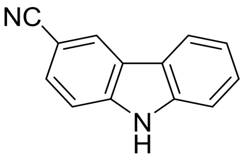

2av (**9*H*-carbazole-3-carbonitrile**), white solid; ^{1}H NMR (400 MHz, DMSO-d_6) δ: 11.86 (s, 1H), 8.69 (s, 1H), 8.23 (d, J = 8.0 Hz, 1H), 7.74 (d, J = 8.4 Hz, 1H), 7.63 (d, J = 8.4 Hz, 1H), 7.57 (d, J = 8.4 Hz, 1H), 7.49 (t, J = 7.6 Hz, 1H), 7.26 (t, J = 7.6 Hz, 1H); ^{13}C NMR (100 MHz, DMSO-d_6) δ: 141.76, 140.35, 128.65, 127.05, 125.64, 122.72, 121.67, 121.00, 120.66, 119.93, 112.09, 111.65, 100.30; HRMS (ESI): Calcd. for $C_{13}H_9N_2$ [M+H]$^+$: 193.0760, Found: 193.0759.

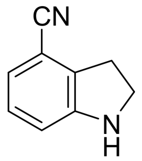

2aw (**indoline-4-carbonitrile**), pale brown solid; ^{1}H NMR (400 MHz, $CDCl_3$) δ: 7.05 (t, J = 8.0 Hz, 1H), 6.90 (d, J = 7.6 Hz, 1H), 6.75 (d, J = 7.6 Hz, 1H), 3.64 (t, J = 8.8 Hz, 2H), 3.20 (t, J = 8.8 Hz, 2H); ^{13}C NMR (100 MHz, $CDCl_3$) δ: 152.18, 133.55, 128.16, 121.03, 117.97, 112.61, 108.61, 46.85, 29.24.

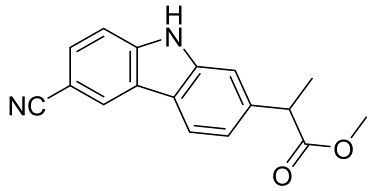

2ax (**methyl 2-(6-cyano-9*H*-carbazol-2-yl)propanoate**), white solid; ^{1}H NMR (400 MHz, $CDCl_3$) δ: 8.92 (br s, 1H), 8.27 (s, 1H), 7.95 (d, J = 8.4 Hz, 1H), 7.60 (dd, J = 8.8, 1.6 Hz, 1H), 7.43-7.41(m, 2H), 7.24 (dd, J = 8.0, 1.2 Hz, 1H), 3.93 (q, J = 7.2 Hz, 1H), 3.72 (s, 3H), 1.61 (d, J = 7.2 Hz, 3H); ^{13}C NMR (100 MHz, $CDCl_3$) δ: 175.36, 141.72, 140.27, 139.87, 128.81, 125.05, 123.09, 121.26, 120.69, 120.51, 120.44, 111.35, 109.87, 101.90, 52.18, 45.76, 18.80; HRMS (ESI): Calcd. for $C_{17}H_{14}N_2NaO_2^+$ $[M + Na]^+$: 301.0947, Found: 301.0941.

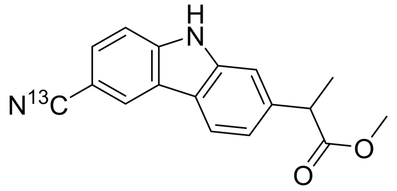

^{13}C-**2ax** (**methyl 2-(6-(cyano-^{13}C)-9*H*-carbazol-2-yl)propanoate**), white solid; ^{1}H NMR (400 MHz, $CDCl_3$) δ: 8.93 (br s, 1H), 8.28-8.27 (m, 1H), 7.95 (d, J = 8.0 Hz, 1H), 7.63-7.59 (m, 1H), 7.44-7.42 (m, 2H), 7.24 (d, J = 8.0 Hz, 1H), 3.93 (q, J = 7.2 Hz, 1H), 3.72 (s, 3H), 1.61 (d, J = 7.2 Hz, 3H); ^{13}C NMR (100 MHz, $CDCl_3$) not analyzed due to complexity; HRMS (ESI): Calcd. for $C_{16}{}^{13}CH_{15}N_2O_2^+$ $[M + H]^+$: 280.1162, Found: 280.1164.

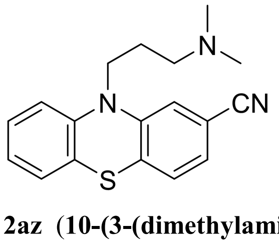

2az (**10-(3-(dimethylamino)propyl)-10*H*-phenothiazine-2-carbonitrile**), olive solid; ^{1}H NMR (400 MHz, DMSO-d_6) δ: 7.424-7.421 (m, 1H), 7.35-7.28 (m, 2H), 7.24-7.20 (m, 1H), 7.14 (dd, J = 7.6, 1.6 Hz, 1H), 7.04 (d, J = 7.6 Hz, 1H), 6.97 (td, J = 7.2, 1.2 Hz, 1H), 3.91 (t, J = 6.8 Hz, 2H), 2.28 (t, J = 6.8 Hz, 2H), 2.08 (s, 6H), 1.78-1.71 (m, 2H); ^{13}C NMR (100 MHz, DMSO-d_6) δ: 145.32, 143.64, 130.78, 128.24, 127.89, 127.36, 126.13, 123.32, 122.23, 118.94, 118.35, 116.42, 110.23, 56.21, 45.38, 44.74, 24.22; HRMS (ESI): Calcd. for $C_{18}H_{20}N_3S^+$ $[M + H]^+$: 310.1372, Found: 310.1372.

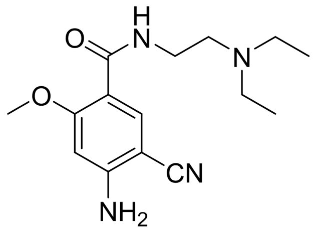

2ba (**4-amino-5-cyano-*N*-(2-(diethylamino)ethyl)-2-methoxybenzamide**), white solid; ^{1}H NMR (400 MHz, $CDCl_3$) δ: 8.30 (s, 1H), 8.13 (s, 1H), 6.22 (s, 1H), 4.77 (s, 2H), 3.93 (s, 3H), 3.51-3.47 (m, 2H), 2.64-2.55 (m, 6H), 1.04 (t, J = 7.2 Hz, 6H); ^{13}C NMR (100 MHz, $CDCl_3$) δ: 163.51, 161.95, 153.10, 137.86, 117.09, 113.42, 96.61, 89.34, 55.91, 51.49, 46.74, 37.43, 11.93; HRMS (ESI): Calcd. for $C_{15}H_{23}N_4O_2^+$ [M + H]$^+$: 291.1816, Found: 291.1819.

NMR spectra of products

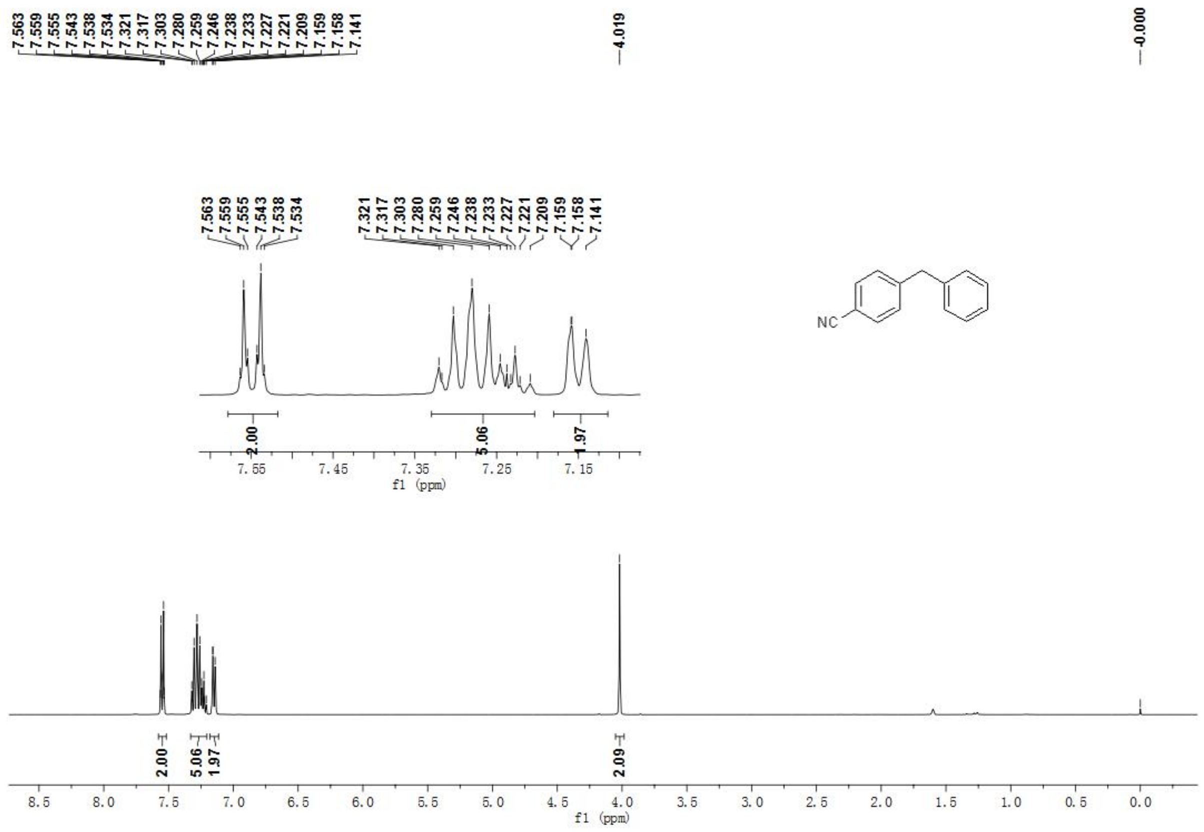

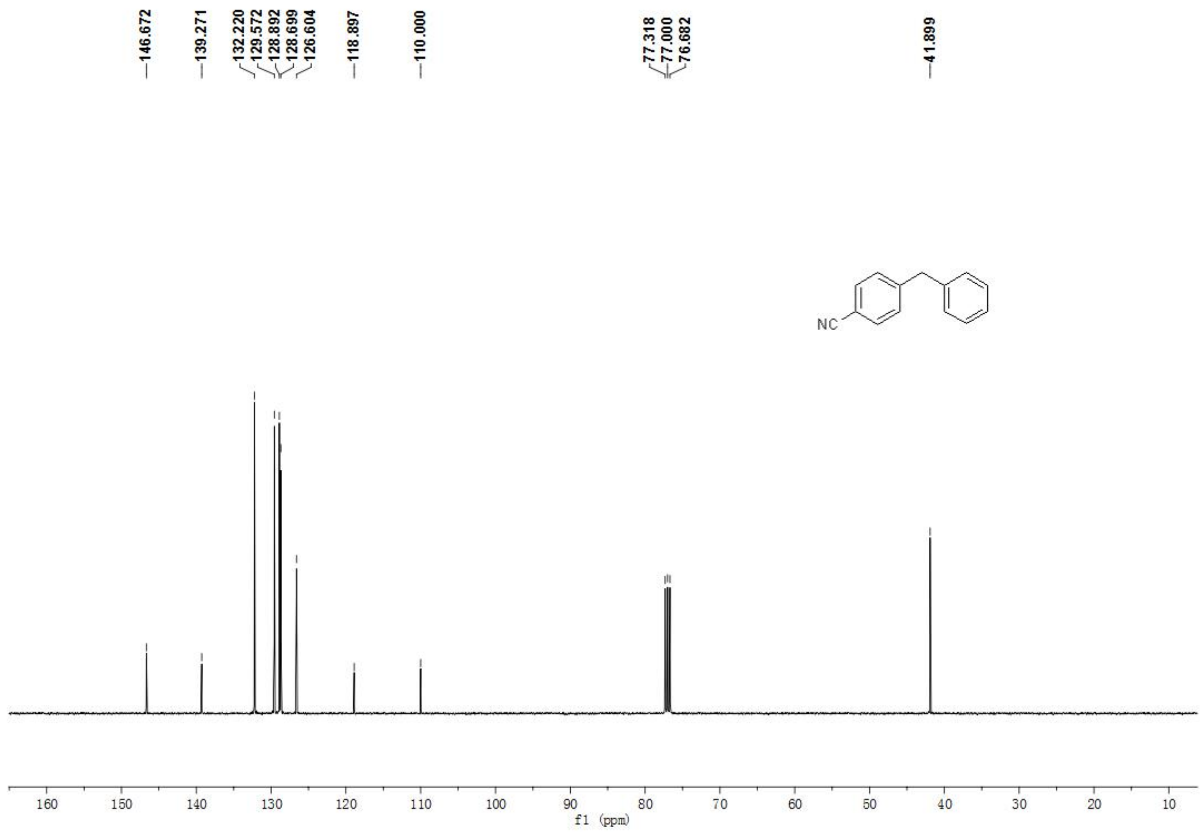

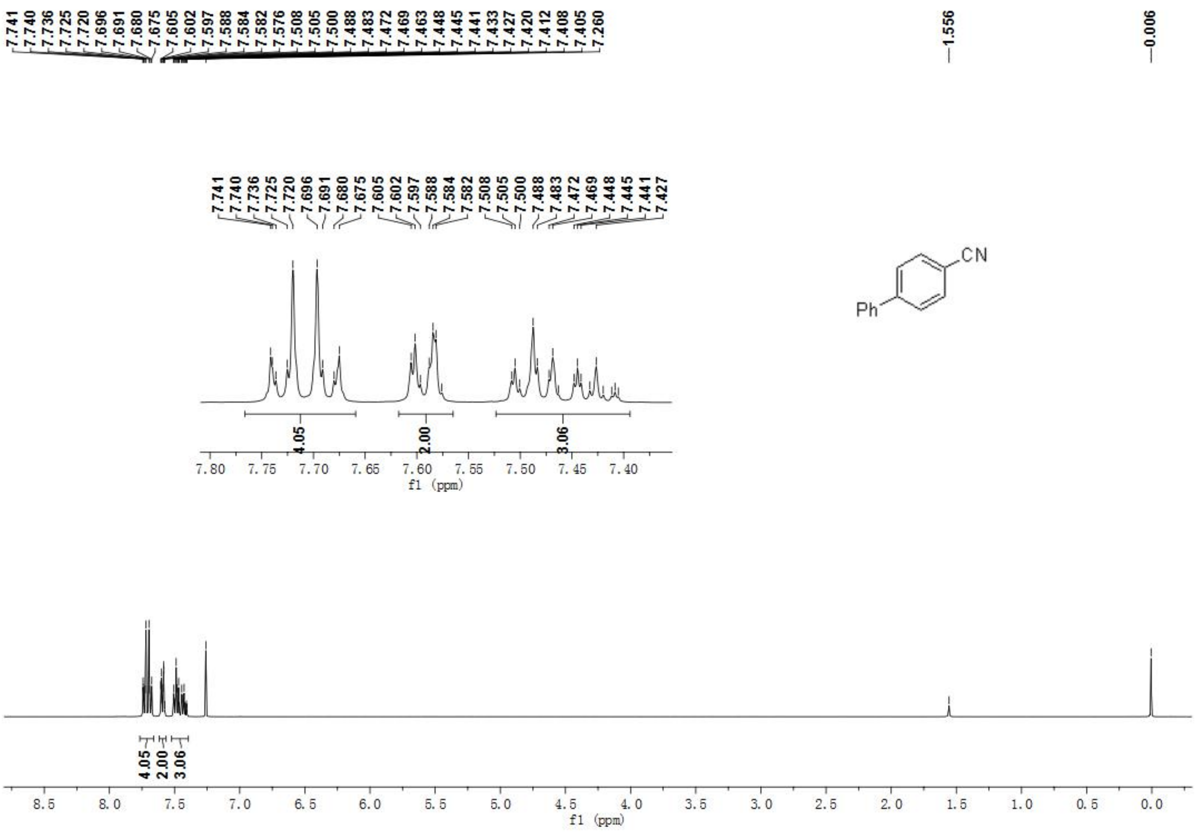

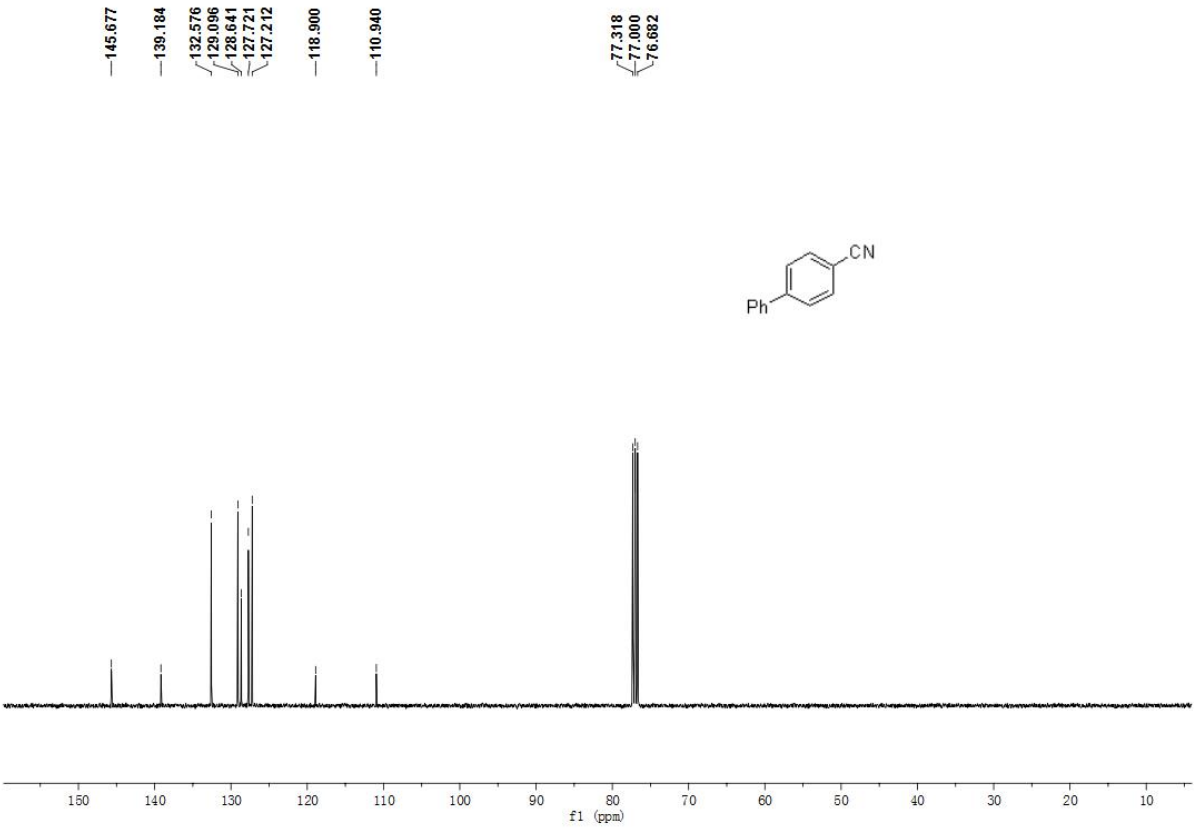

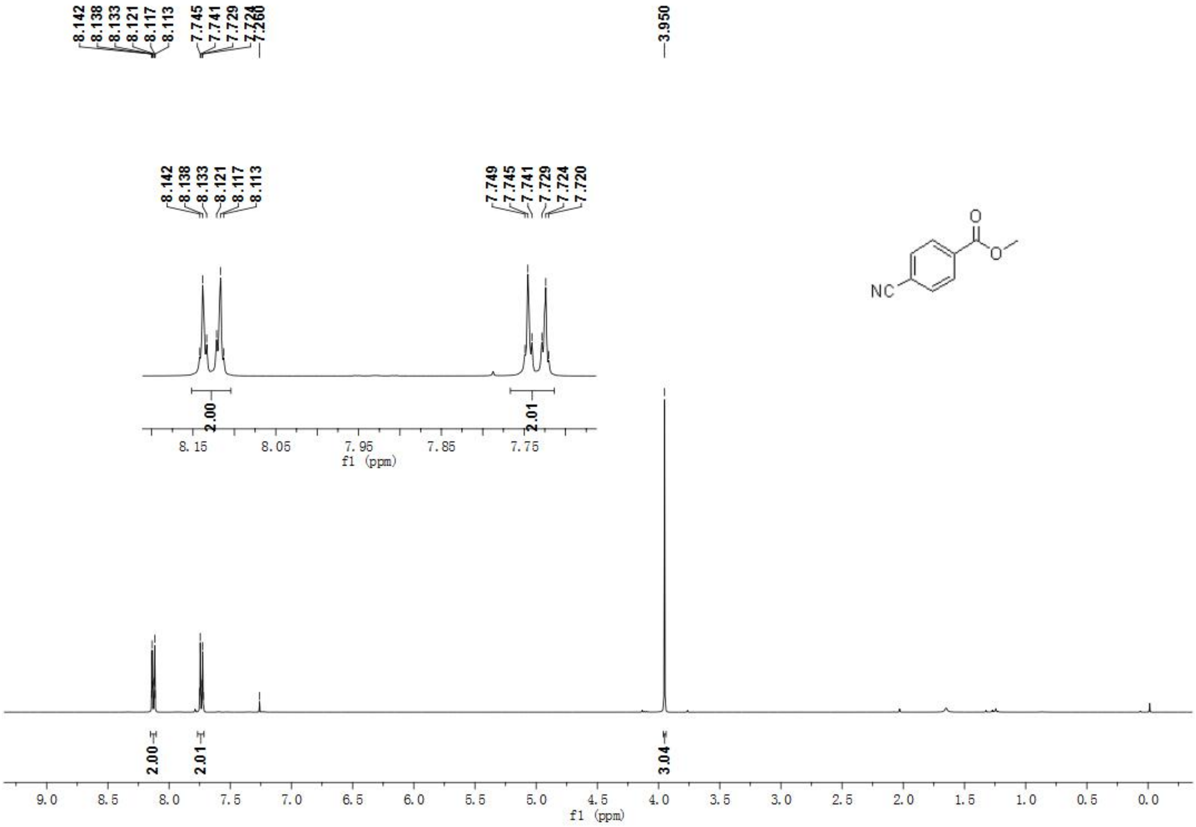
8.142
8.138
8.133
8.121
8.117
8.113
7.745
7.741
7.729
7.724
7.260
3.950
7.749
7.745
7.741
7.729
7.724
7.720
NC
O
O
2.00
2.01
8.15
8.05
7.95
7.85
7.75
f1 (ppm)
3.04
9.0
8.5
8.0
7.5
7.0
6.5
6.0
5.5
5.0
4.5
4.0
3.5
3.0
2.5
2.0
1.5
1.0
0.5
0.0
f1 (ppm)

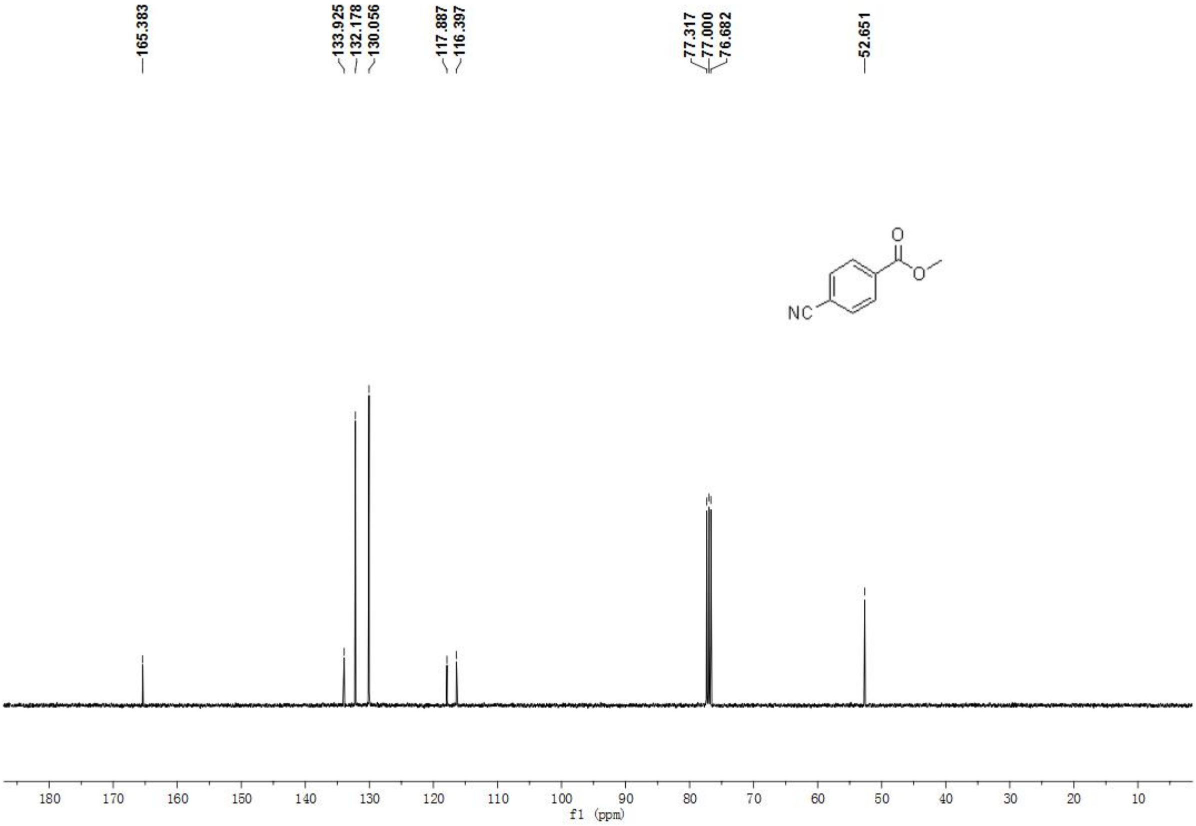
165.383
133.925
132.178
130.056
117.887
116.397
77.317
77.000
76.682
52.651
NC
O
O
180
170
160
150
140
130
120
110
100
90
80
70
60
50
40
30
20
10
f1 (ppm)

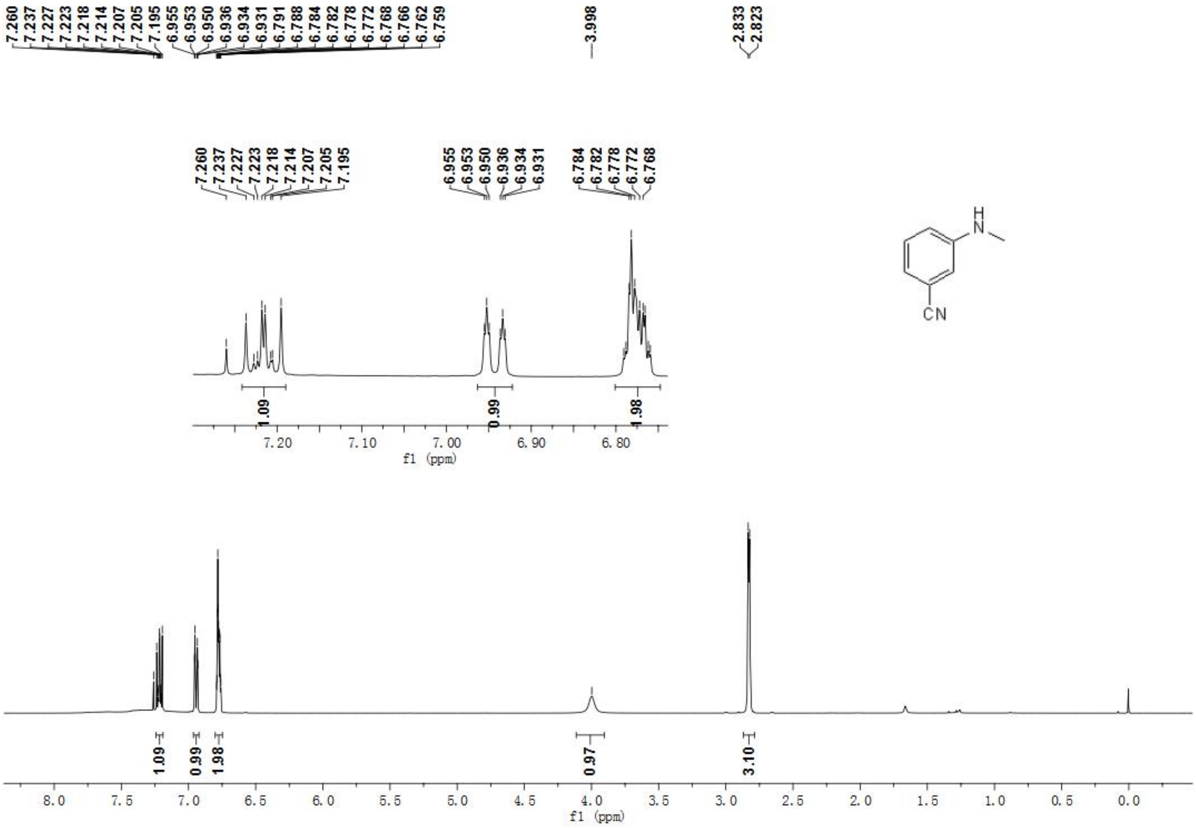
7.260
7.237
7.227
7.223
7.218
7.214
7.207
7.205
7.195
6.955
6.953
6.950
6.936
6.934
6.931
6.791
6.788
6.784
6.782
6.778
6.772
6.768
6.766
6.762
6.759
3.998
2.833
2.823
H
N
CN
1.09
0.99
1.98
0.97
3.10
f1 (ppm)

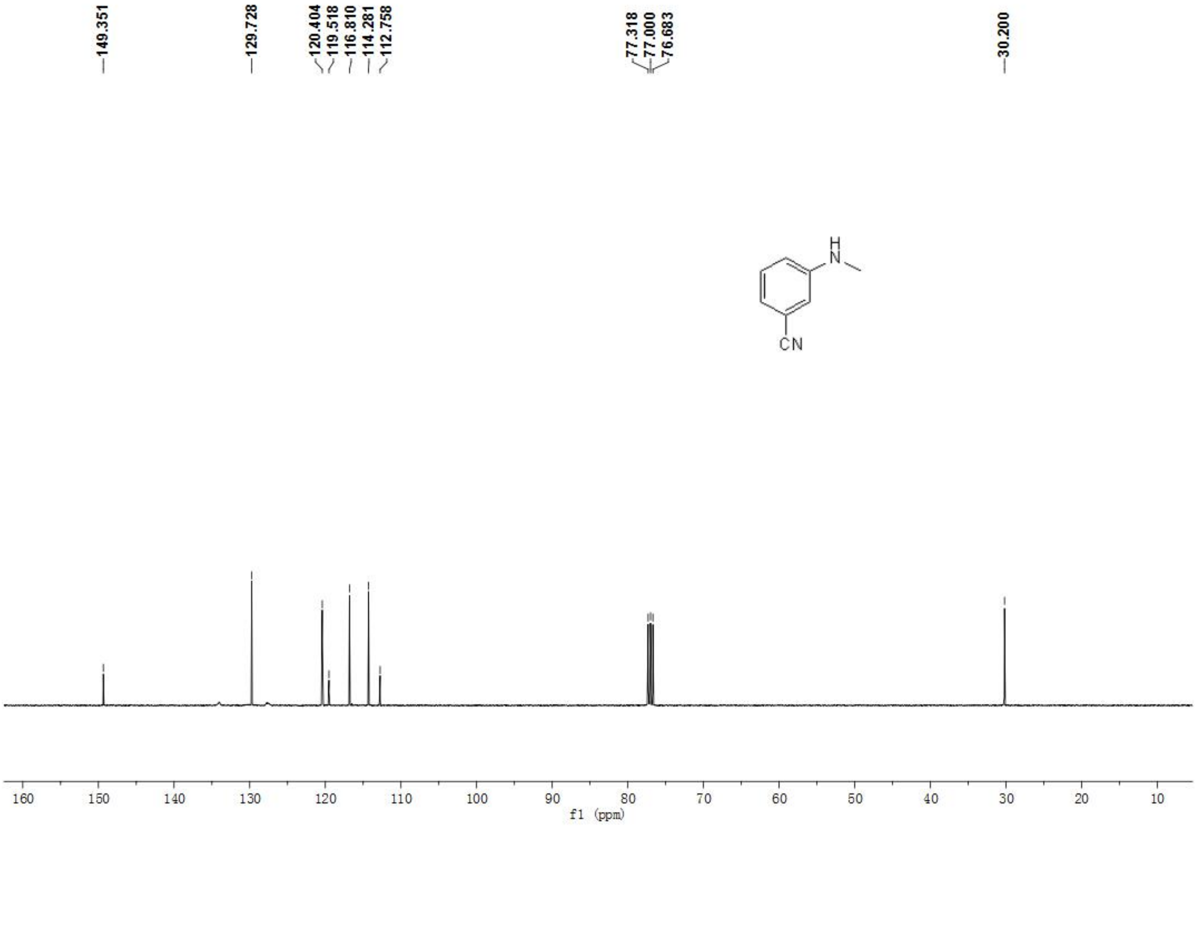
149.351
129.728
120.404
119.518
116.810
114.281
112.758
77.318
77.000
76.683
30.200
H
N
CN
f1 (ppm)

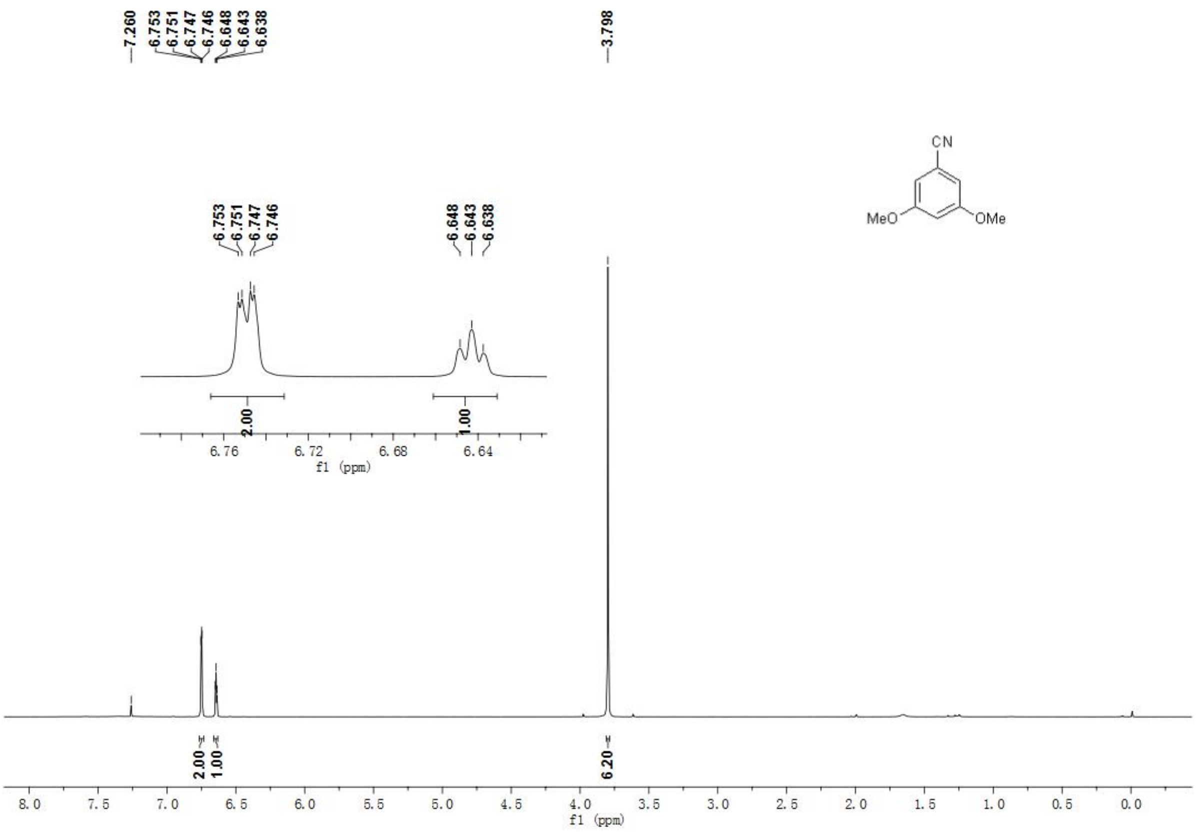
7.260
6.753
6.751
6.747
6.746
6.648
6.643
6.638
3.798
CN
MeO
OMe
2.00
1.00
6.20
f1 (ppm)

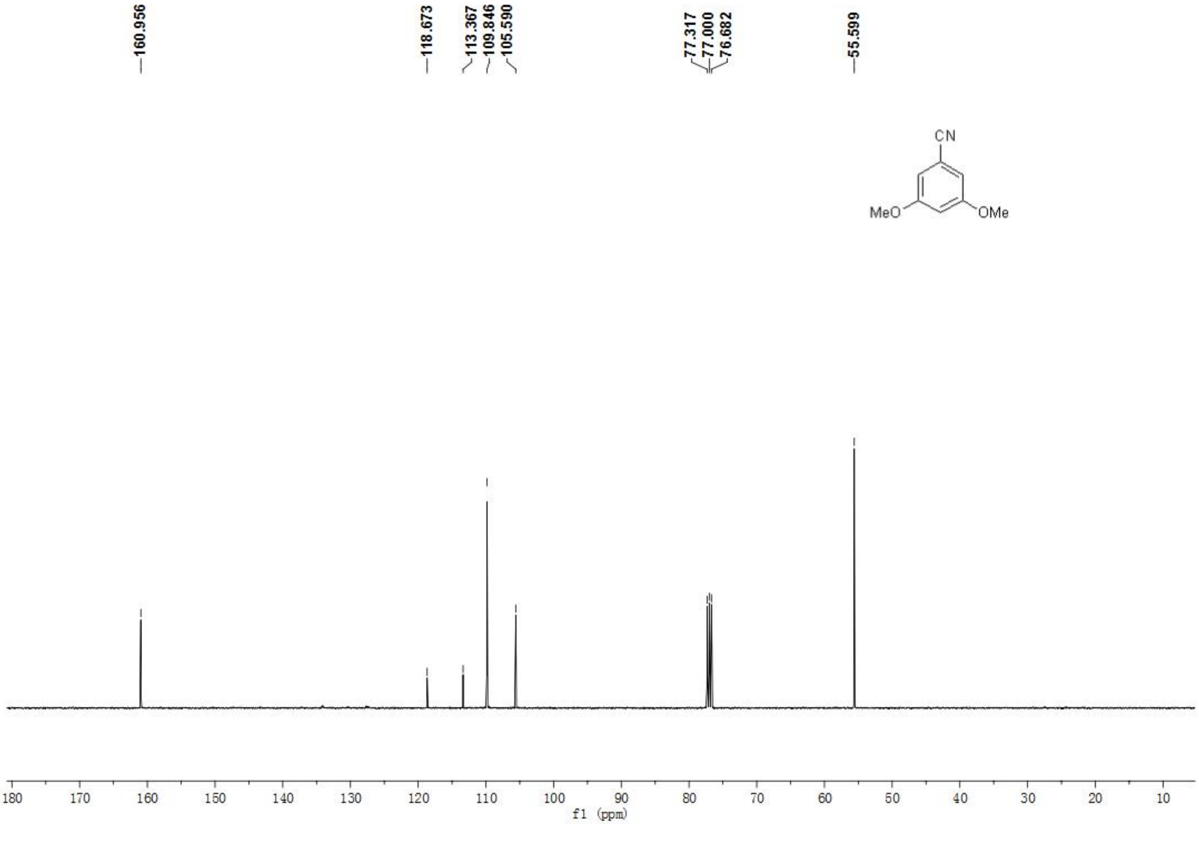
160.956
118.673
113.367
109.846
105.590
77.317
77.000
76.682
55.599
CN
MeO
OMe
f1 (ppm)

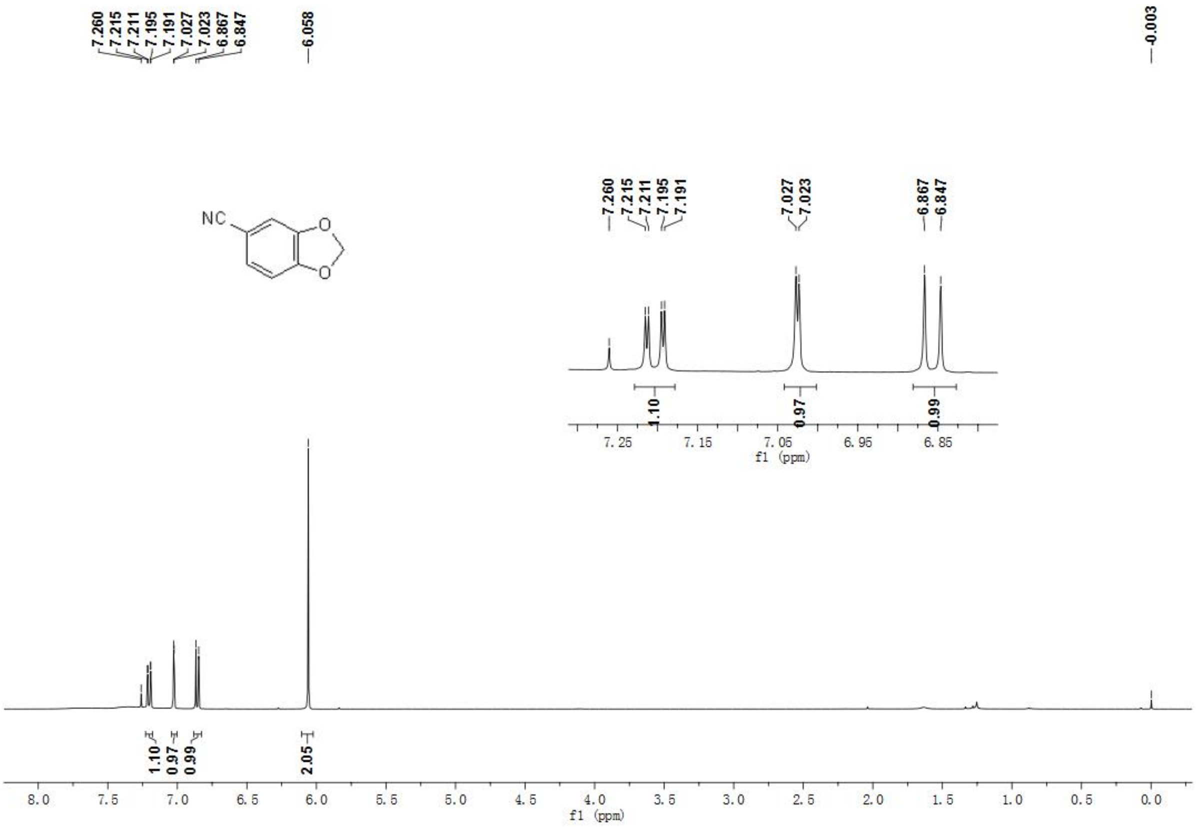

7.260
7.215
7.211
7.195
7.191
7.027
7.023
6.867
6.847
6.058
-0.003
NC
O
O
1.10
0.97
0.99
7.25
7.15
7.05
6.95
6.85
f1 (ppm)
2.05
8.0
7.5
7.0
6.5
6.0
5.5
5.0
4.5
4.0
3.5
3.0
2.5
2.0
1.5
1.0
0.5
0.0
f1 (ppm)

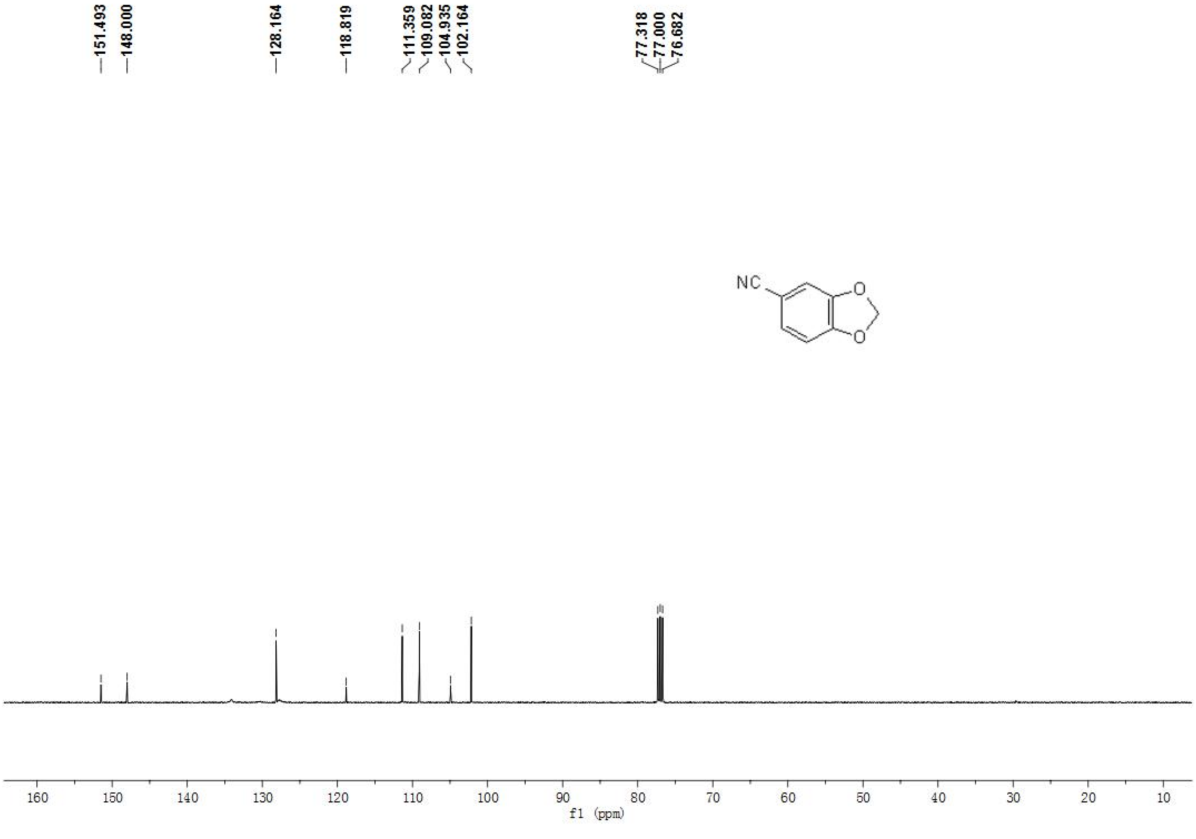

151.493
148.000
128.164
118.819
111.359
109.082
104.935
102.164
77.318
77.000
76.682
NC
O
O
160
150
140
130
120
110
100
90
80
70
60
50
40
30
20
10
f1 (ppm)

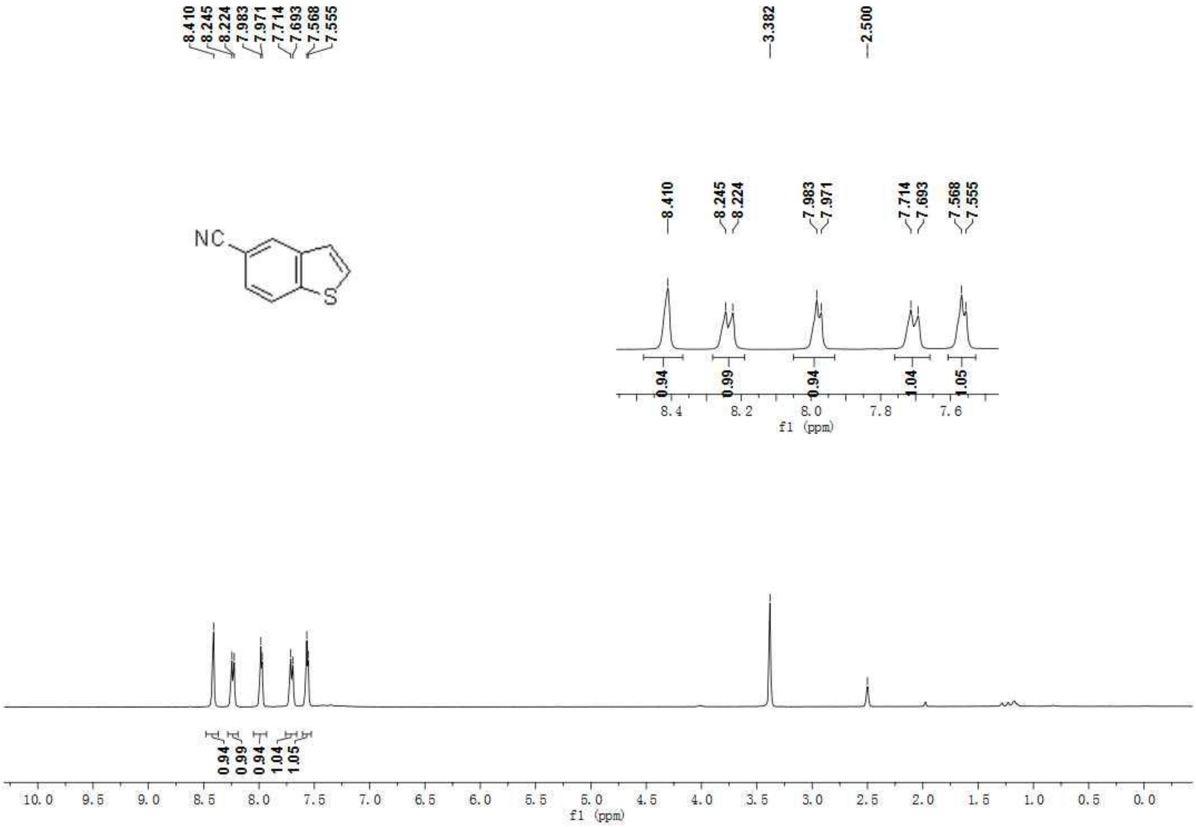
8.410
8.245
8.224
7.983
7.971
7.714
7.693
7.568
7.555
3.382
2.500
NC
S
0.94
0.99
0.94
1.04
1.05
f1 (ppm)

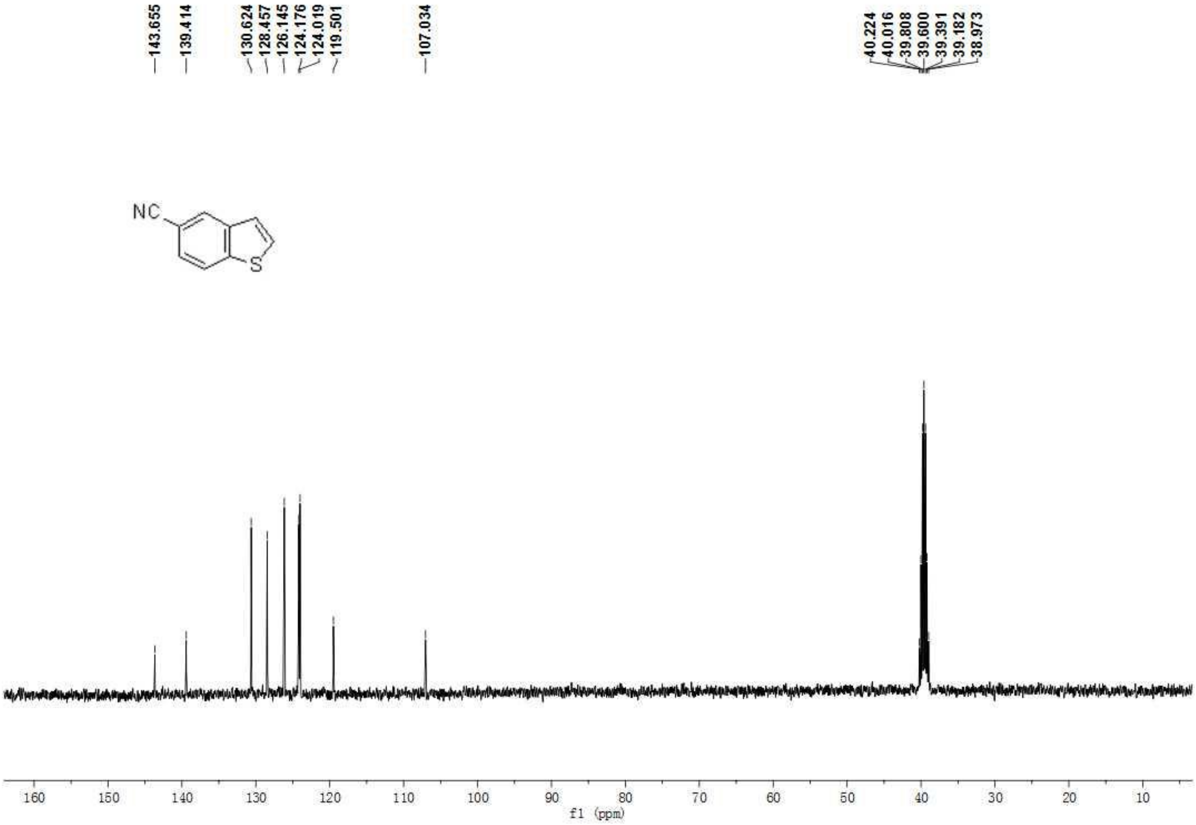
143.655
139.414
130.624
128.457
126.145
124.176
124.019
119.501
107.034
40.224
40.016
39.808
39.600
39.391
39.182
38.973
NC
S
f1 (ppm)

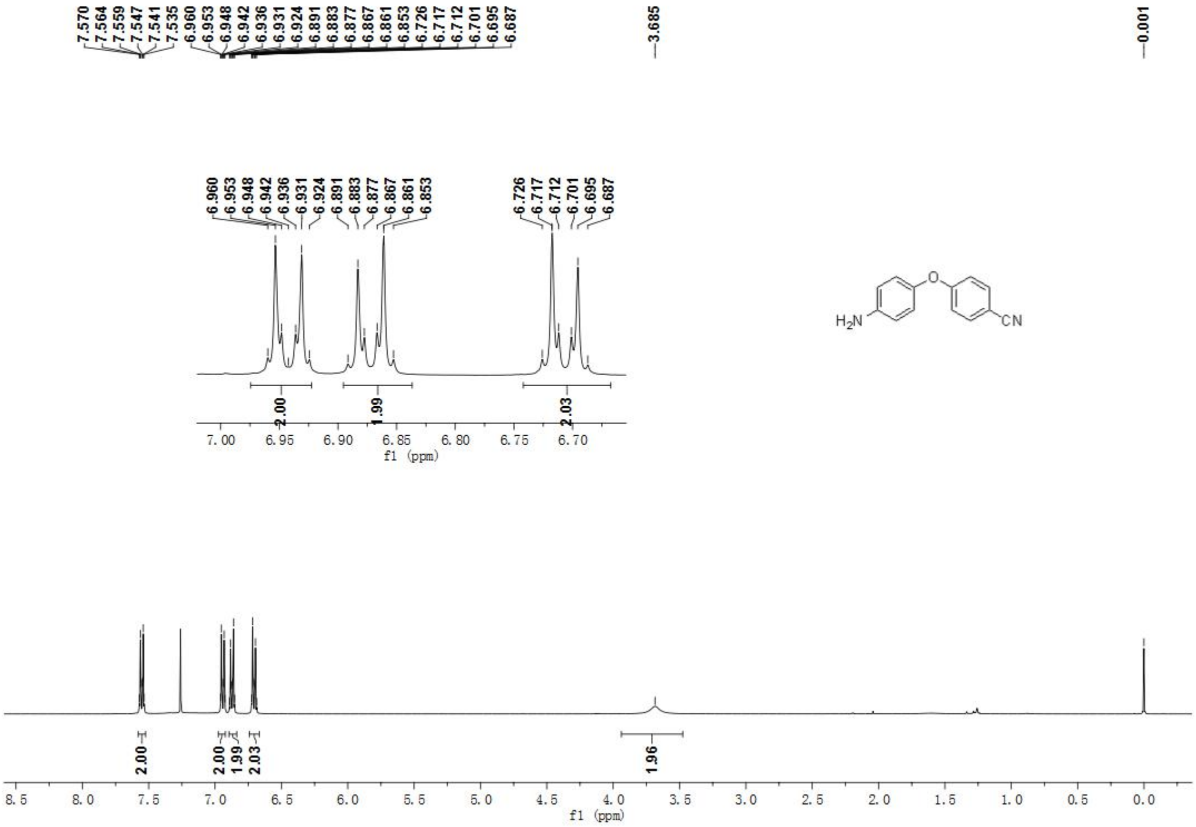
7.570 7.564 7.559 7.547 7.541 7.535 6.960 6.953 6.948 6.942 6.936 6.931 6.924 6.891 6.883 6.877 6.867 6.861 6.853 6.726 6.717 6.712 6.701 6.695 6.687
3.685
0.001
H_2N
CN
2.00
1.99
2.03
7.00 6.95 6.90 6.85 6.80 6.75 6.70
f1 (ppm)
2.00 2.00 1.99 2.03 1.96
8.5 8.0 7.5 7.0 6.5 6.0 5.5 5.0 4.5 4.0 3.5 3.0 2.5 2.0 1.5 1.0 0.5 0.0
f1 (ppm)

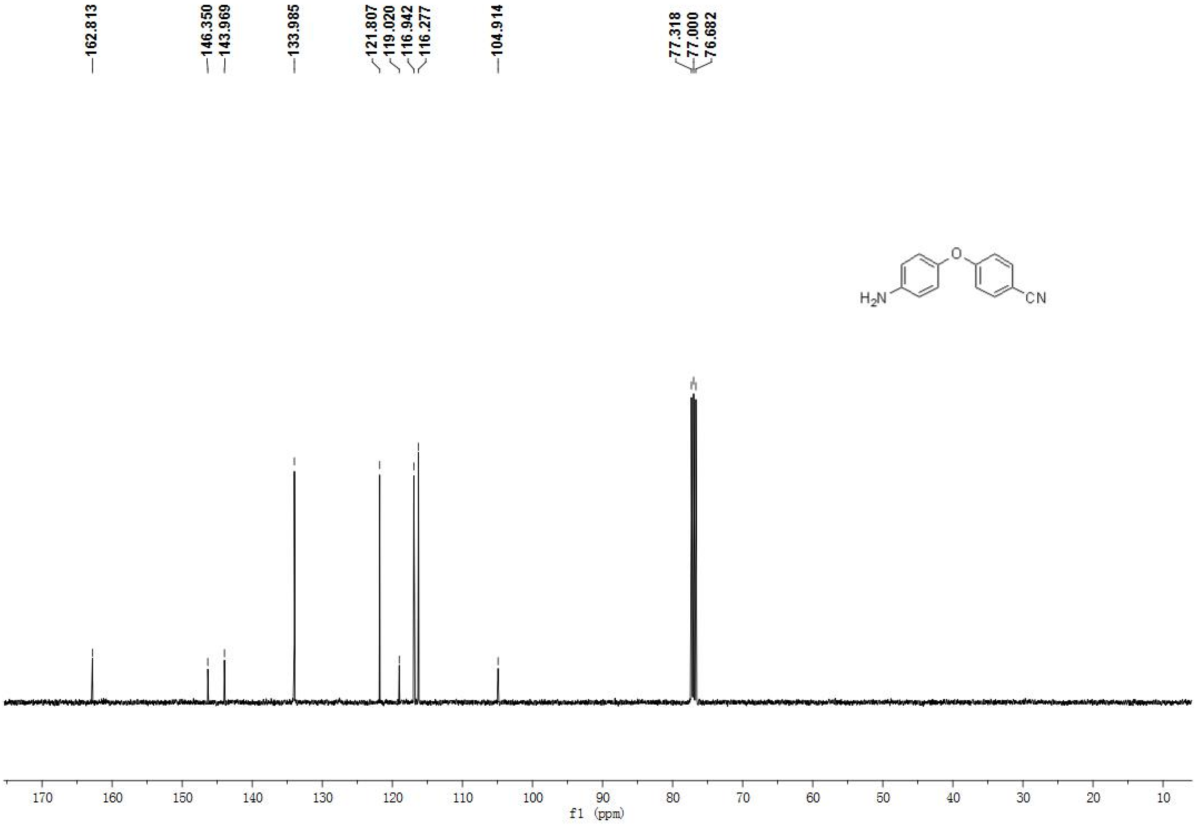
162.813
146.350 143.969
133.985
121.807 119.020 116.942 116.277
104.914
77.318 77.000 76.682
H_2N
CN
170 160 150 140 130 120 110 100 90 80 70 60 50 40 30 20 10
f1 (ppm)

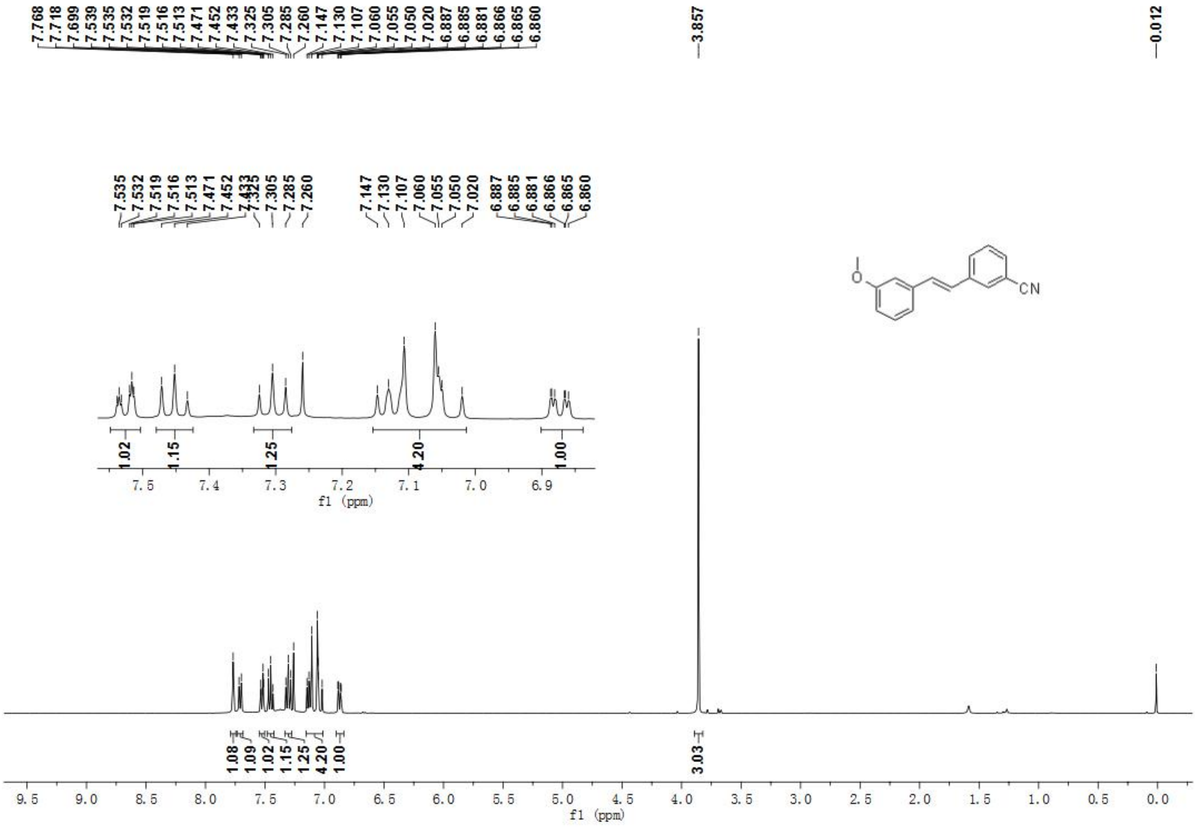
7.768 7.718 7.699 7.539 7.535 7.532 7.519 7.516 7.513 7.471 7.452 7.433 7.325 7.305 7.285 7.260 7.147 7.130 7.107 7.060 7.055 7.050 7.020 6.887 6.885 6.881 6.866 6.865 6.860
3.857
0.012
CN
1.02 1.15 1.25 4.20 1.00
f1 (ppm)
1.08 1.09 1.02 1.15 1.25 4.20 1.00
3.03
f1 (ppm)

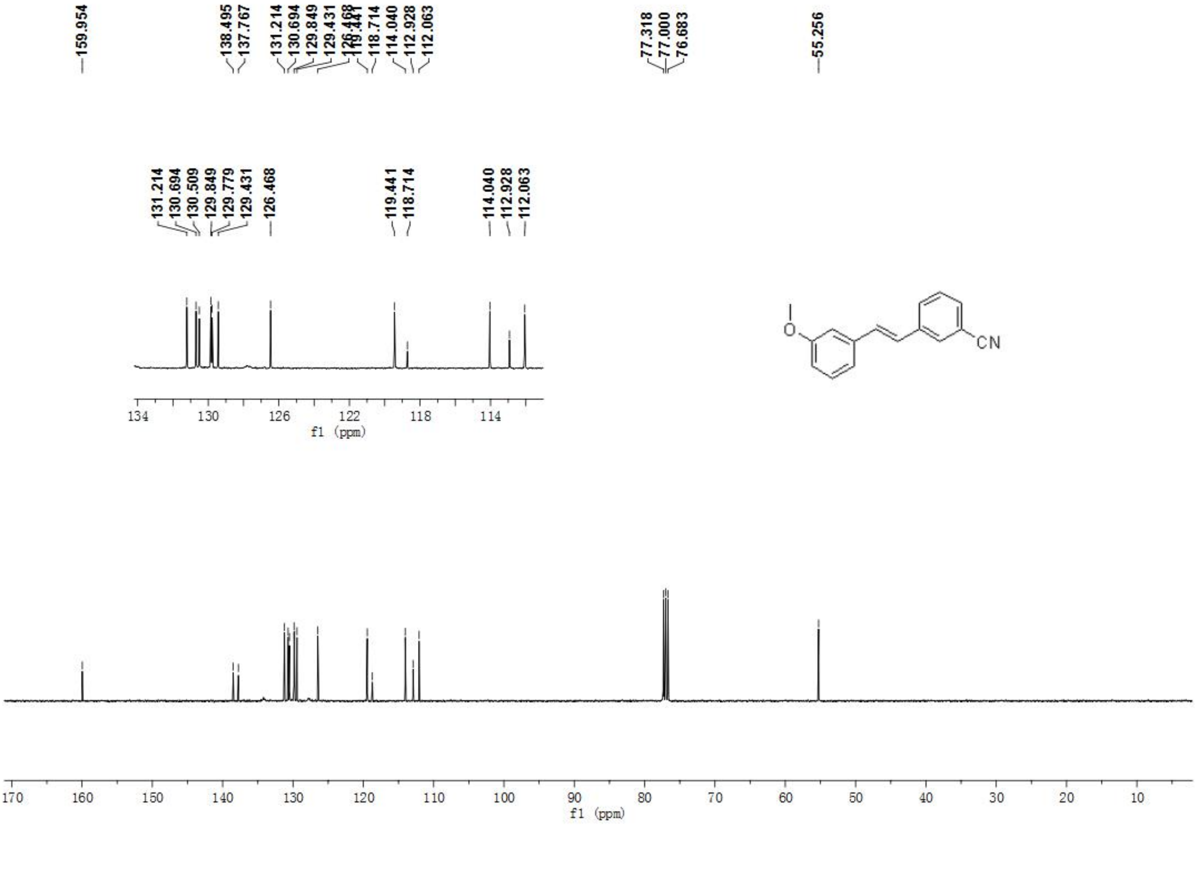
159.954
138.495 137.767 131.214 130.694 129.849 129.431 126.468 119.441 118.714 114.040 112.928 112.063
77.318 77.000 76.683
55.256
131.214 130.694 130.509 129.849 129.779 129.431 126.468 119.441 118.714 114.040 112.928 112.063
CN
f1 (ppm)
f1 (ppm)

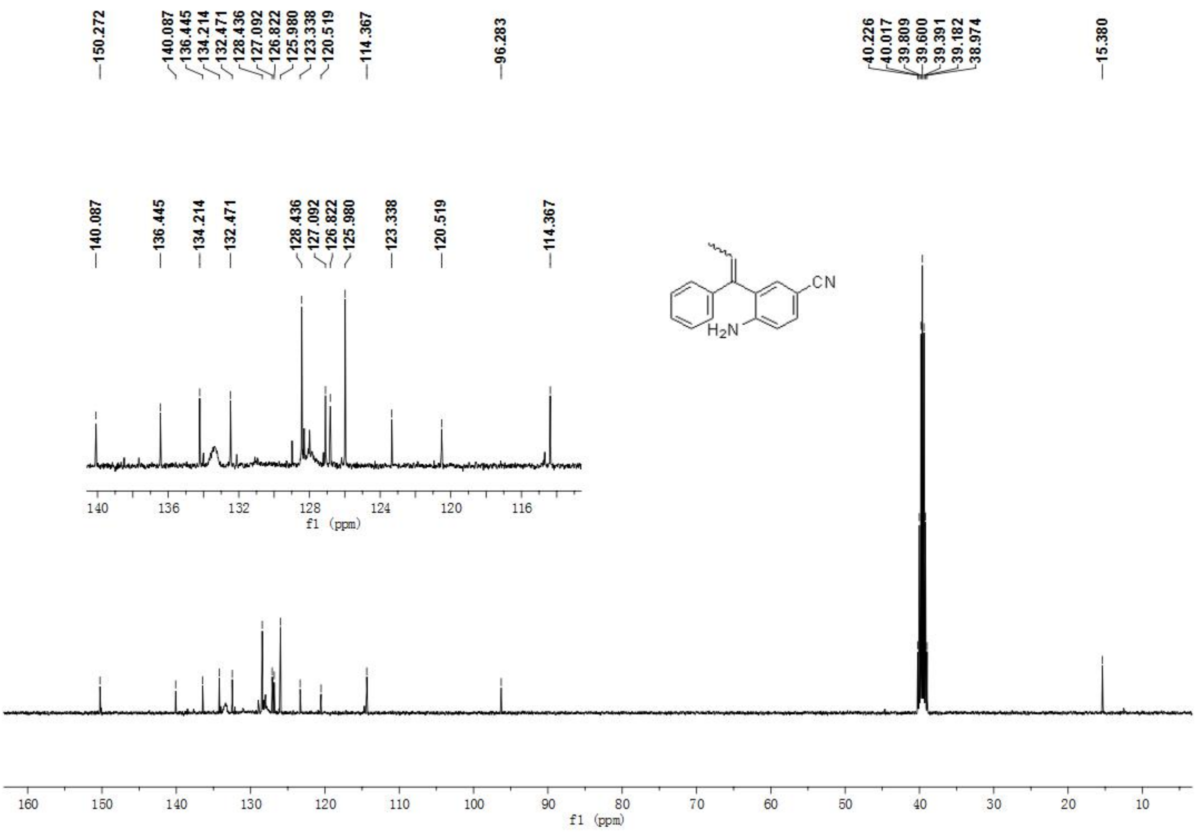
150.272
140.087
136.445
134.214
132.471
128.436
127.092
126.822
125.980
123.338
120.519
114.367
96.283
40.226
40.017
39.809
39.600
39.391
39.182
38.974
15.380
CN
H2N
f1 (ppm)

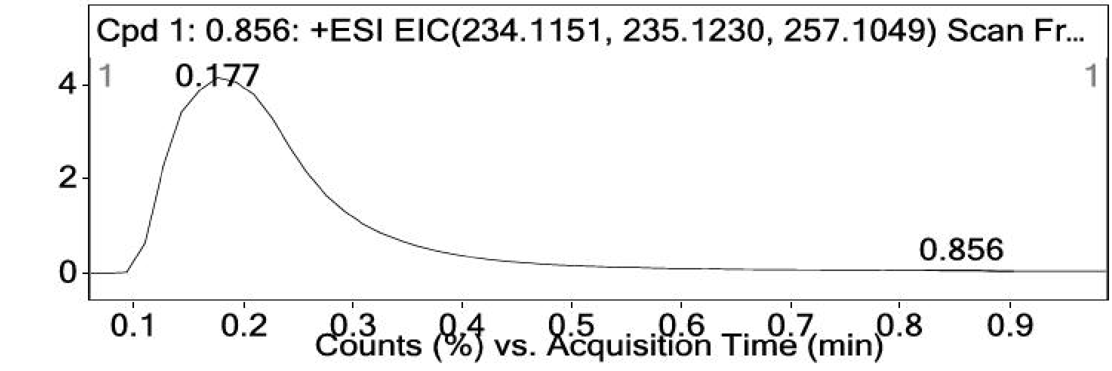
Cpd 1: 0.856: +ESI EIC(234.1151, 235.1230, 257.1049) Scan Fr...
0.177
0.856
Counts (%) vs. Acquisition Time (min)

MS Spectrum

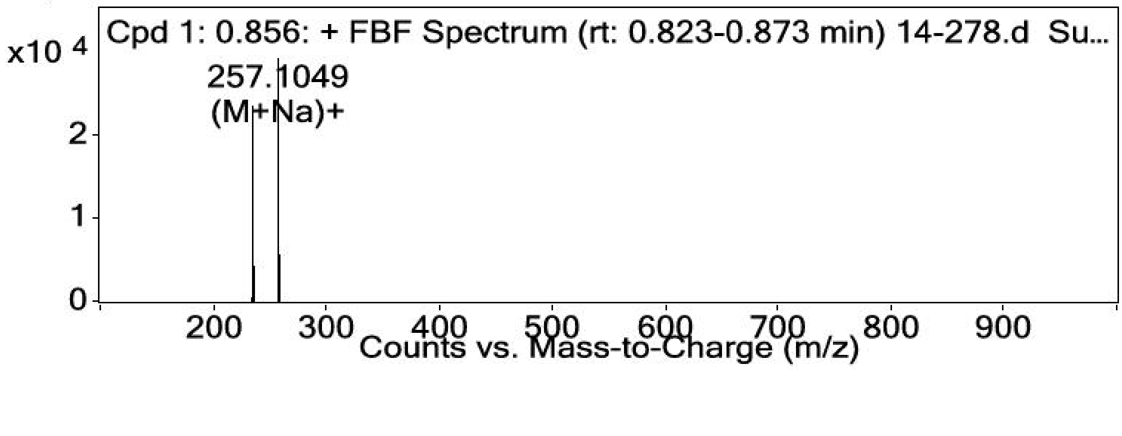
Cpd 1: 0.856: + FBF Spectrum (rt: 0.823-0.873 min) 14-278.d Su...
x10 4
257.1049
(M+Na)+
Counts vs. Mass-to-Charge (m/z)

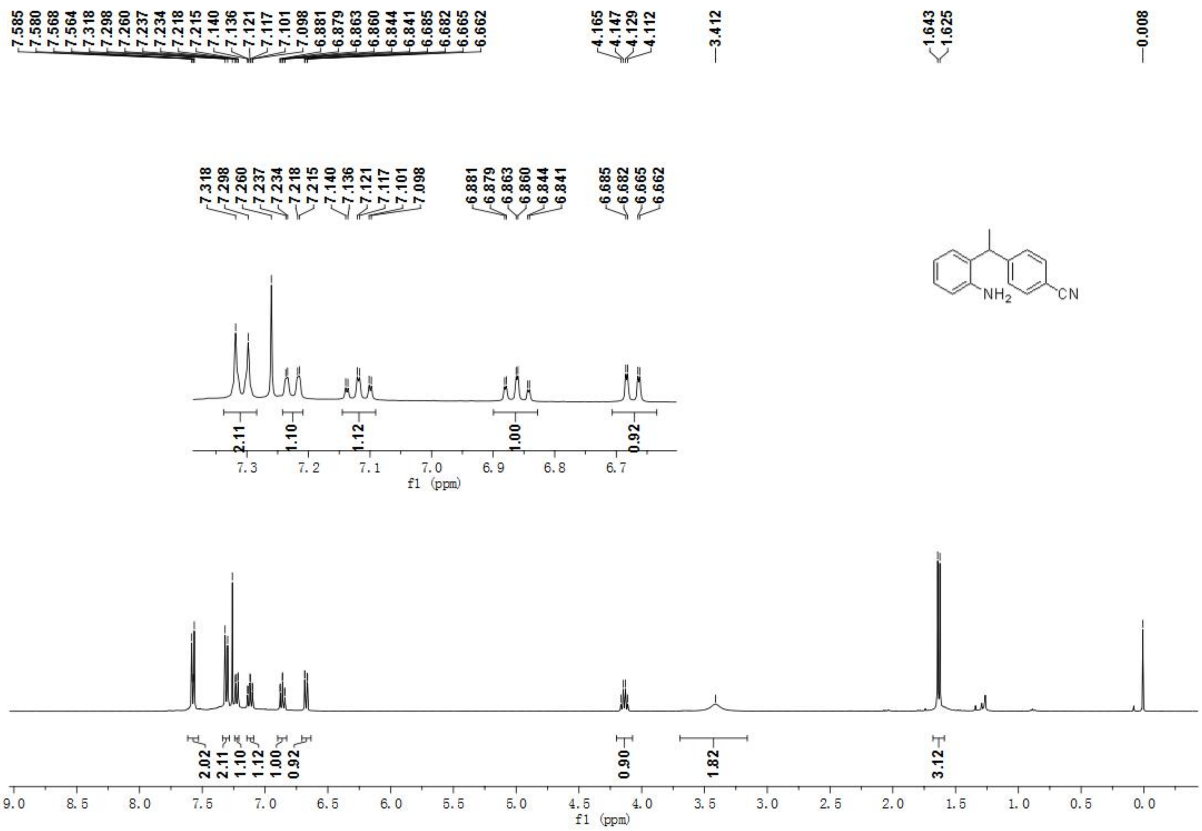
7.585 7.580 7.568 7.564 7.318 7.298 7.260 7.237 7.234 7.218 7.215 7.140 7.136 7.121 7.117 7.101 7.098 6.881 6.879 6.863 6.860 6.844 6.841 6.685 6.682 6.665 6.662
4.165 4.147 4.129 4.112
3.412
1.643 1.625
0.008
NH2
CN
2.11 1.10 1.12 1.00 0.92
f1 (ppm)
2.02 2.11 1.10 1.12 1.00 0.92 0.90 1.82 3.12
f1 (ppm)

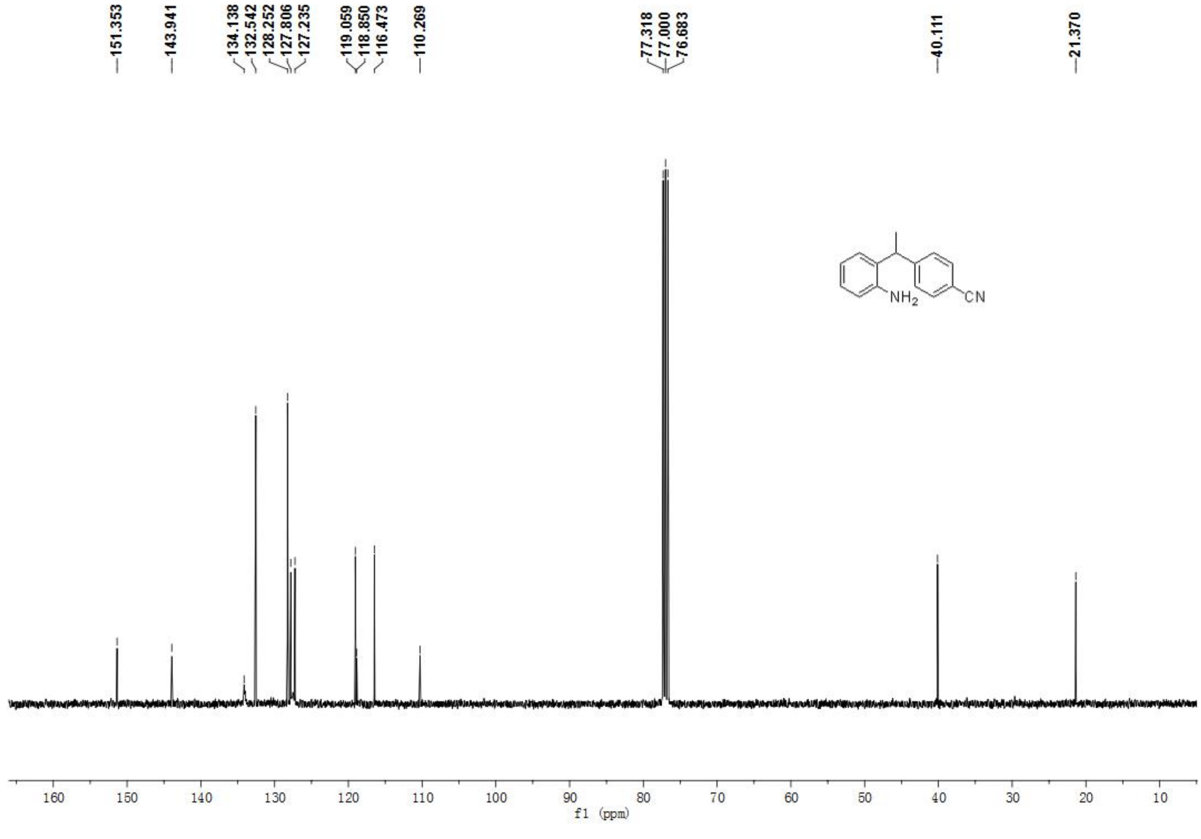
151.353 143.941 134.138 132.542 128.252 127.806 127.235 119.059 118.850 116.473 110.269 77.318 77.000 76.683 40.111 21.370
NH2
CN
f1 (ppm)

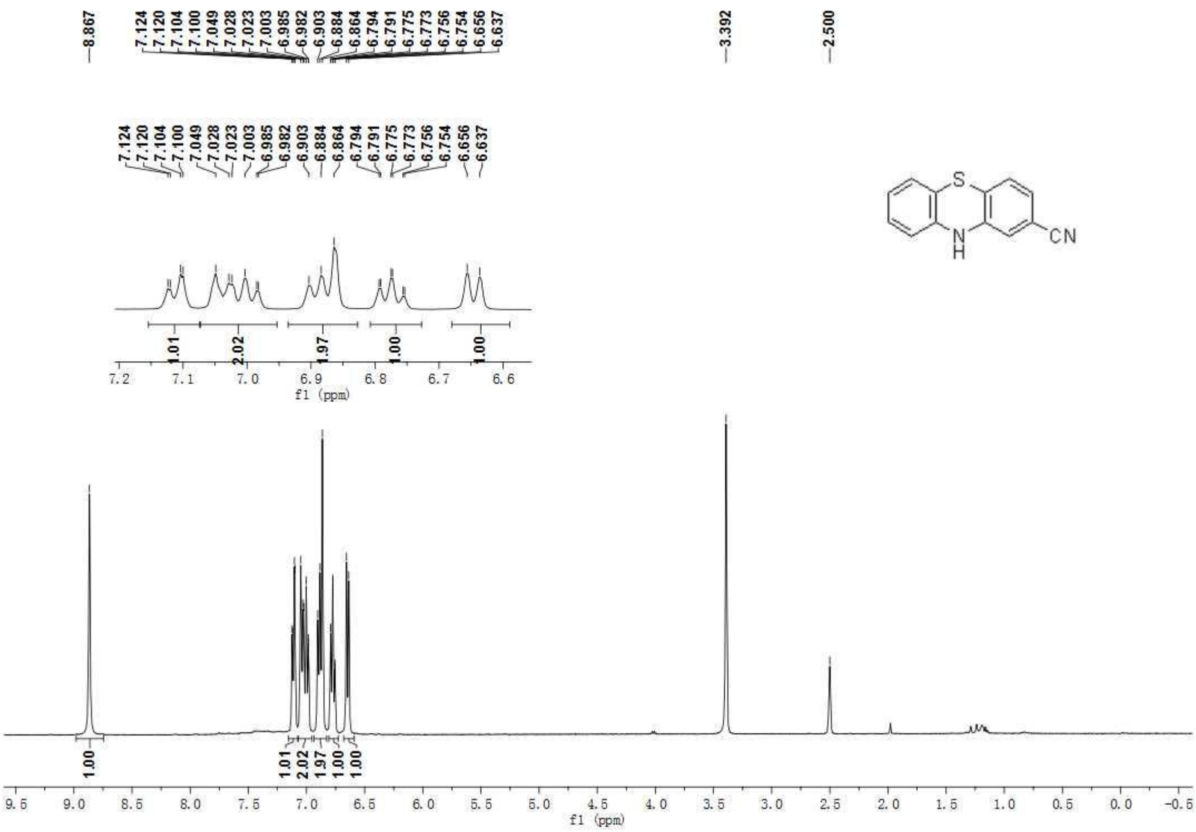
8.867
7.124
7.120
7.104
7.100
7.049
7.028
7.023
7.003
6.985
6.982
6.903
6.884
6.864
6.794
6.791
6.775
6.773
6.756
6.754
6.656
6.637
3.392
2.500
S
N
H
CN
1.01
2.02
1.97
1.00
1.00
f1 (ppm)

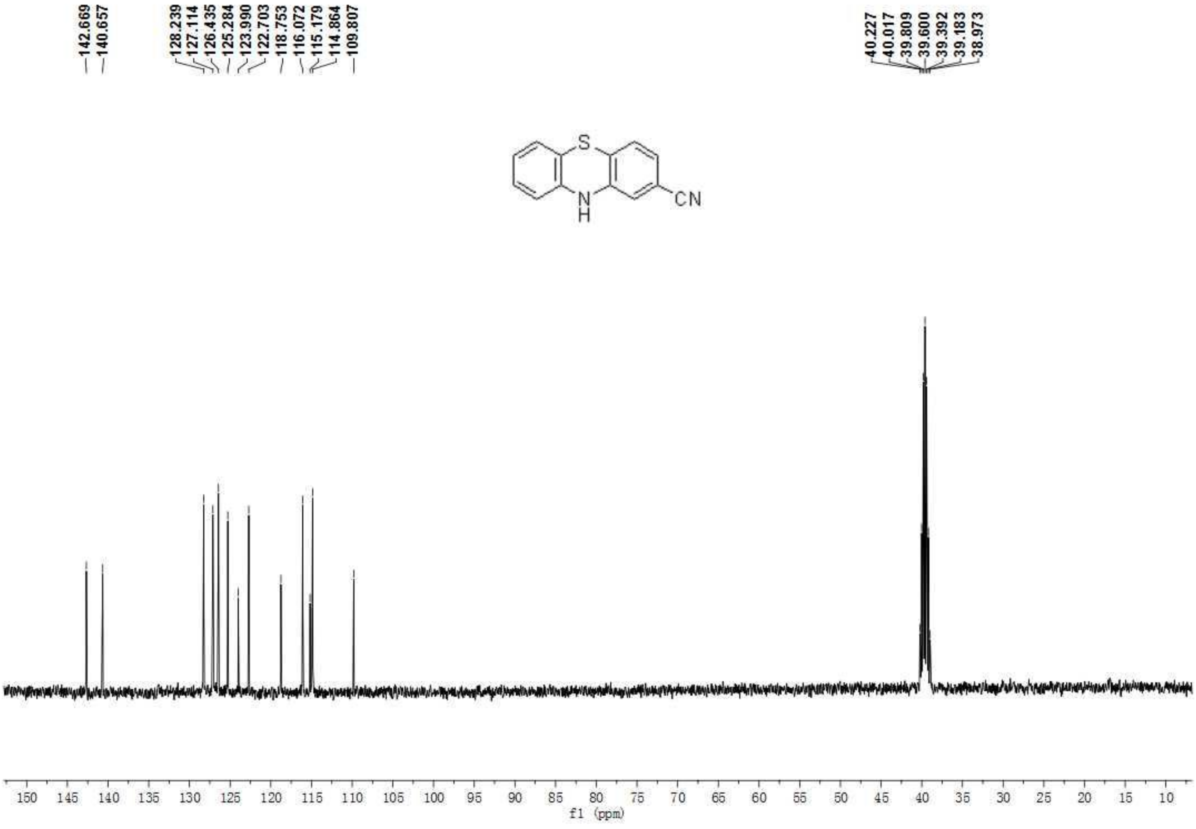
142.669
140.657
128.239
127.114
126.435
125.284
123.990
122.703
118.753
116.072
115.179
114.864
109.807
40.227
40.017
39.809
39.600
39.392
39.183
38.973
S
N
H
CN
f1 (ppm)

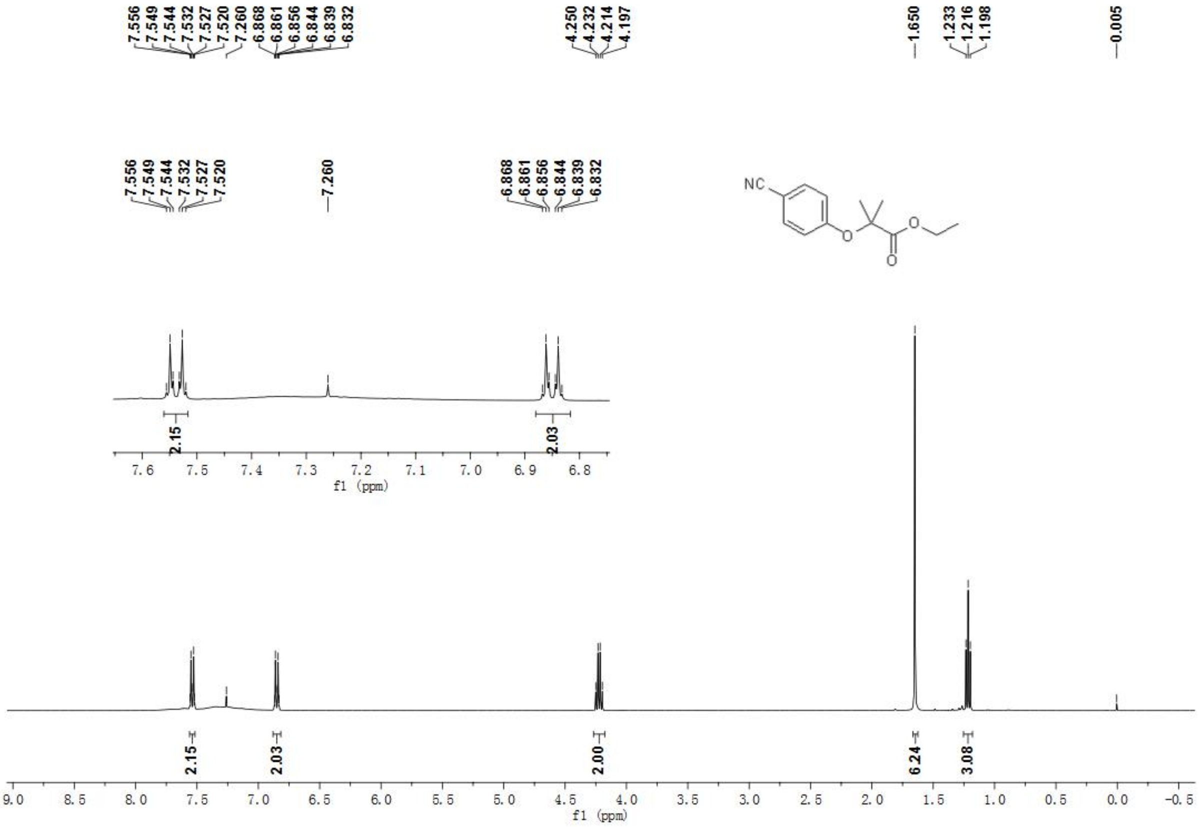
7.556
7.549
7.544
7.532
7.527
7.520
7.260
6.868
6.861
6.856
6.844
6.839
6.832
4.250
4.232
4.214
4.197
1.650
1.233
1.216
1.198
0.005
NC
O
O
O
2.15
2.03
2.00
6.24
3.08
f1 (ppm)

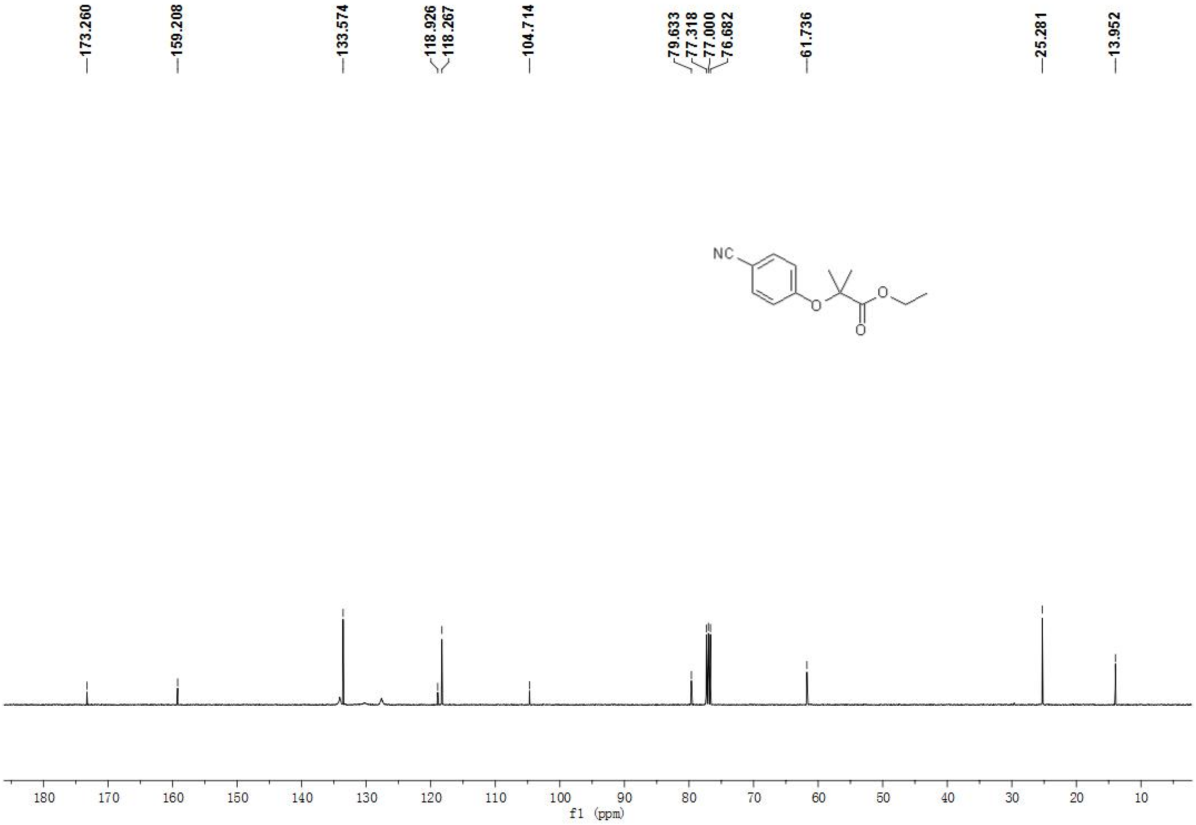
173.260
159.208
133.574
118.926
118.267
104.714
79.633
77.318
77.000
76.682
61.736
25.281
13.952
NC
O
O
O
f1 (ppm)

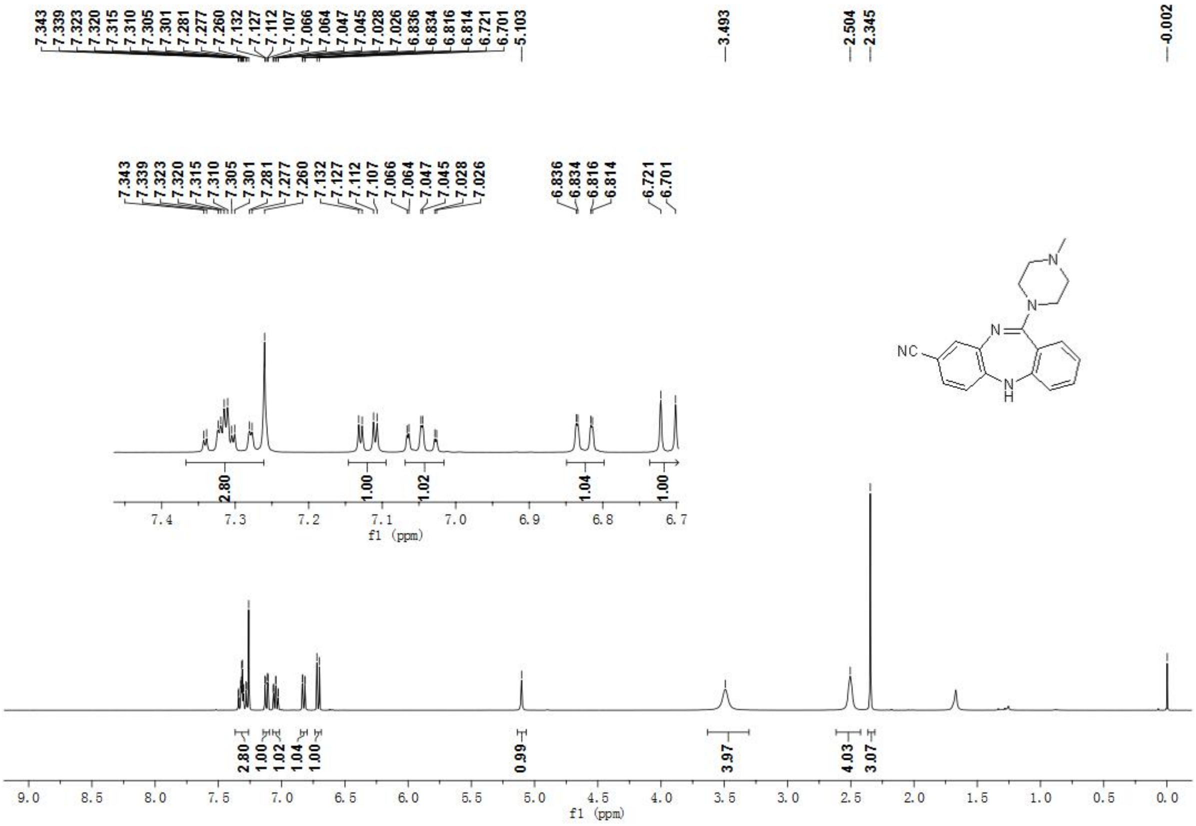
7.343 7.339 7.323 7.320 7.315 7.310 7.305 7.301 7.281 7.277 7.260 7.132 7.127 7.112 7.107 7.066 7.064 7.047 7.045 7.028 7.026 6.836 6.834 6.816 6.814 6.721 6.701 5.103
3.493
2.504 2.345
-0.002
2.80 1.00 1.02 1.04 1.00 0.99 3.97 4.03 3.07
f1 (ppm)
NC
N
H

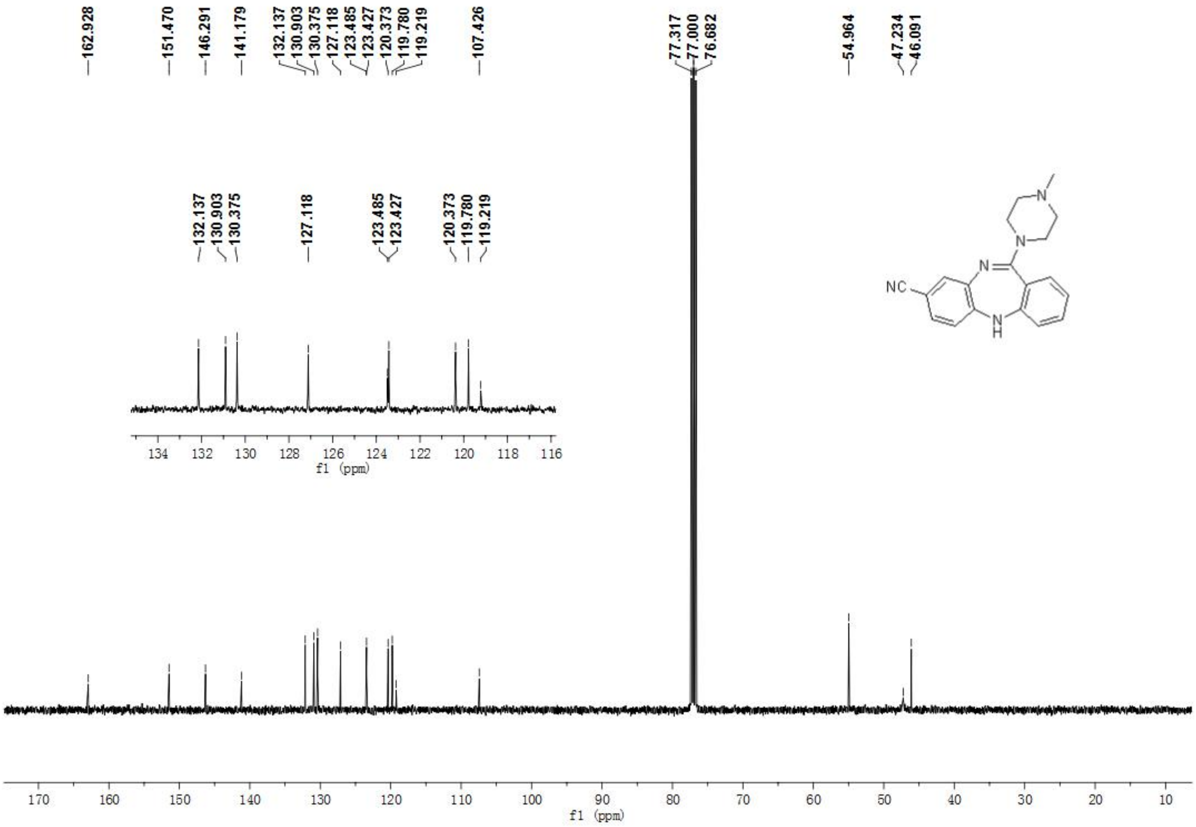
162.928 151.470 146.291 141.179 132.137 130.903 130.375 127.118 123.485 123.427 120.373 119.780 119.219 107.426
77.317 77.000 76.682
54.964 47.234 46.091
f1 (ppm)
NC
N
H

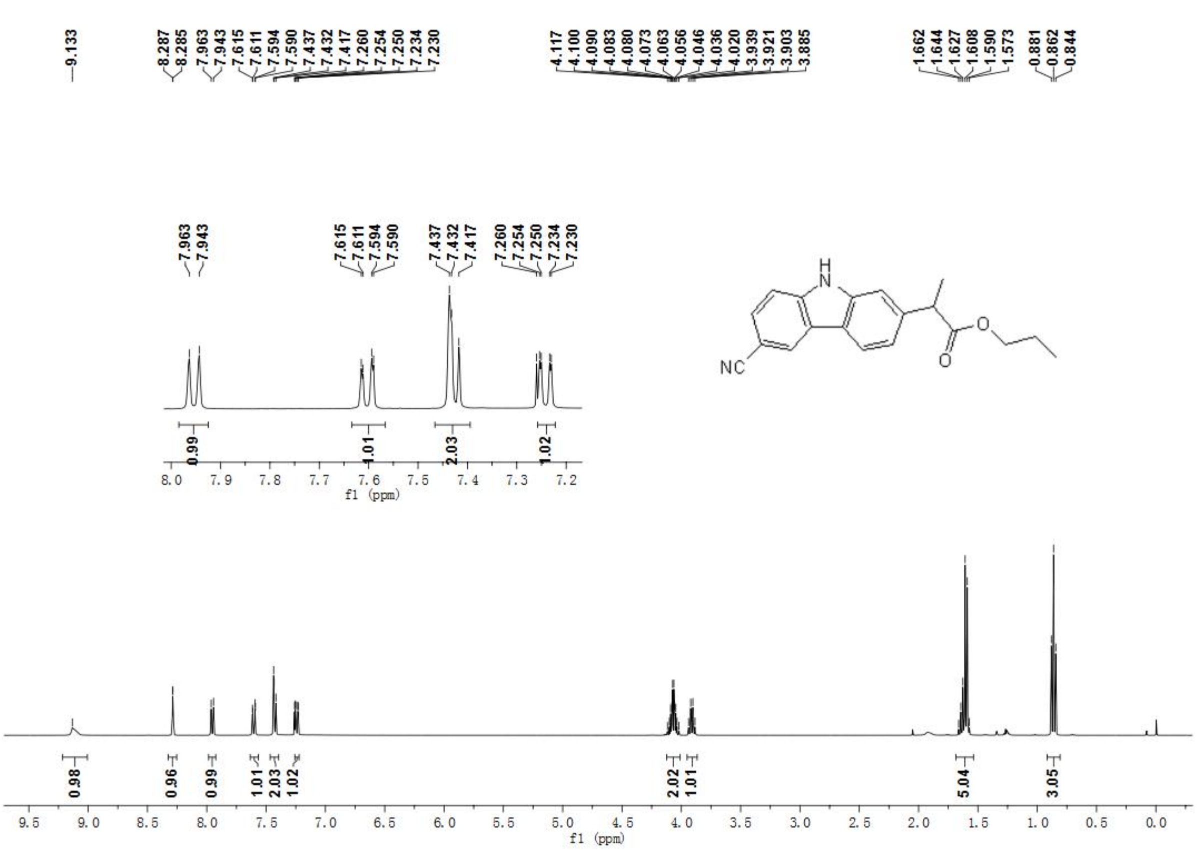
9.133
8.287
8.285
7.963
7.943
7.615
7.611
7.594
7.590
7.437
7.432
7.417
7.260
7.254
7.250
7.234
7.230
4.117
4.100
4.090
4.083
4.080
4.073
4.063
4.056
4.046
4.036
4.020
3.939
3.921
3.903
3.885
1.662
1.644
1.627
1.608
1.590
1.573
0.881
0.862
0.844
H
NC
O
0.99
1.01
2.03
1.02
0.98
0.96
2.02
5.04
3.05
f1 (ppm)

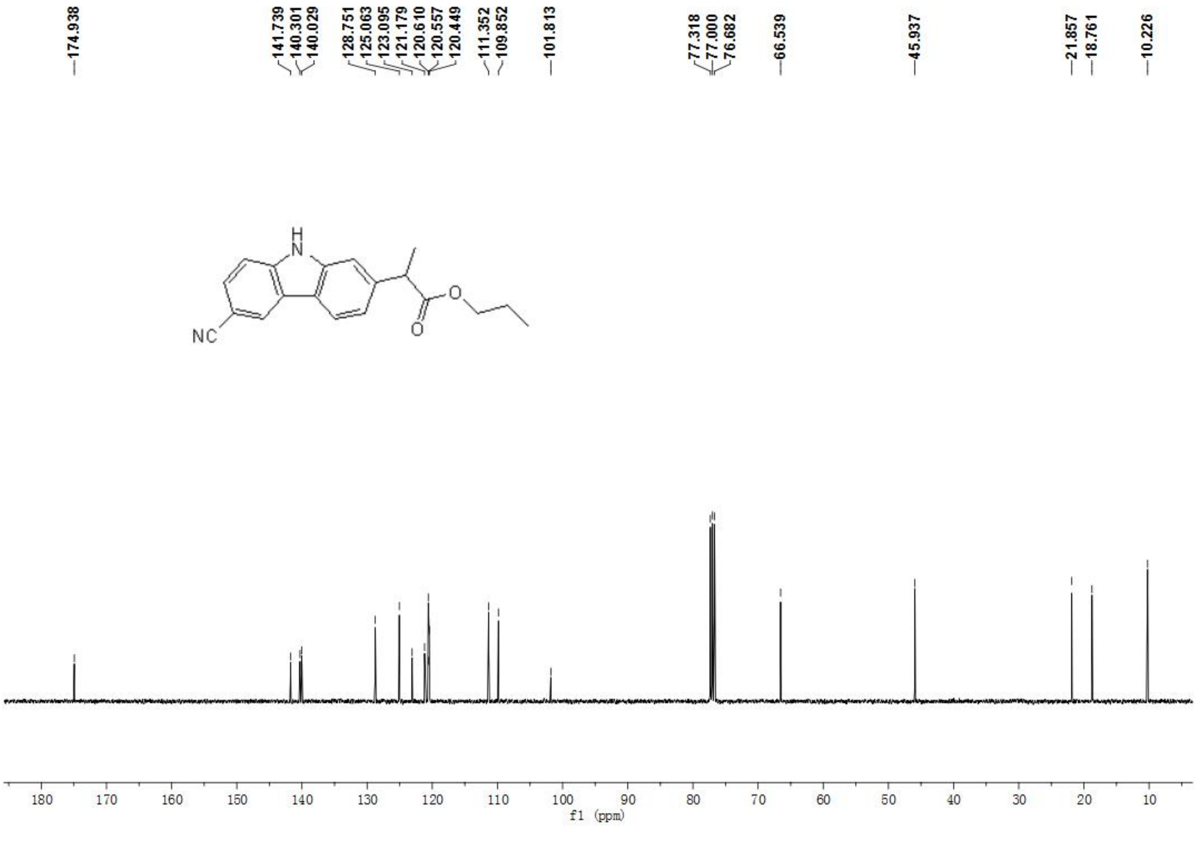
174.938
141.739
140.301
140.029
128.751
125.063
123.095
121.179
120.610
120.557
120.449
111.352
109.852
101.813
77.318
77.000
76.682
66.539
45.937
21.857
18.761
10.226
H
NC
O
f1 (ppm)

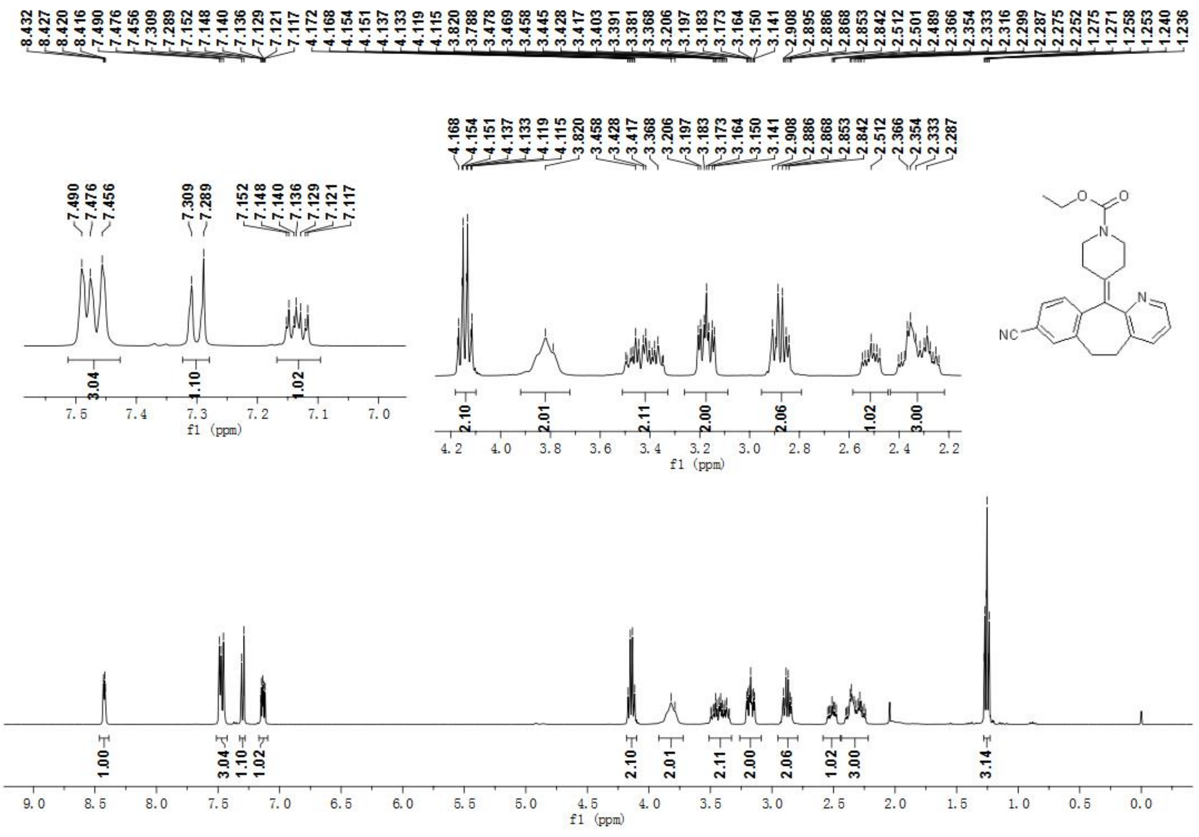

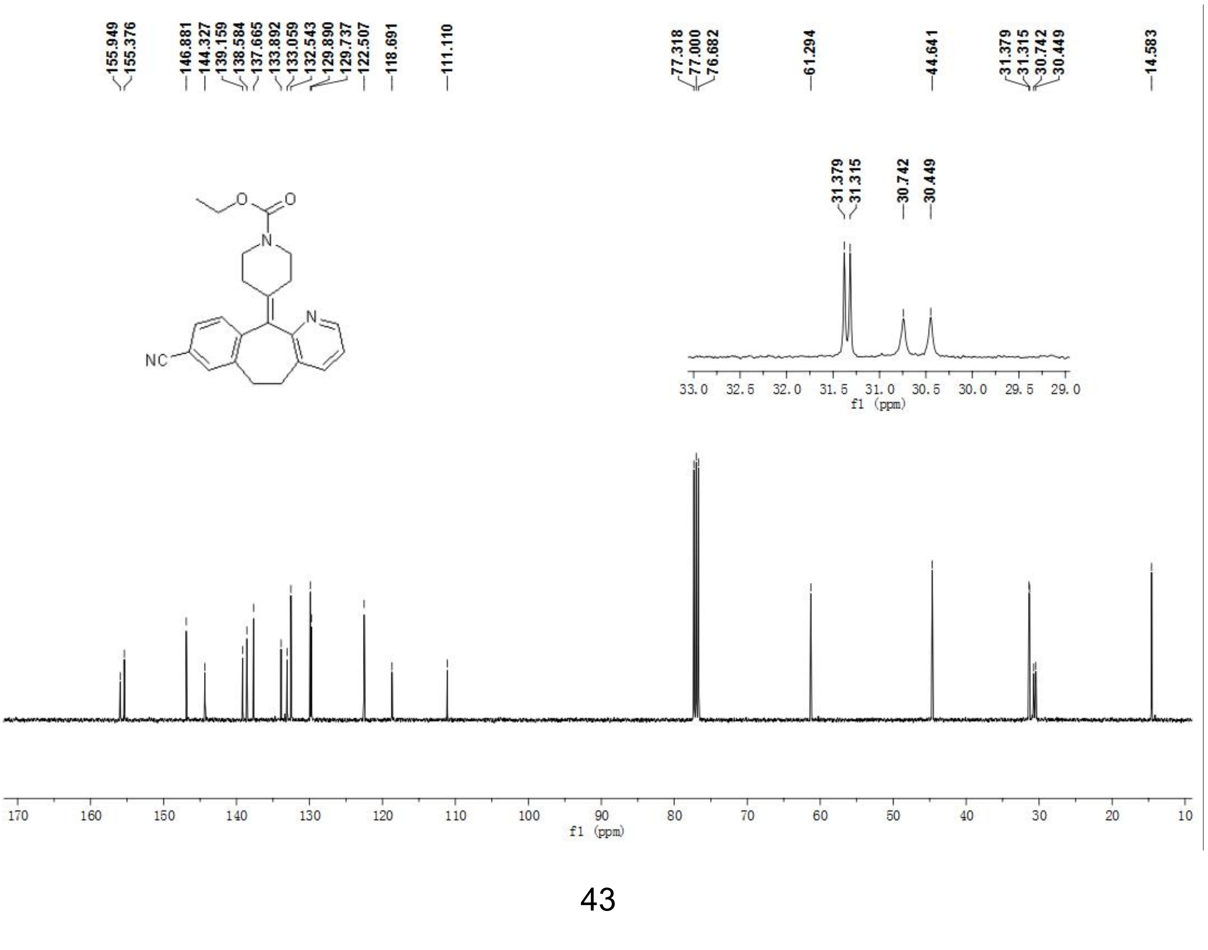

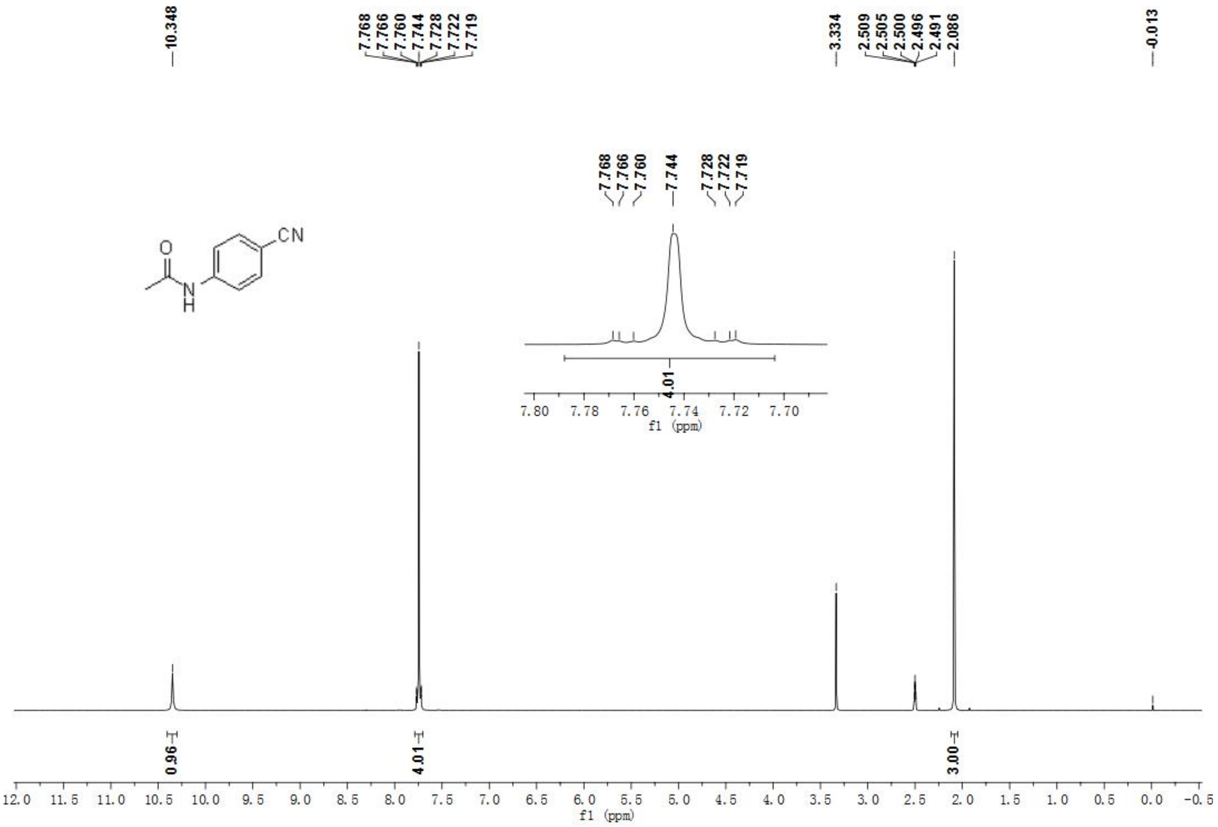
10.348
7.768
7.766
7.760
7.744
7.728
7.722
7.719
3.334
2.509
2.505
2.500
2.496
2.491
2.086
-0.013
CN
O
N
H
4.01
7.80 7.78 7.76 7.74 7.72 7.70
f1 (ppm)
0.96
4.01
3.00
12.0 11.5 11.0 10.5 10.0 9.5 9.0 8.5 8.0 7.5 7.0 6.5 6.0 5.5 5.0 4.5 4.0 3.5 3.0 2.5 2.0 1.5 1.0 0.5 0.0 -0.5
f1 (ppm)

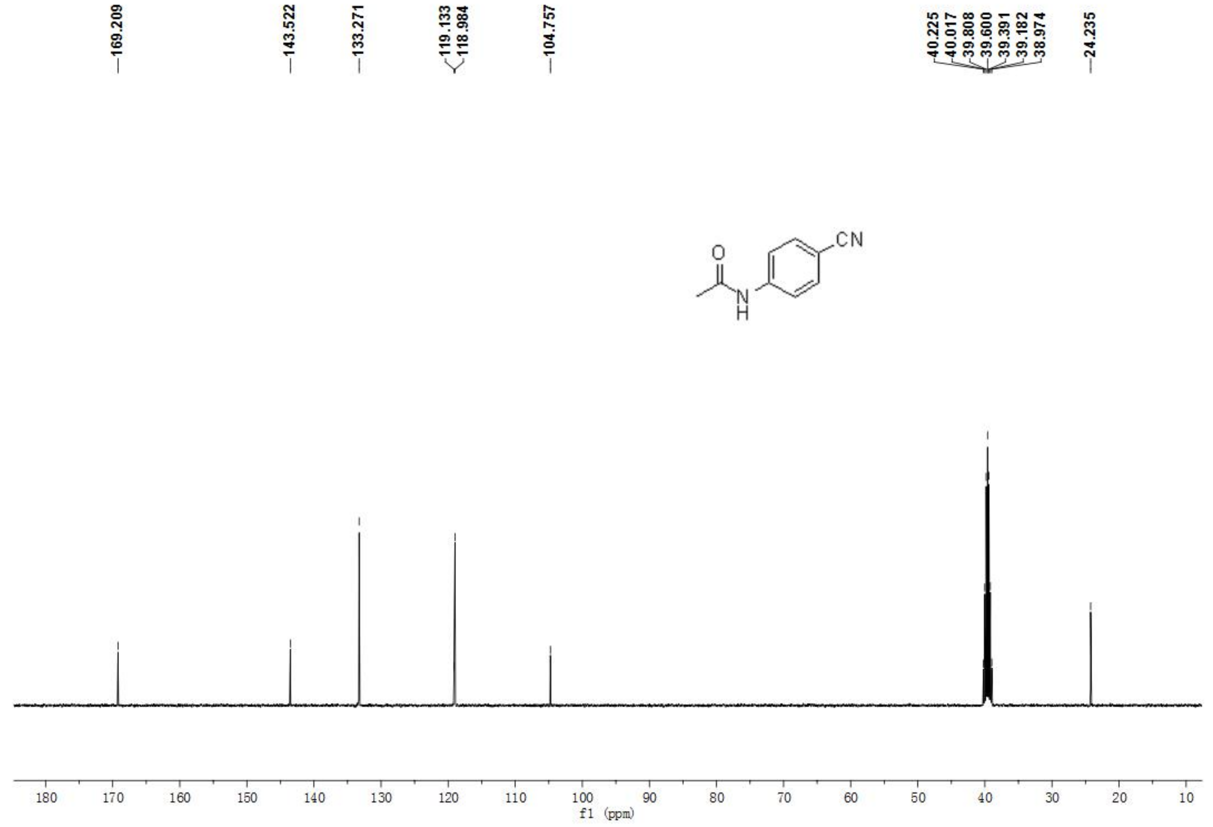
169.209
143.522
133.271
119.133
118.984
104.757
40.225
40.017
39.808
39.600
39.391
39.182
38.974
24.235
CN
O
N
H
180 170 160 150 140 130 120 110 100 90 80 70 60 50 40 30 20 10
f1 (ppm)

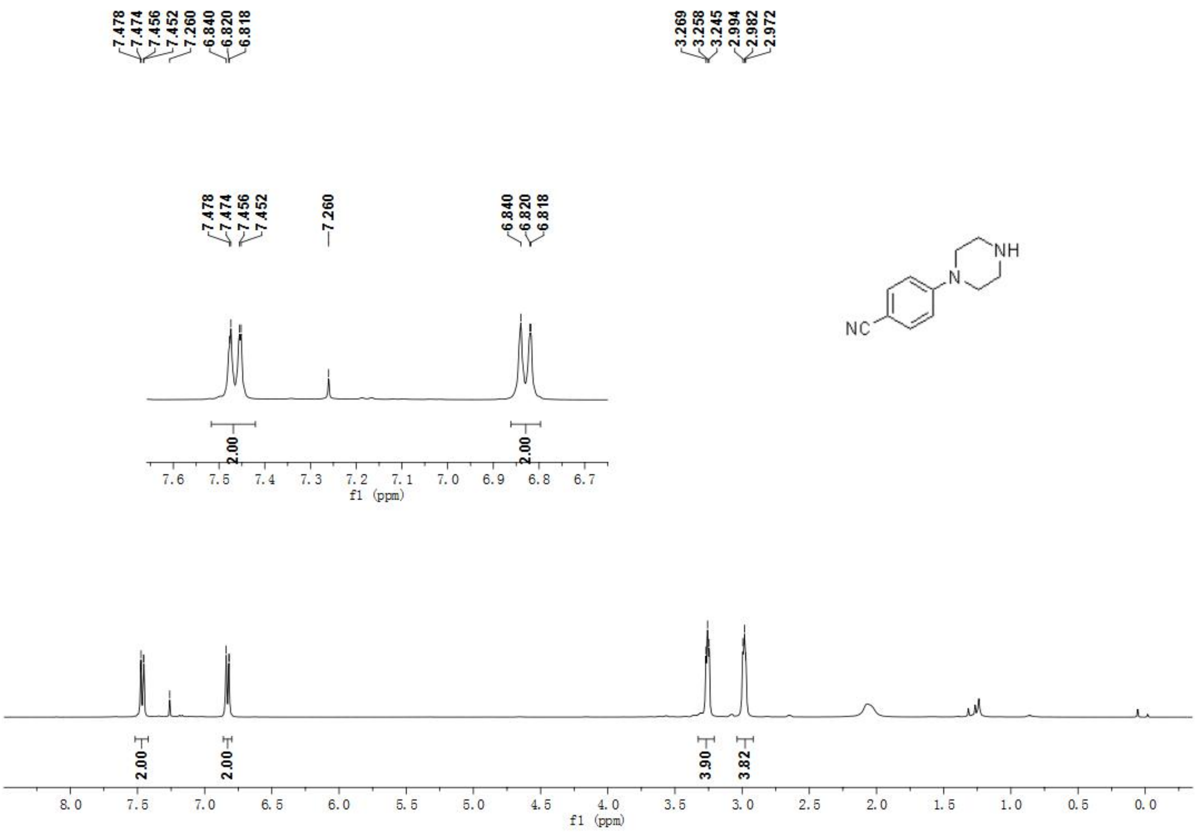
7.478
7.474
7.456
7.452
7.260
6.840
6.820
6.818
3.269
3.258
3.245
2.994
2.982
2.972
NH
N
NC
2.00
2.00
3.90
3.82
f1 (ppm)

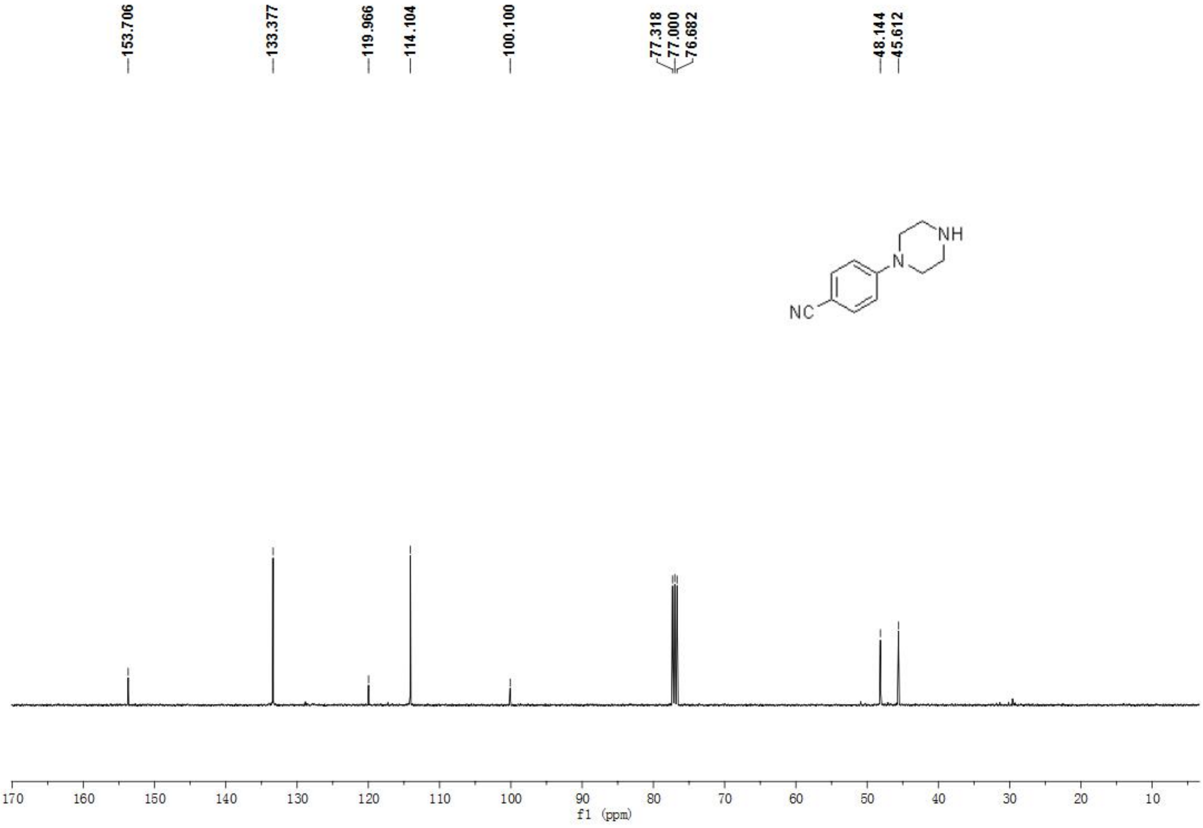
153.706
133.377
119.966
114.104
100.100
77.318
77.000
76.682
48.144
45.612
NH
N
NC
f1 (ppm)

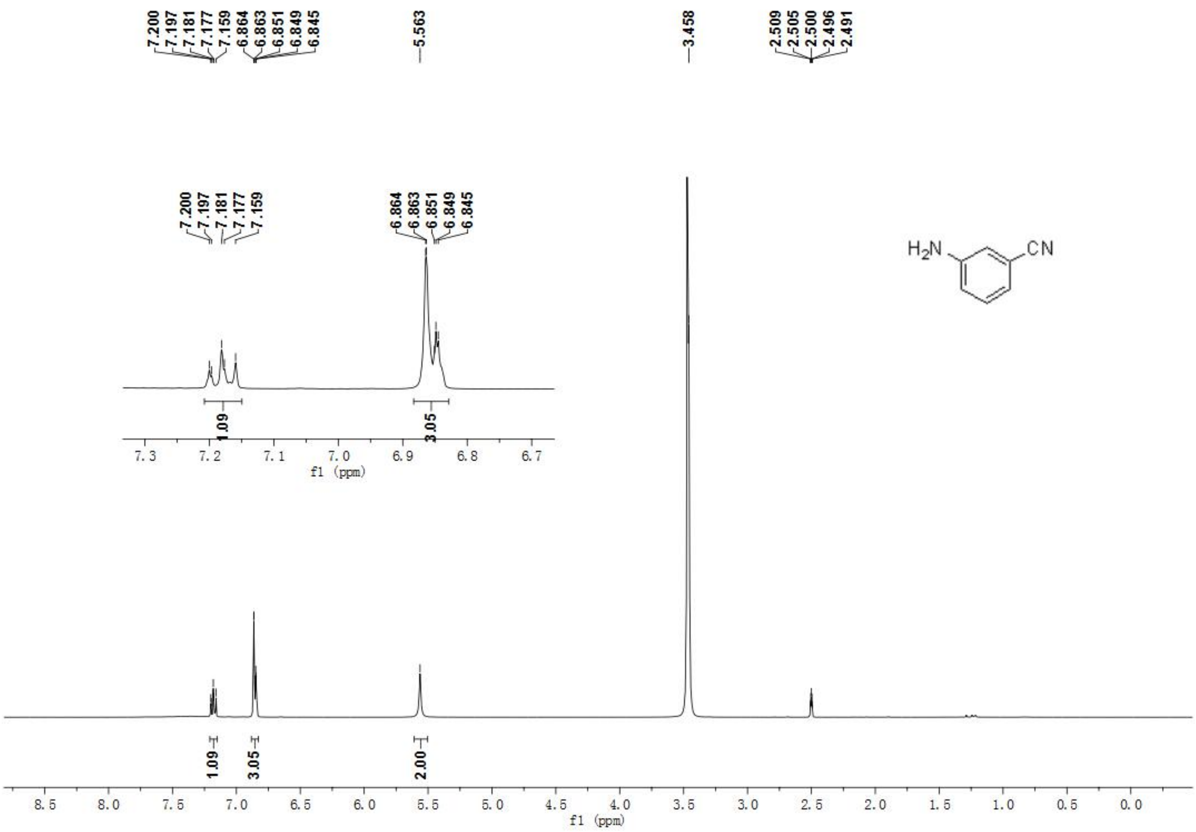
7.200
7.197
7.181
7.177
7.159
6.864
6.863
6.851
6.849
6.845
5.563
3.458
2.509
2.505
2.500
2.496
2.491
1.09
3.05
2.00
f1 (ppm)

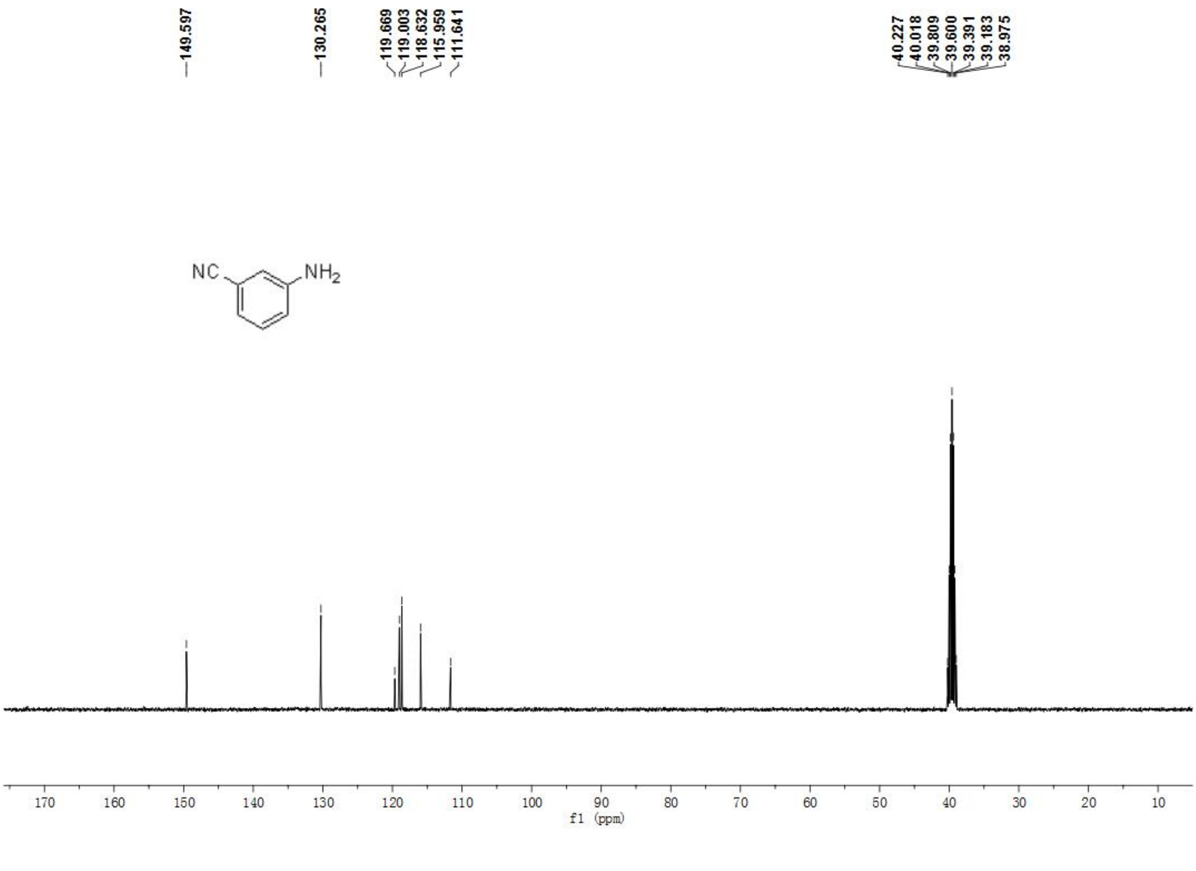
149.597
130.265
119.669
119.003
118.632
115.959
111.641
40.227
40.018
39.809
39.600
39.391
39.183
38.975
f1 (ppm)

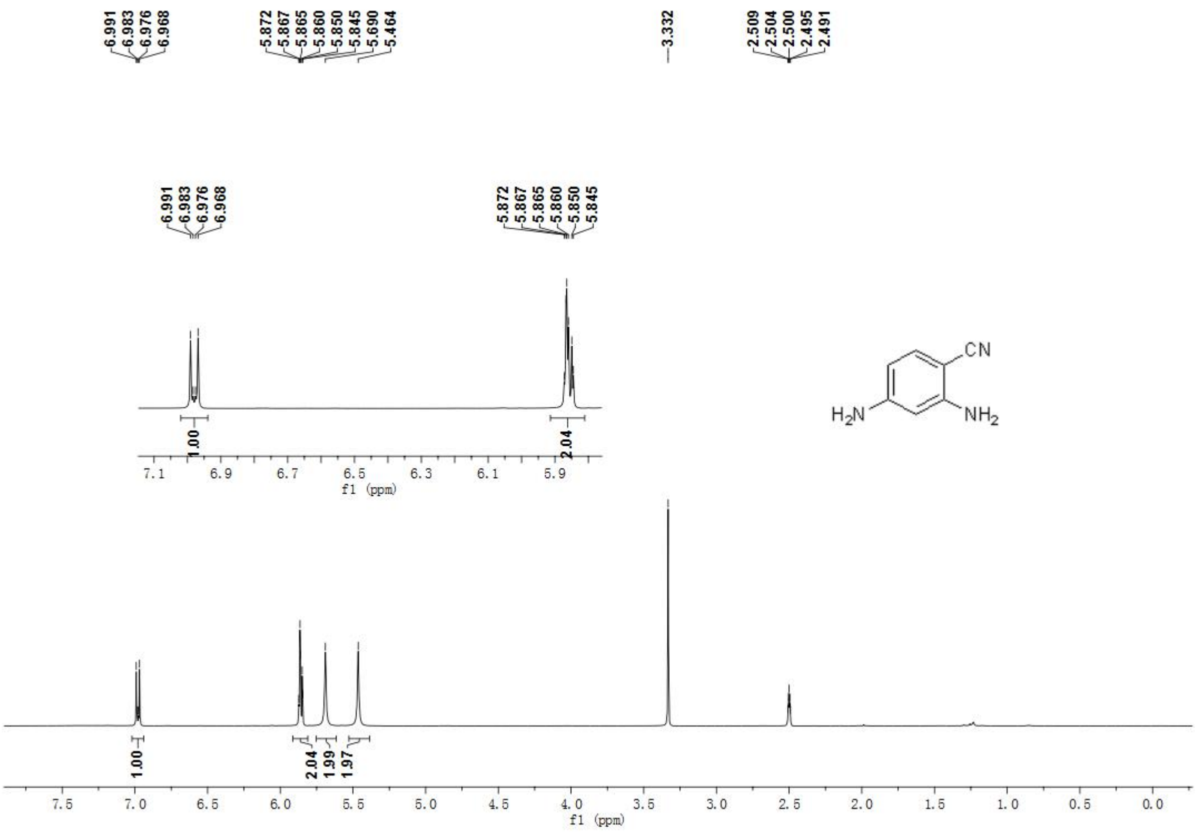
6.991
6.983
6.976
6.968
5.872
5.867
5.865
5.860
5.850
5.845
5.690
5.464
3.332
2.509
2.504
2.500
2.495
2.491
CN
H2N
NH2
1.00
2.04
1.99
1.97
f1 (ppm)

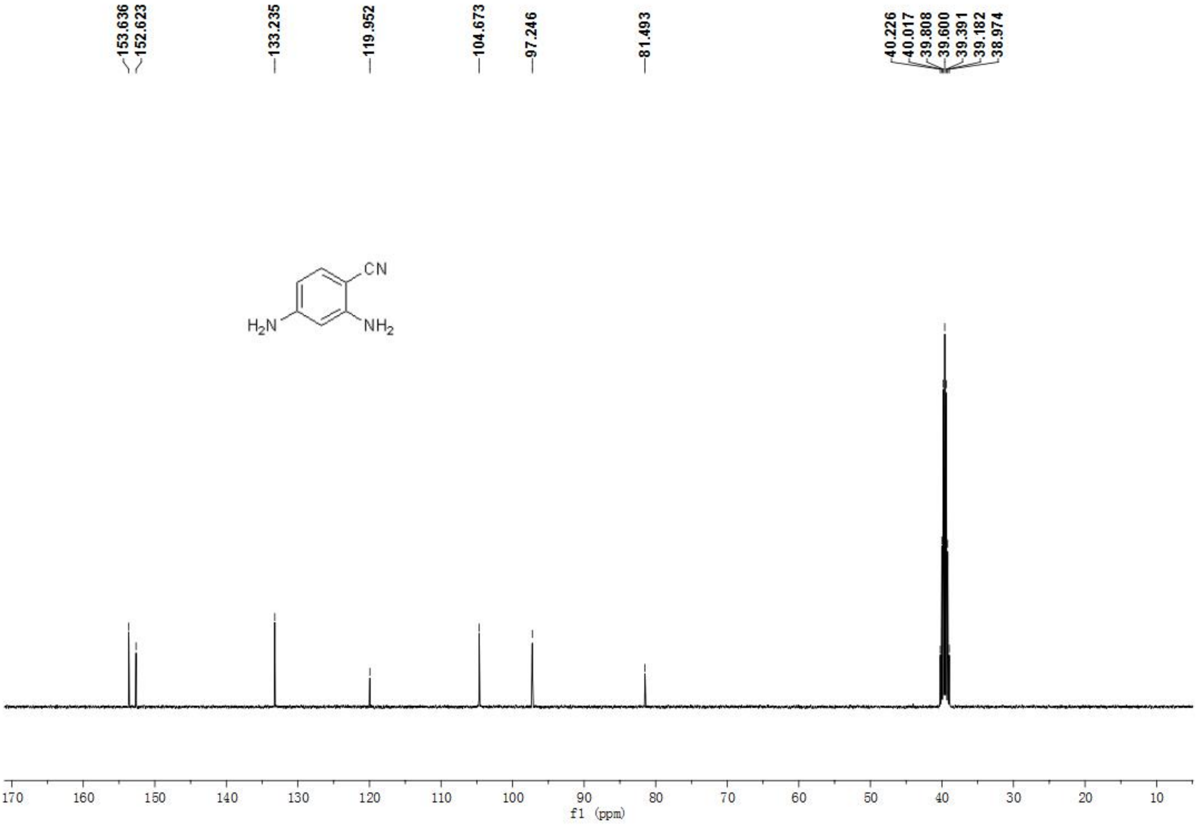
153.636
152.623
133.235
119.952
104.673
97.246
81.493
40.226
40.017
39.808
39.600
39.391
39.182
38.974
CN
H2N
NH2
f1 (ppm)

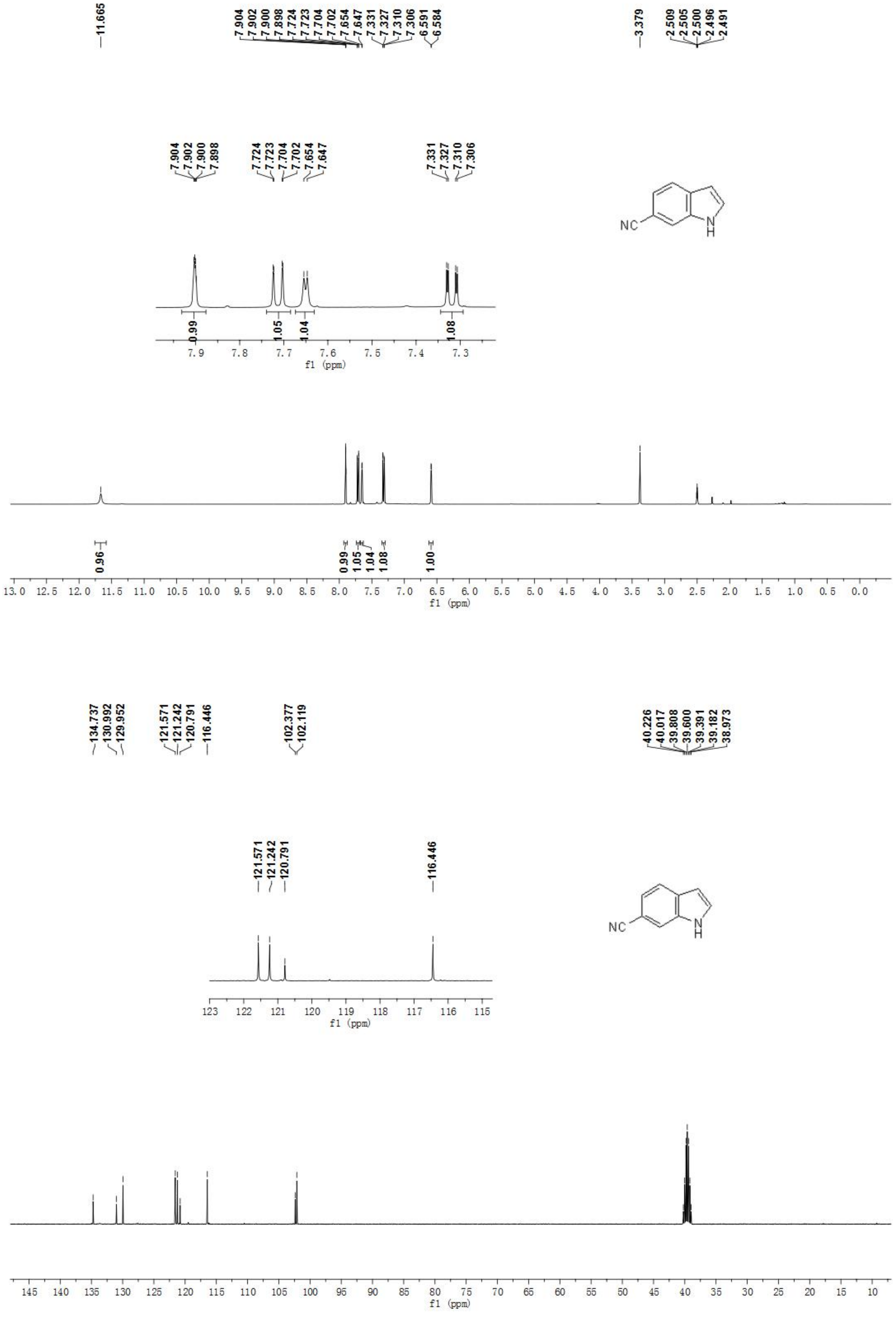
NC
H
f1 (ppm)

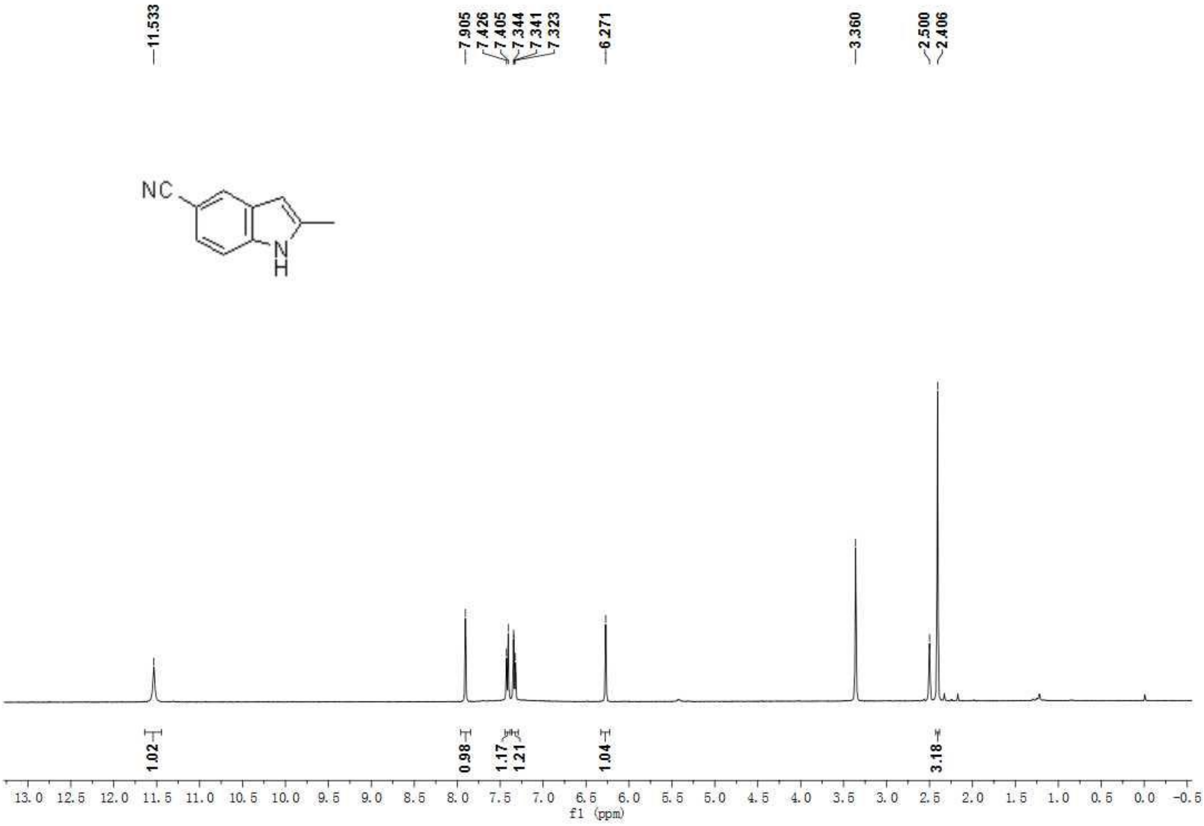

11.533
7.905
7.426
7.405
7.344
7.341
7.323
6.271
3.360
2.500
2.406
NC
N
H
1.02
0.98
1.17
1.21
1.04
3.18
13.0 12.5 12.0 11.5 11.0 10.5 10.0 9.5 9.0 8.5 8.0 7.5 7.0 6.5 6.0 5.5 5.0 4.5 4.0 3.5 3.0 2.5 2.0 1.5 1.0 0.5 0.0 -0.5
f1 (ppm)

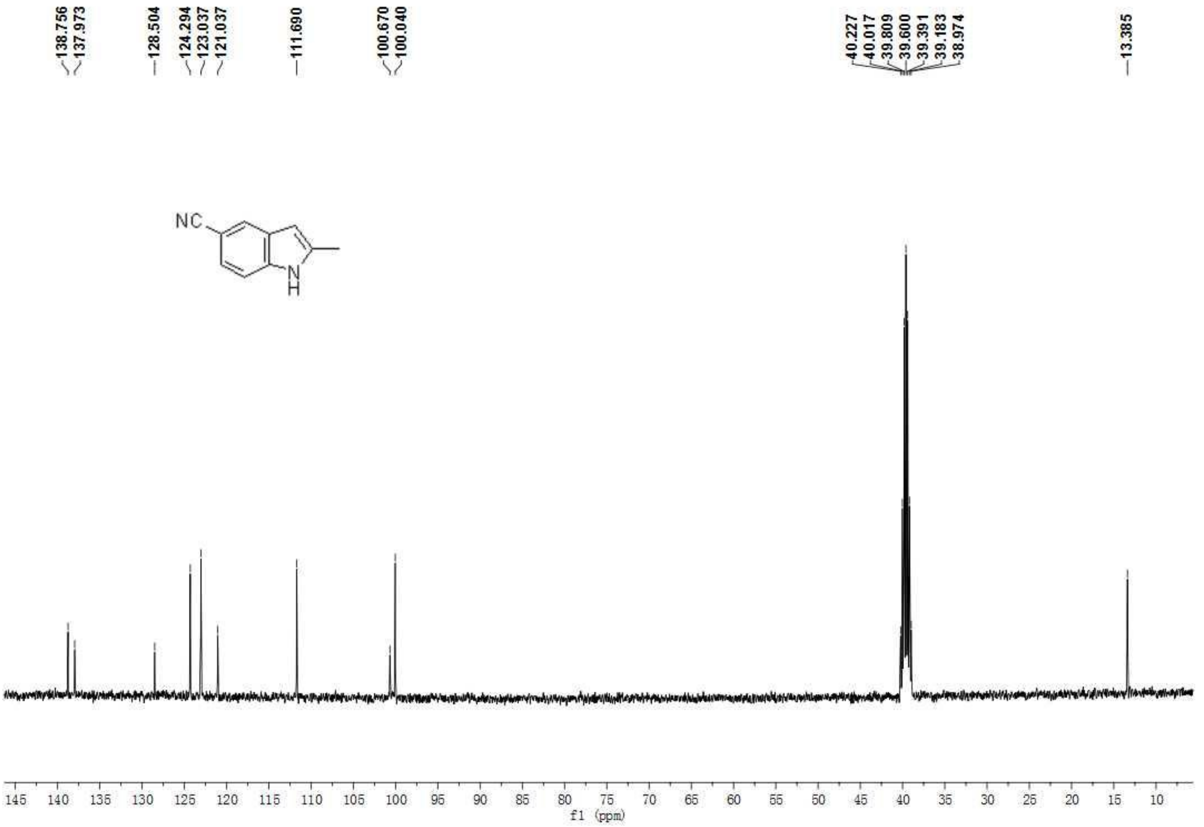

138.756
137.973
128.504
124.294
123.037
121.037
111.690
100.670
100.040
40.227
40.017
39.809
39.600
39.391
39.183
38.974
13.385
NC
N
H
145 140 135 130 125 120 115 110 105 100 95 90 85 80 75 70 65 60 55 50 45 40 35 30 25 20 15 10
f1 (ppm)

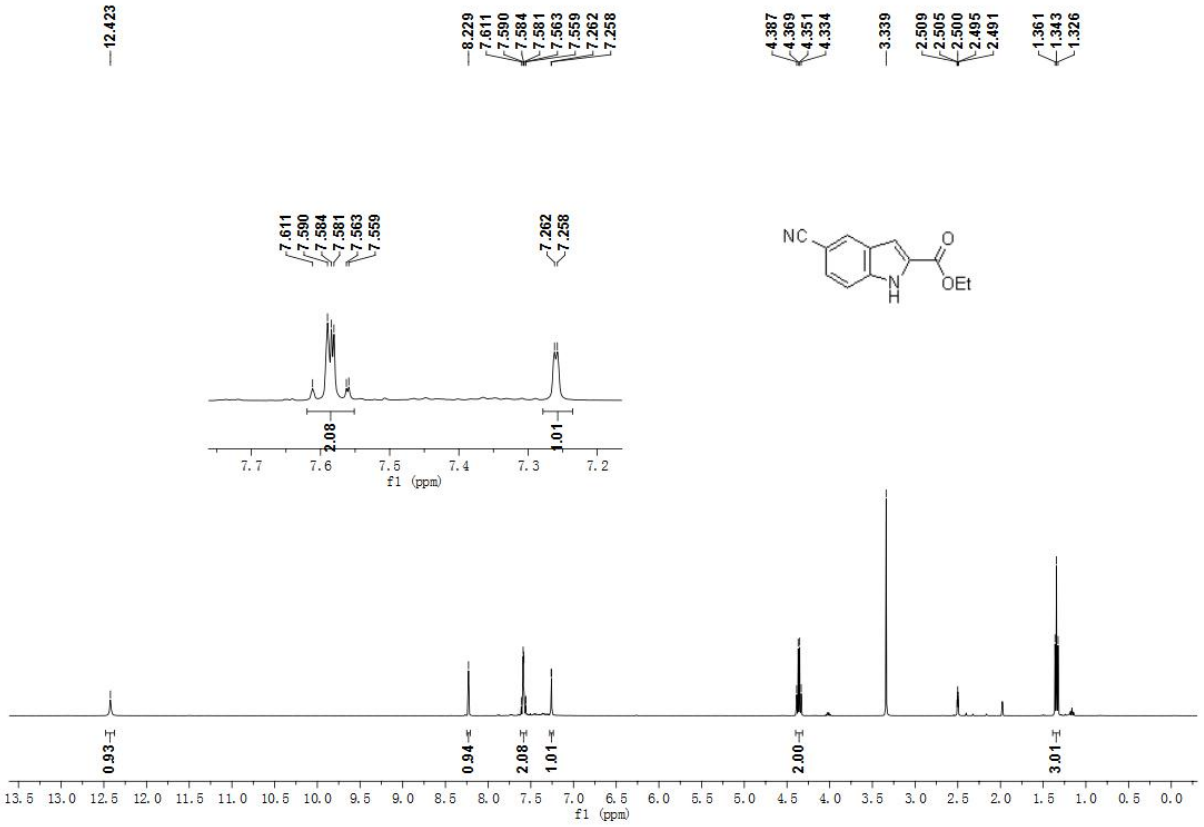

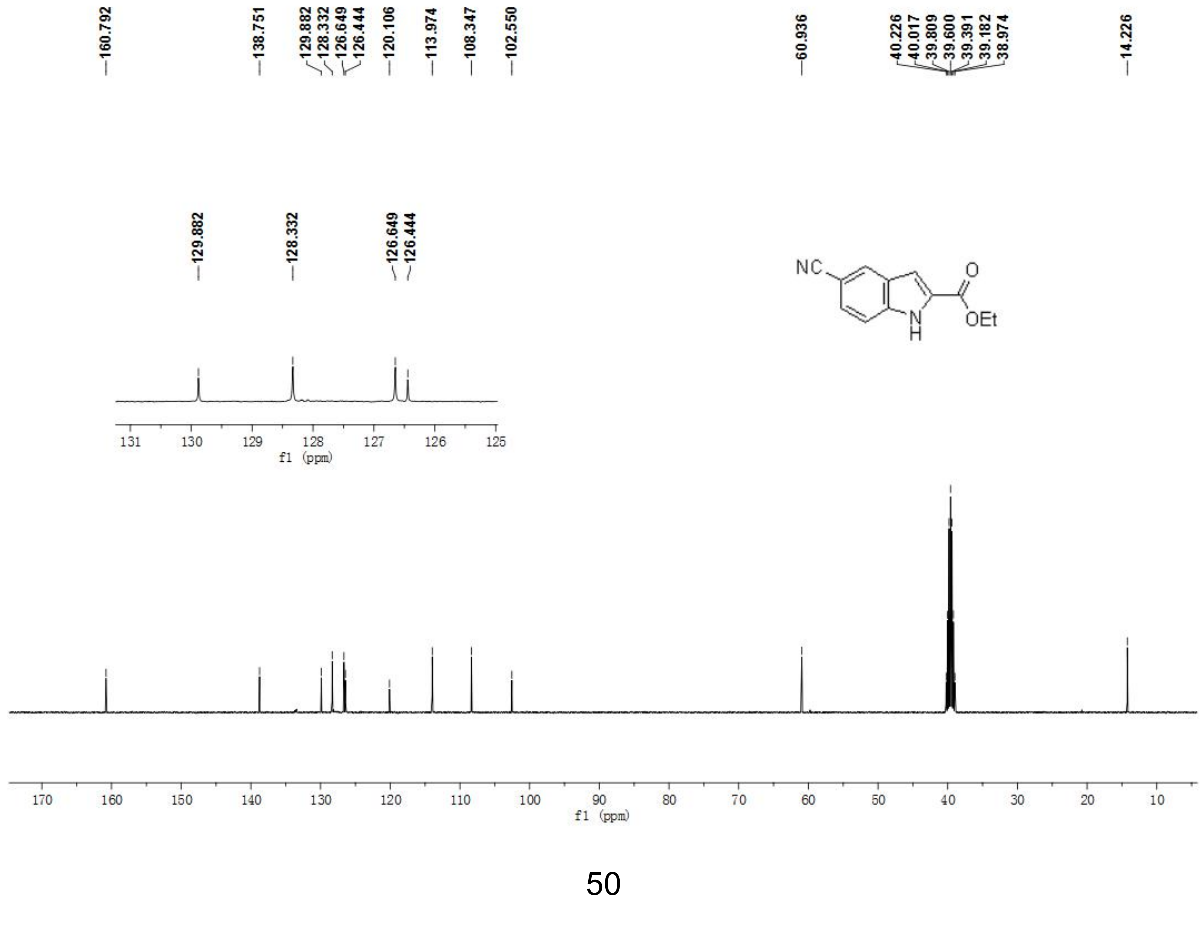

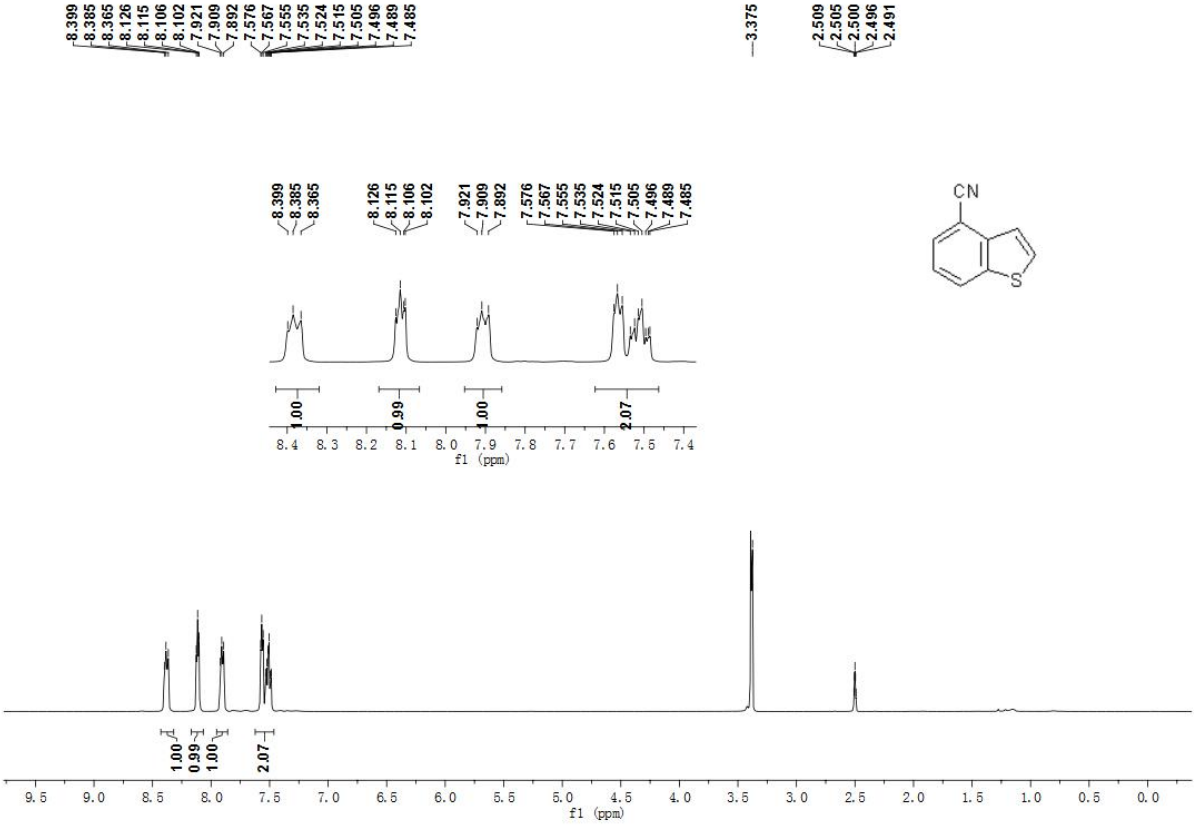
8.399 8.385 8.365 8.126 8.115 8.106 8.102 7.921 7.909 7.892 7.576 7.567 7.555 7.535 7.524 7.515 7.505 7.496 7.489 7.485
3.375
2.509 2.505 2.500 2.496 2.491
CN
S
1.00 0.99 1.00 2.07
f1 (ppm)

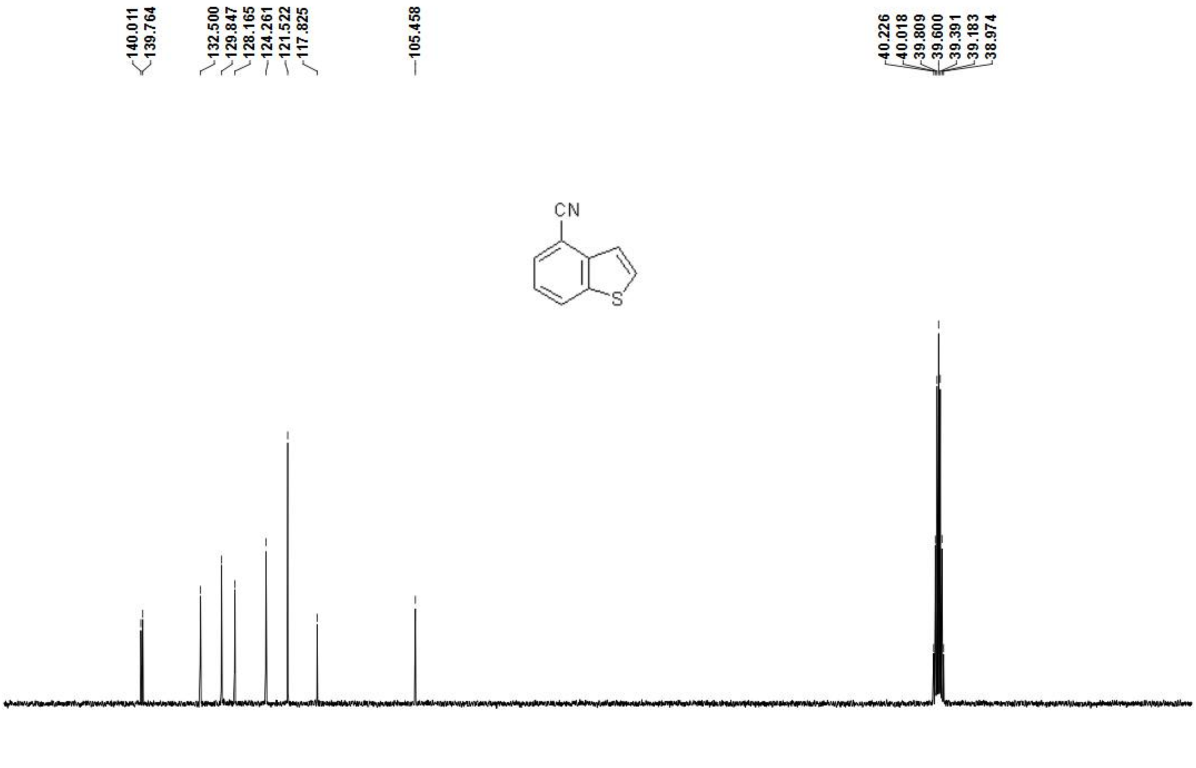
140.011 139.764
132.500 129.847 128.165 124.261 121.522 117.825
105.458
40.226 40.018 39.809 39.600 39.391 39.183 38.974
CN
S
f1 (ppm)

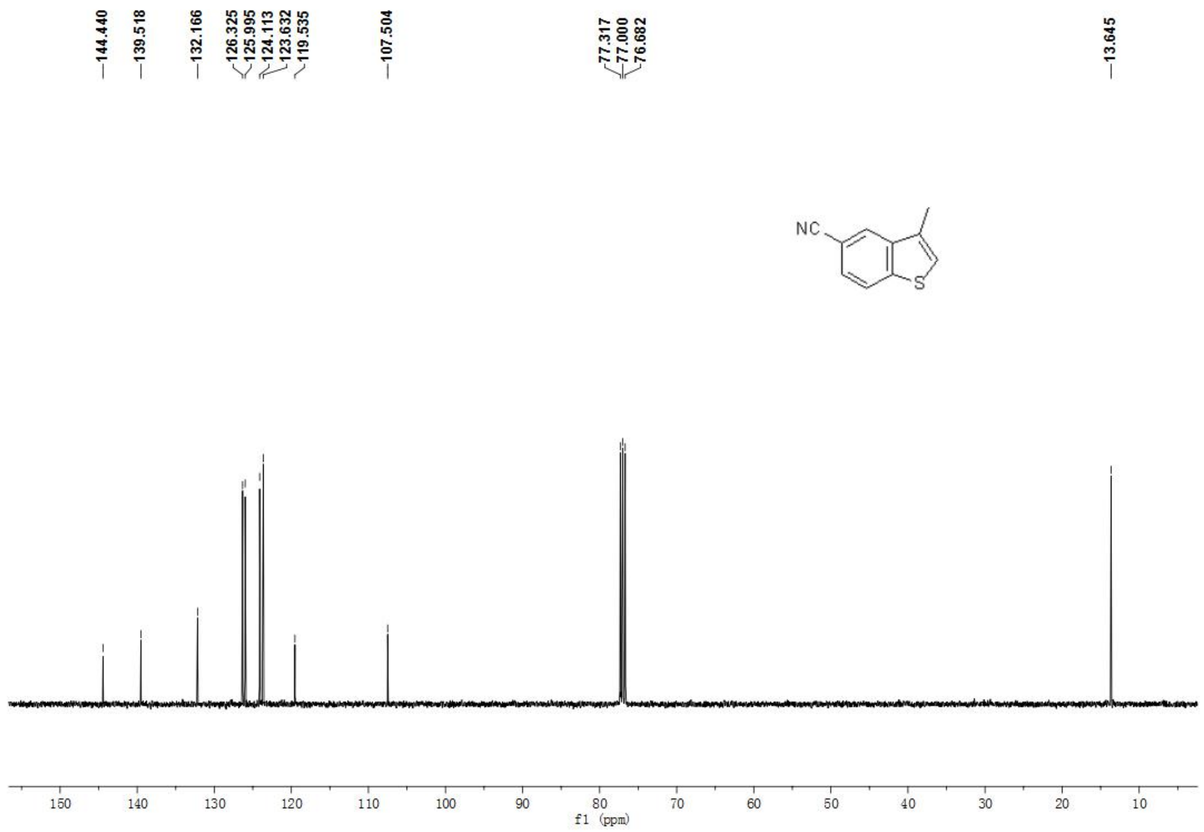

144.440
139.518
132.166
126.325
125.995
124.113
123.632
119.535
107.504
77.317
77.000
76.682
13.645
NC
S
150 140 130 120 110 100 90 80 70 60 50 40 30 20 10
f1 (ppm)

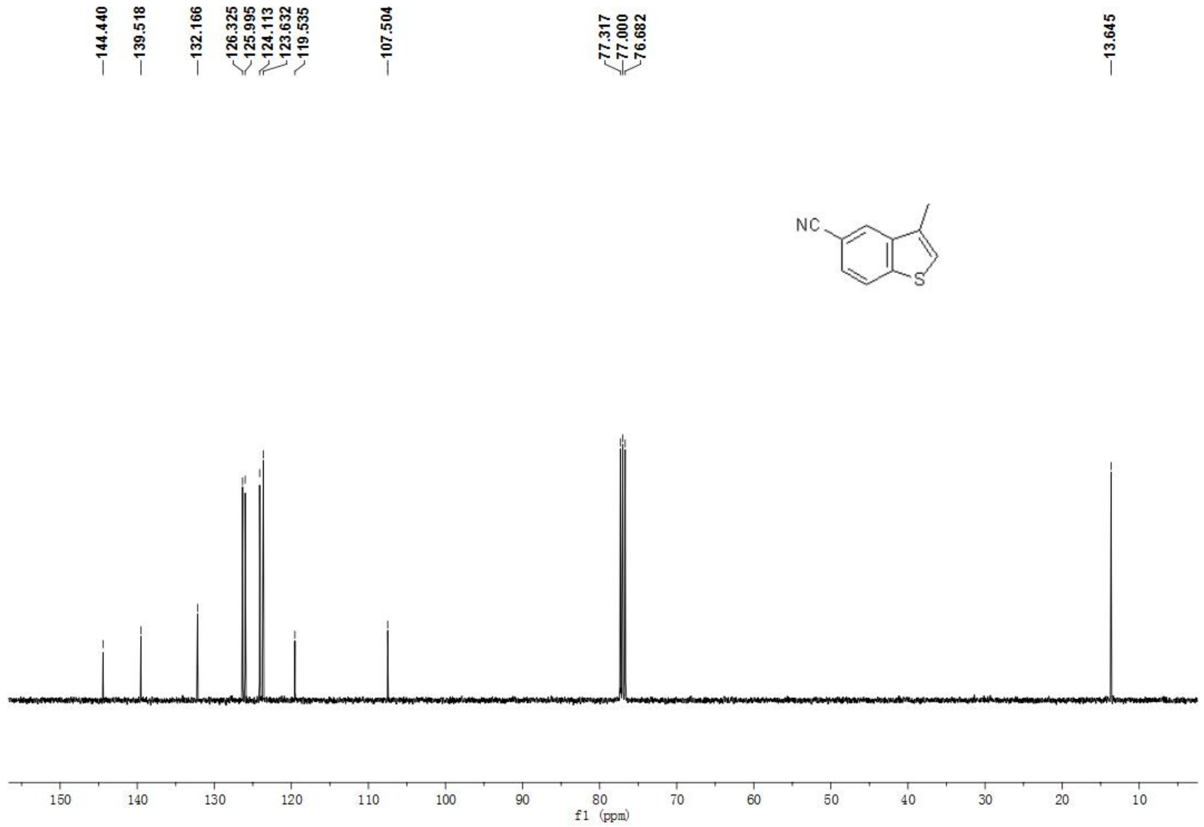

144.440
139.518
132.166
126.325
125.995
124.113
123.632
119.535
107.504
77.317
77.000
76.682
13.645
NC
S
150 140 130 120 110 100 90 80 70 60 50 40 30 20 10
f1 (ppm)

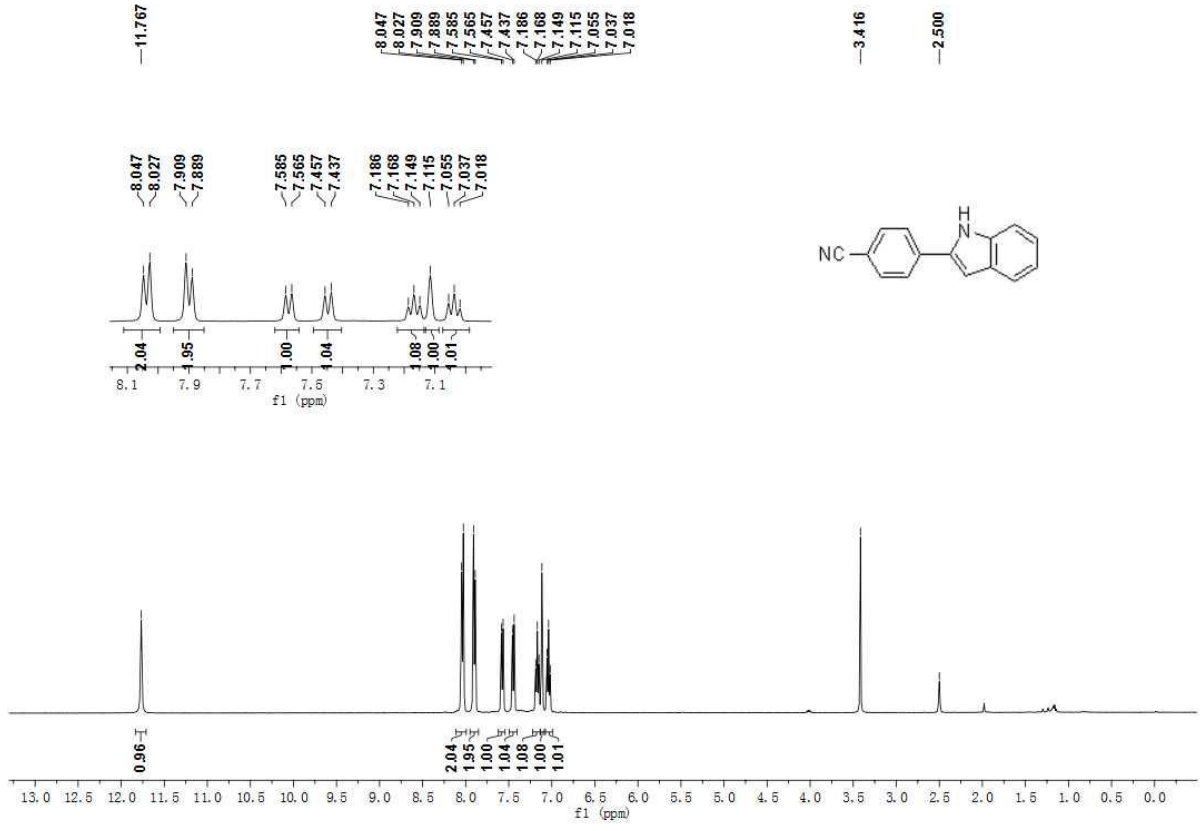

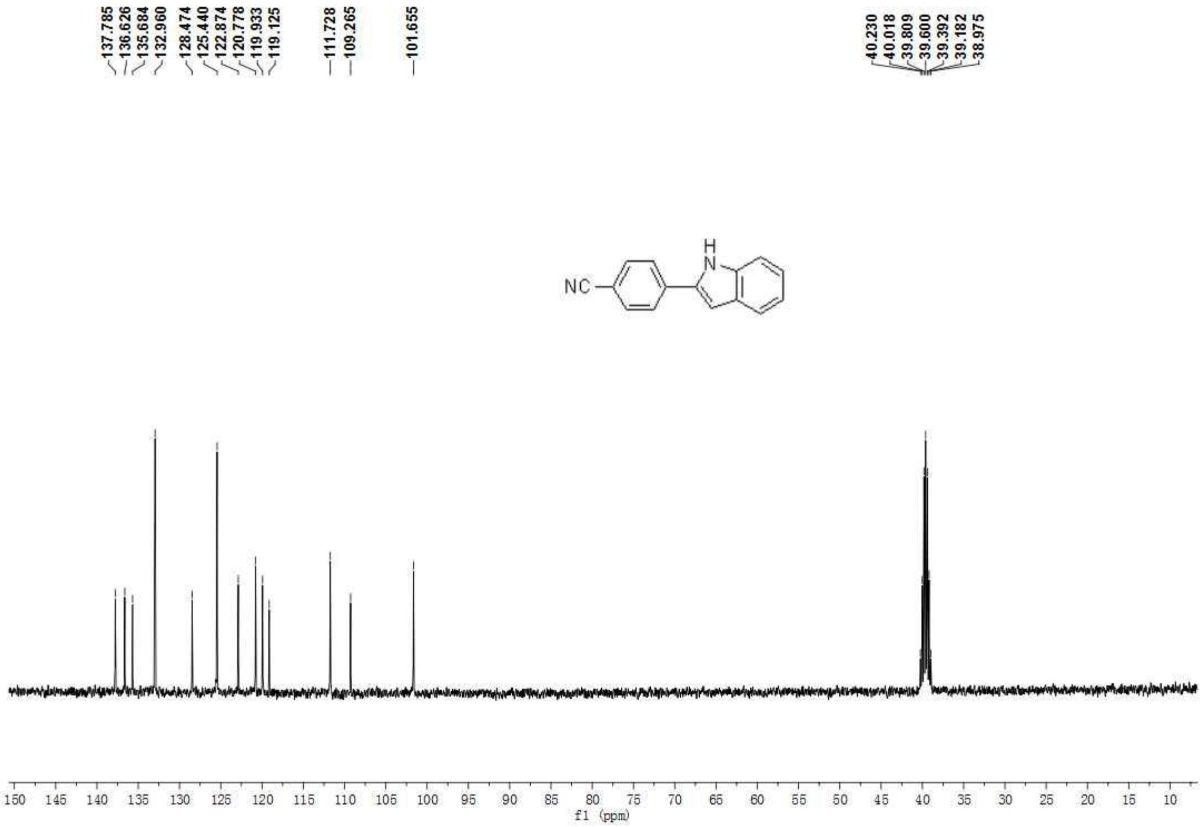

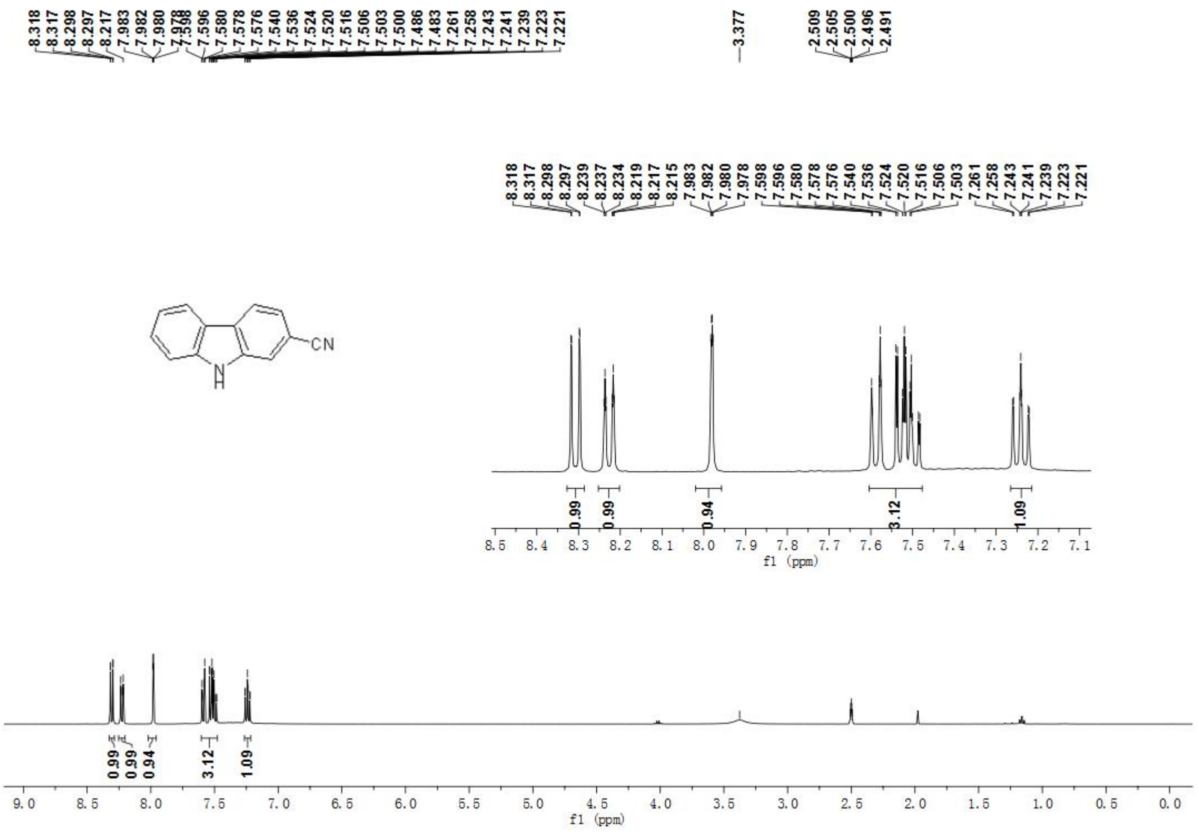

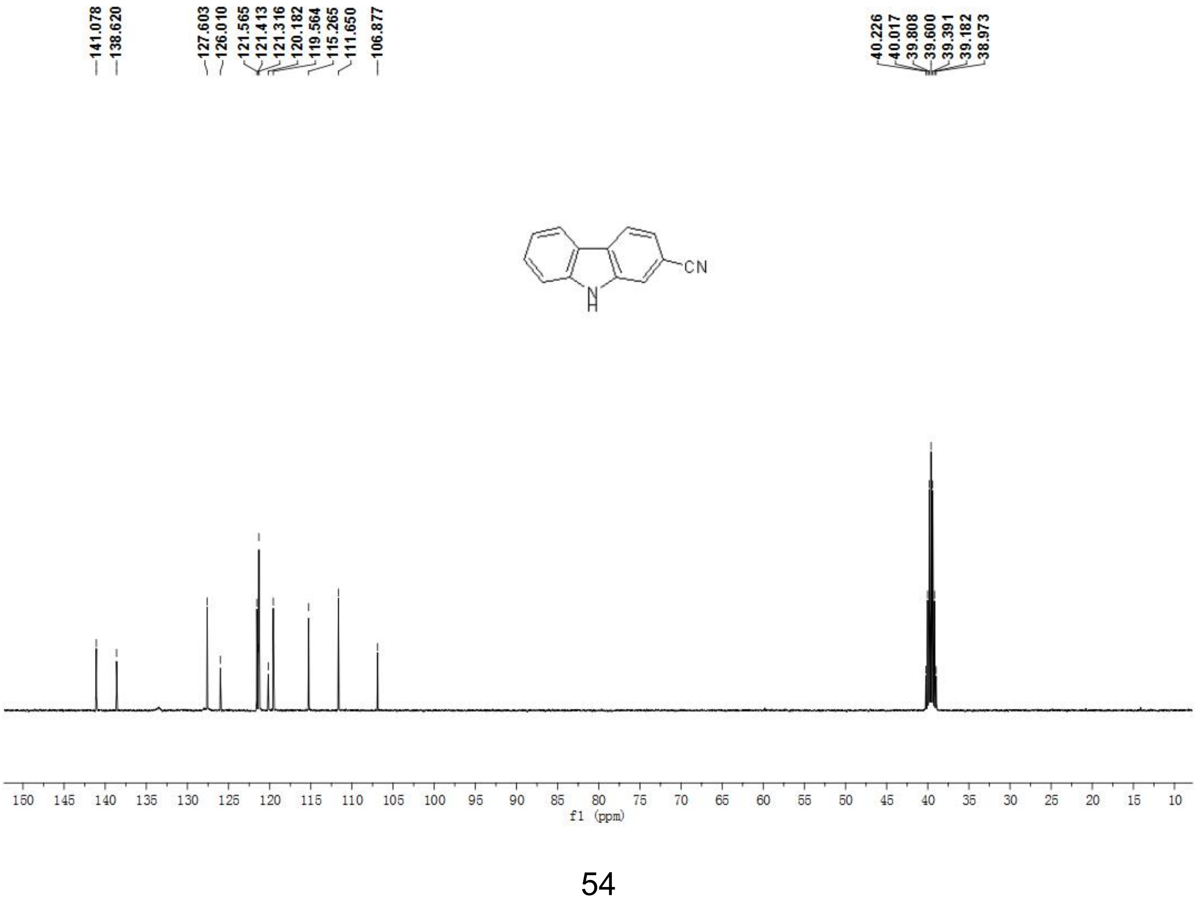

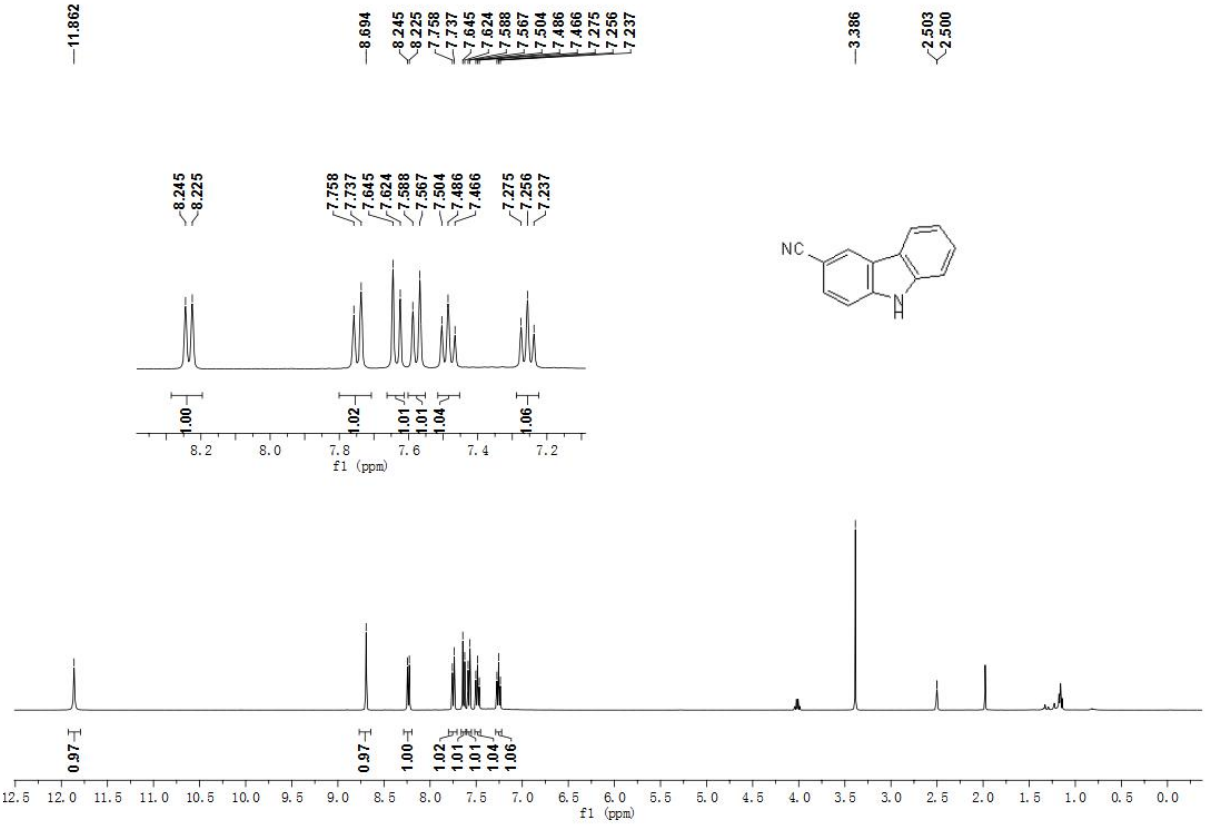
11.862
8.694
8.245
8.225
7.758
7.737
7.645
7.624
7.588
7.567
7.504
7.486
7.466
7.275
7.256
7.237
3.386
2.503
2.500
NC
H
N
1.00
1.02
1.01
1.01
1.04
1.06
0.97
0.97
f1 (ppm)

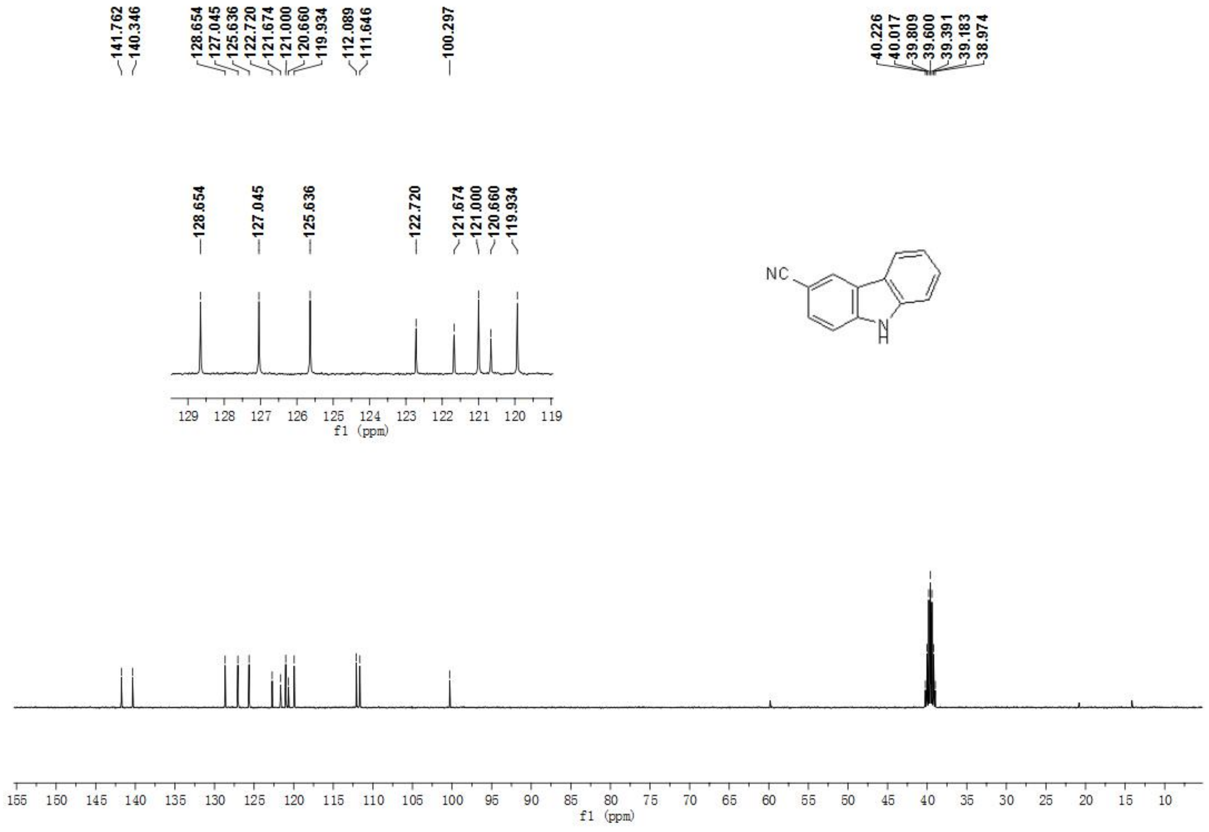
141.762
140.346
128.654
127.045
125.636
122.720
121.674
121.000
120.660
119.934
112.089
111.646
100.297
40.226
40.017
39.809
39.600
39.391
39.183
38.974
NC
H
N
f1 (ppm)

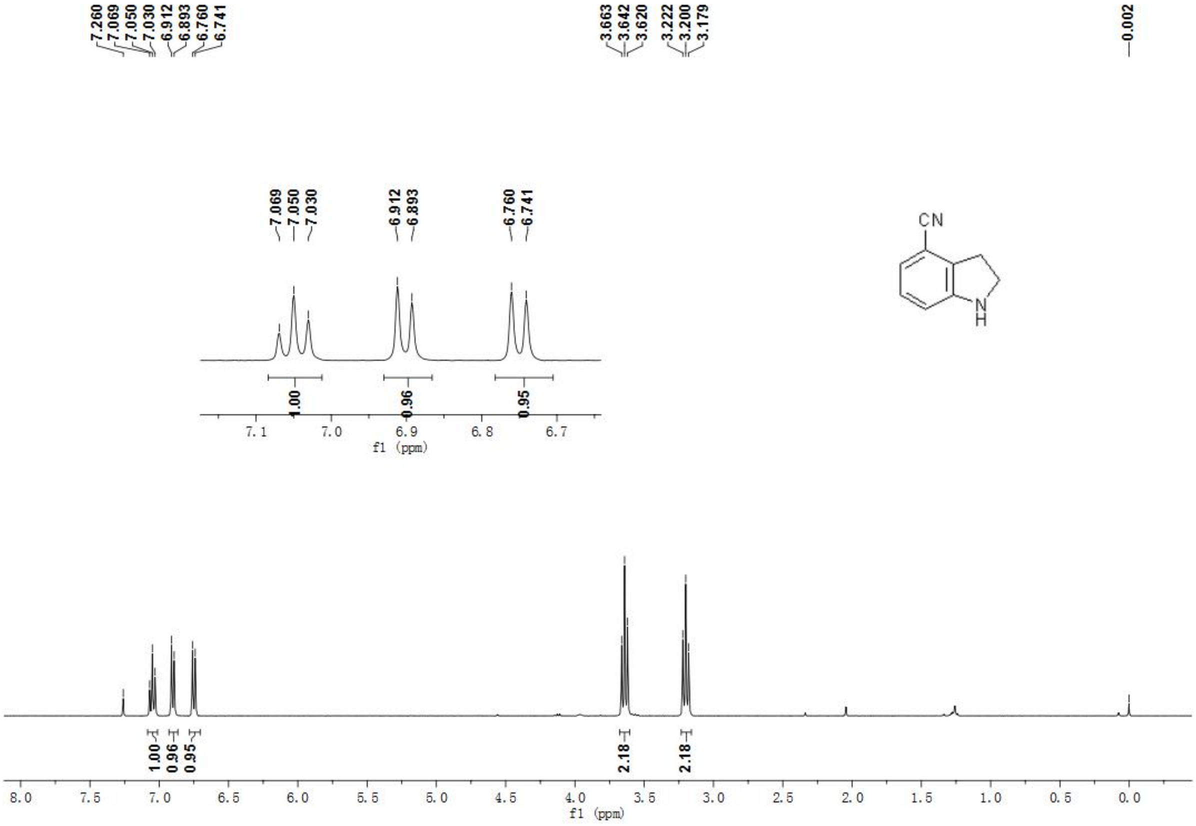
7.260
7.069
7.050
7.030
6.912
6.893
6.760
6.741
3.663
3.642
3.620
3.222
3.200
3.179
0.002
CN
N
H
1.00
0.96
0.95
2.18
2.18
f1 (ppm)

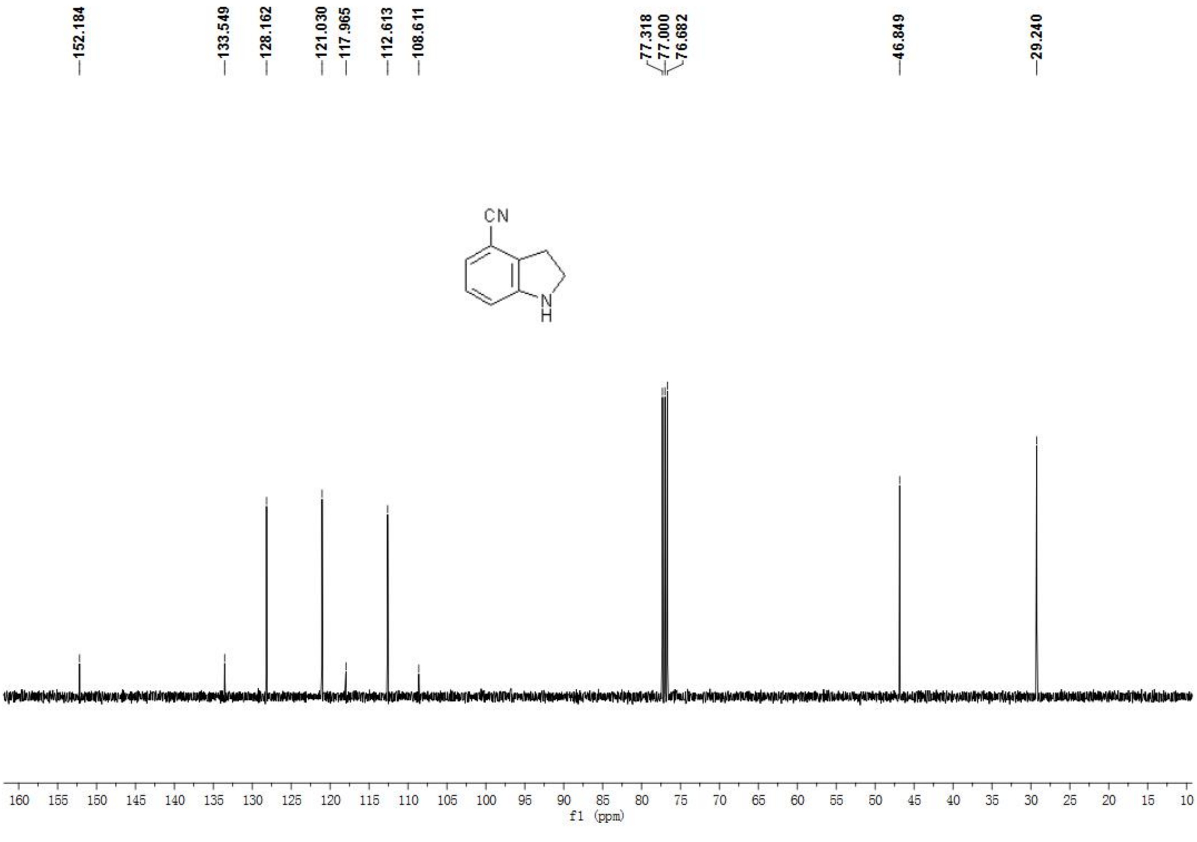
152.184
133.549
128.162
121.030
117.965
112.613
108.611
77.318
77.000
76.682
46.849
29.240
CN
N
H
f1 (ppm)

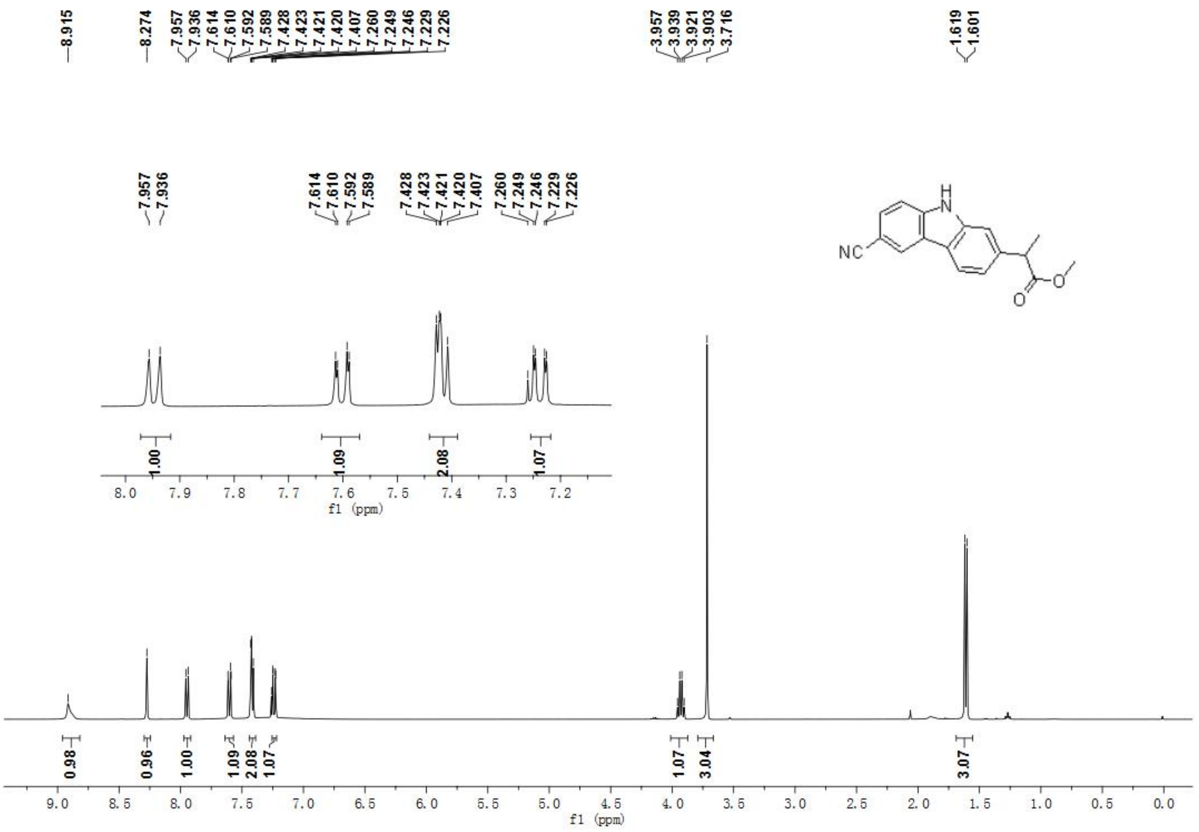

8.915
8.274
7.957
7.936
7.614
7.610
7.592
7.589
7.428
7.423
7.421
7.420
7.407
7.260
7.249
7.246
7.229
7.226
3.957
3.939
3.921
3.903
3.716
1.619
1.601
NC
H
N
O
O
1.00
1.09
2.08
1.07
0.98
0.96
1.00
1.09
2.08
1.07
1.07
3.04
3.07
f1 (ppm)
f1 (ppm)

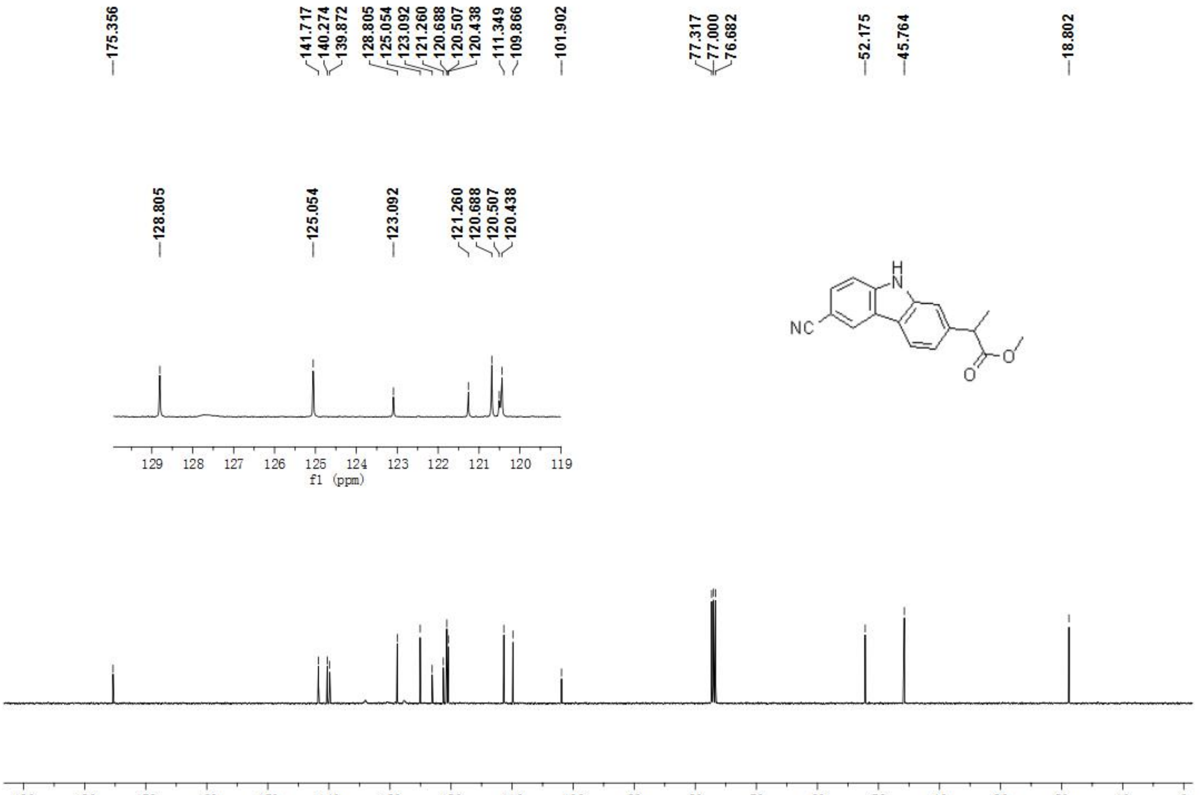

175.356
141.717
140.274
139.872
128.805
125.054
123.092
121.260
120.688
120.507
120.438
111.349
109.866
101.902
77.317
77.000
76.682
52.175
45.764
18.802
128.805
125.054
123.092
121.260
120.688
120.507
120.438
NC
H
N
O
O
f1 (ppm)
f1 (ppm)

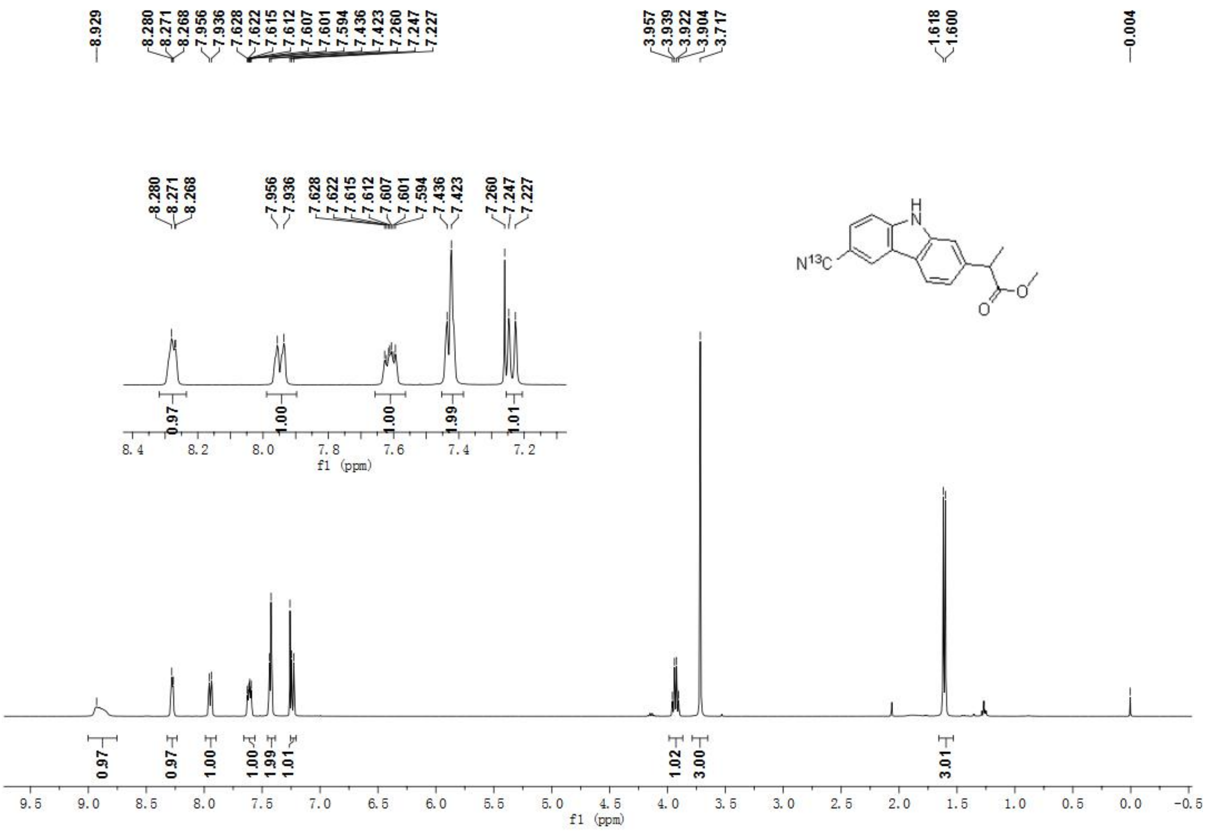

Compound Label	*m/z*	RT	Algorithm	Mass
Cpd 1: 0.335	280.1164	0.335	Find By Formula	279.109

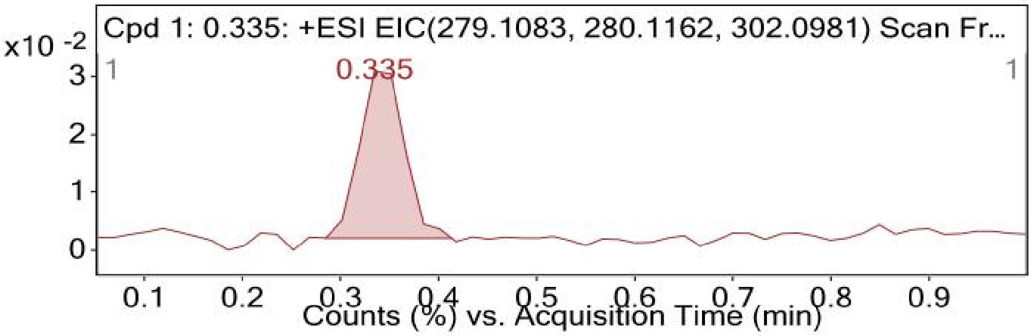

MS Spectrum

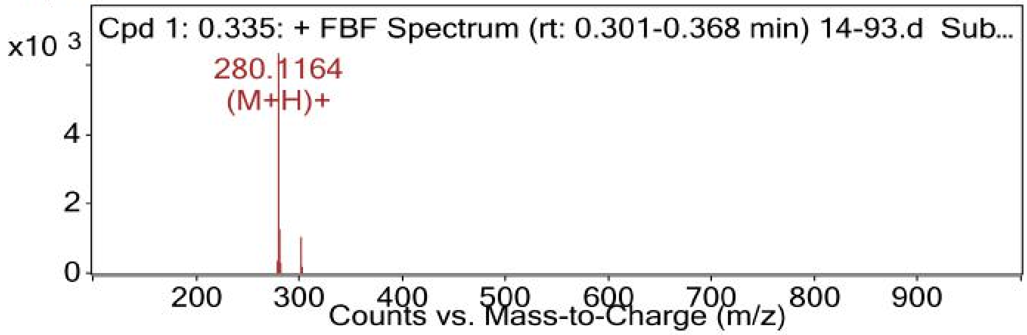

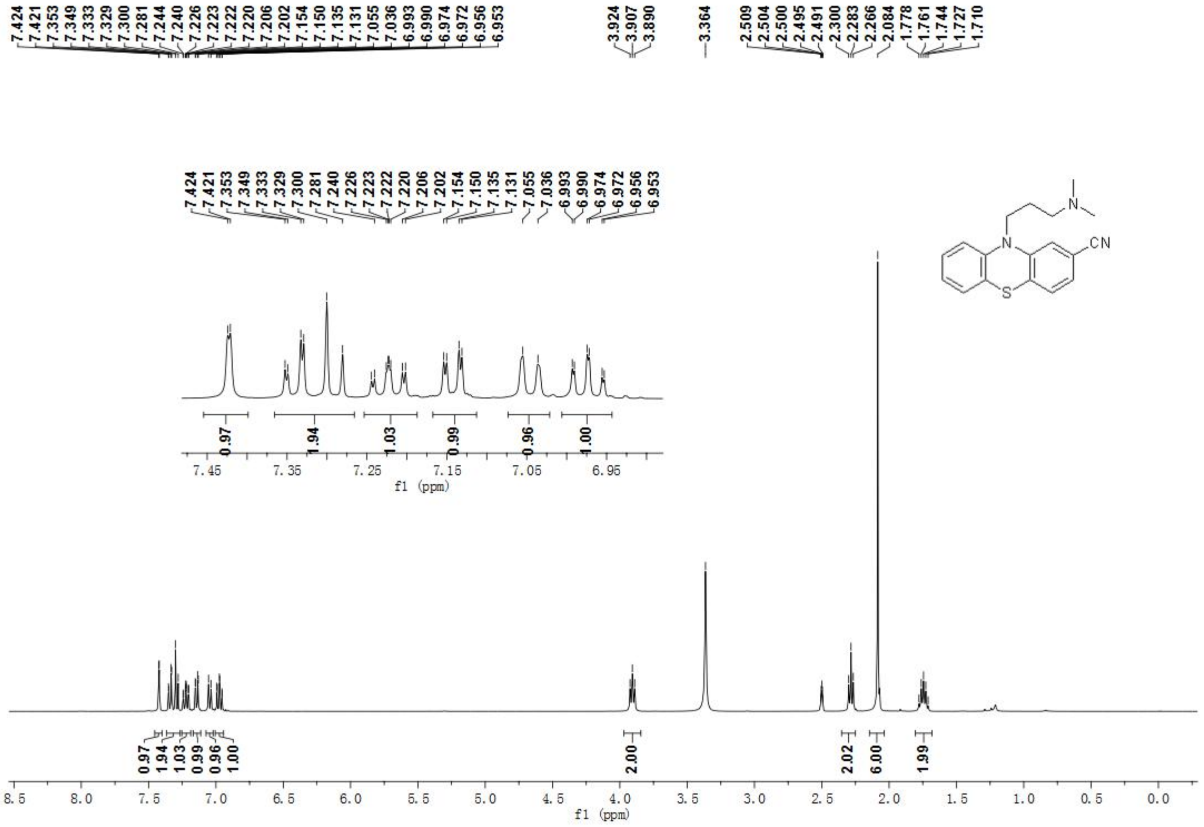
7.424 7.421 7.353 7.349 7.333 7.329 7.300 7.281 7.244 7.240 7.226 7.223 7.222 7.220 7.206 7.202 7.154 7.150 7.135 7.131 7.055 7.036 6.993 6.990 6.974 6.972 6.956 6.953
3.924 3.907 3.890
3.364
2.509 2.504 2.500 2.495 2.491 2.300 2.283 2.266 2.084 1.778 1.761 1.744 1.727 1.710
N
CN
S
0.97 1.94 1.03 0.99 0.96 1.00
7.45 7.35 7.25 7.15 7.05 6.95
f1 (ppm)
2.00 2.02 6.00 1.99
8.5 8.0 7.5 7.0 6.5 6.0 5.5 5.0 4.5 4.0 3.5 3.0 2.5 2.0 1.5 1.0 0.5 0.0
f1 (ppm)

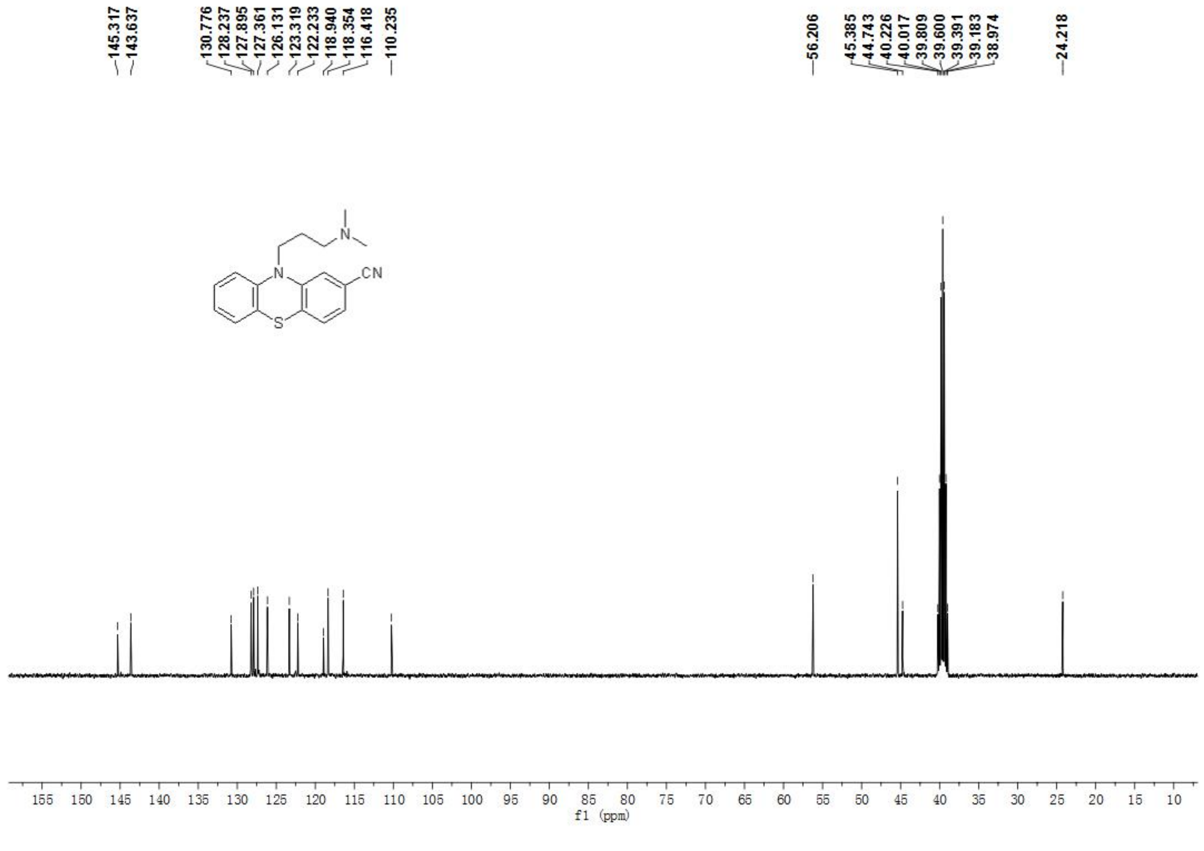
145.317 143.637
130.776 128.237 127.895 127.361 126.131 123.319 122.233 118.940 118.354 116.418 110.235
56.206
45.385 44.743 40.226 40.017 39.809 39.600 39.391 39.183 38.974
24.218
N
CN
S
155 150 145 140 135 130 125 120 115 110 105 100 95 90 85 80 75 70 65 60 55 50 45 40 35 30 25 20 15 10
f1 (ppm)

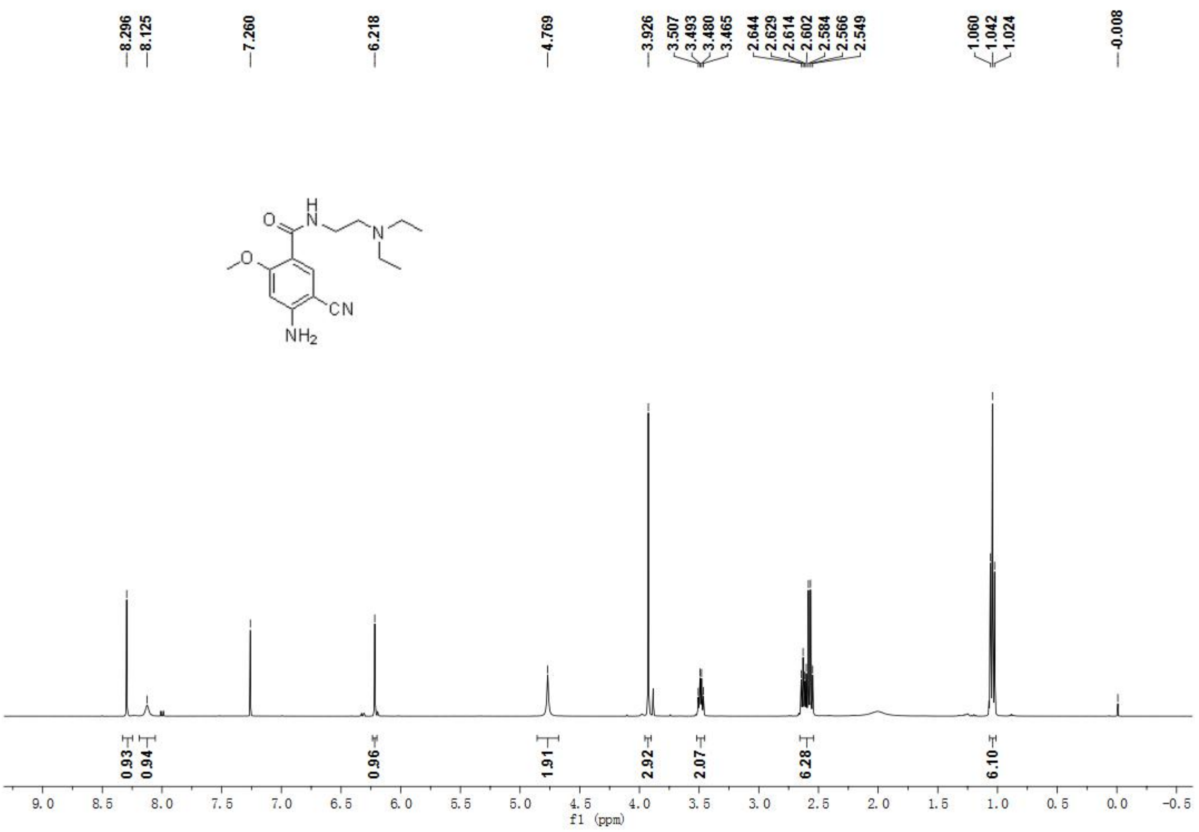
8.296
8.125
7.260
6.218
4.769
3.926
3.507
3.493
3.480
3.465
2.644
2.629
2.614
2.602
2.584
2.566
2.549
1.060
1.042
1.024
-0.008
O
H
N
N
O
CN
NH2
0.93
0.94
0.96
1.91
2.92
2.07
6.28
6.10
f1 (ppm)

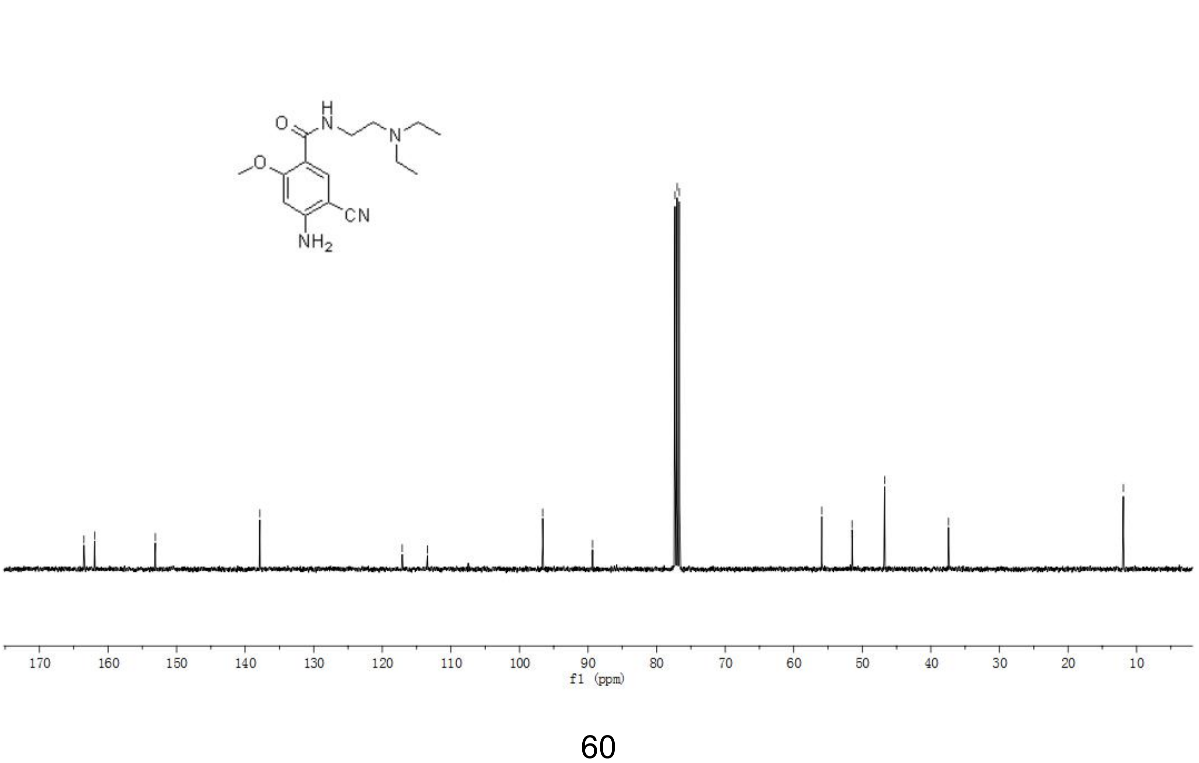
163.505
161.951
153.096
137.861
117.093
113.424
96.608
89.335
77.318
77.000
76.683
55.906
51.487
46.742
37.428
11.925
O
H
N
N
O
CN
NH2
f1 (ppm)

Supplementary References

1. Miedaner, A., Haltiwanger, R. C. & DuBois, D. L. Relationship between the bite size of diphosphine ligands and tetrahedral distortions of "Square-Planar" Nickel(II) complexes: stabilization of Nickel(I) and Palladium(I) complexes using diphosphine ligands with large bites. *Inorg. Chem*, **30**, 417-427 (1991).

2. Heintz, R. A., Smith, J. A., Szalay, P. S., Weisgerber, A., Dunbar, K. R., Beck, K. & Coucouvanis, D. Inorganic Syntheses: useful reagents and ligands; John Wiley & Sons, Inc. **2002**; pp 75-83.

3. Chavez, C. A., Choi, J. & Nesterov, E. E. One-step simple preparation of catalytic initiators for catalyst-transfer Kumada polymerization: synthesis of defect-free polythiophenes. *Macromolecules*, **47**, 506-516 (2014).

4. Standley, E. A., Smith, S. J., Müller, P. & Jamison, T. F. A broadly applicable strategy for entry into homogeneous Nickel(0) catalysts from air-stable Nickel(II) Complexes. *Organometallics*, **33**, 2012-2018 (2014).

5. Zhang, X., Xia, A., Chen, H. & Liu, Y. General and mild Nickel-catalyzed cyanation of aryl/heteroaryl chlorides with $Zn(CN)_2$: key roles of DMAP. *Org. Lett.* **19**, 2118-2121 (2017).

6. Gray, L. R., Higgins, S. J., Levason, W. & Webster, M. Co-ordination chemistry of higher oxidation states. Part 8. Nickel(III) complexes of bi- and multi-dentate phosphorus and arsenic ligands; crystal and molecular structure of $[Ni(Ph_2PCH_2CH_2PPh_2)Br_3]C_6H_5Me$. *J. Chem. Soc., Dalton Trans.* 459-467 (1984).

7. Zhang, G. Y., Ren, X. Y., Chen, J., Hu, M. & Cheng, J. Copper-mediated cyanation of aryl halide with the combined cyanide source. *Org. Lett.* **13**, 5004-5007 (2011).

8. Kim, J., Choi, J., Shin, K. & Chang, S. Copper-mediated sequential cyanation of aryl C-B and arene C-H bonds using ammonium iodide and DMF. *J. Am. Chem. Soc.* **134**, 2528-2531 (2012).

9. Yang, L., Liu, Y.-T., Park, Y., Park, S.-W. & Chang, S. Ni-mediated generation of "CN" unit from formamide and its catalysis in the cyanation reactions. *ACS Catal.* **9**, 3360-3365 (2019).
